# The Resistome, Mobilome, Virulome and Phylogenomics of Multidrug-Resistant *Escherichia coli* Clinical Isolates from Pretoria, South Africa

**DOI:** 10.1038/s41598-019-52859-2

**Published:** 2019-11-11

**Authors:** Nontombi Marylucy Mbelle, Charles Feldman, John Osei Sekyere, Nontuthuko Excellent Maningi, Lesedi Modipane, Sabiha Yusuf Essack

**Affiliations:** 1 Department of Medical Microbiology, Faculty of Health Sciences, University of Pretoria, Pretoria, South Africa; 2 National Health Laboratory Service, Tshwane Academic Division, Pretoria, South Africa; 3 Department of Internal Medicine, Faculty of Health Sciences, University of the Witwatersrand, Johannesburg, South Africa; 4 Antimicrobial Research Unit, College of Health Sciences, University of KwaZulu/Natal, Durban, South Africa

**Keywords:** Computational biology and bioinformatics, Evolution, Genetics, Microbiology, Molecular biology

## Abstract

Antibiotic-resistant *Escherichia coli* is a common occurrence in food, clinical, community and environmental settings worldwide. The resistome, mobilome, virulome and phylogenomics of 20 multidrug resistant (MDR) clinical *E. coli* isolates collected in 2013 from Pretoria, South Africa, were characterised. The isolates were all extended-spectrum β-lactamase producers, harbouring CTX-M (n = 16; 80%), TEM-1B (n = 10; 50%) and OXA (n = 12, 60%) β-lactamases alongside genes mediating resistance to fluoroquinolones, aminoglycosides, tetracyclines etc. Most resistance determinants were found on contigs containing IncF plasmid replicons and bracketed by composite transposons (Tn3), diverse ISs and class 1 integrons (In13, In54, In369, and In467). Gene cassettes such as *bla*_OXA,_ *dfrA5-psp-aadA2-cmlA1a-aadA1-qac* and *estX3-psp-aadA2-cmlA1a-aadA1a-qac* were encompassed by Tn*3* and ISs; several isolates had same or highly similar genomic antibiotic resistance islands. ST131 (n = 10), ST617 (n = 2) and singletons of ST10, ST73, ST95, ST410, ST648, ST665, ST744 and ST998 clones were phylogenetically related to clinical (human and animal) strains from Egypt, Kenya, Niger, Nigeria, Tanzania, and UK. A rich repertoire of virulence genes, including *iss, gad and iha* were identified. MDR *E. coli* harbouring chromosomal and plasmid-borne resistance genes in same and multiple clones exist in South Africa, which is very worrying for clinical epidemiology and infectious diseases management.

## Introduction

The rising prevalence of multi-drug resistant (MDR) bacteria has been attributed to increasing antibiotic use, as well as poor infection control in healthcare, farm, and community settings^1–3^. Due to the ubiquity of *Escherichia coli* in community, hospital, farm/food and environmental settings as well as in the gastrointestinal tract of humans and animals, they are used as index species to monitor the prevalence, types, and movement of resistance genes within and between clinical, farm, community and environmental settings^4–6^. Furthermore, the ability of *E. coli* to exchange genetic material with other bacterial species make them ideal candidates for studying the reservoir of resistance genes in any setting^7,8^. Of equally grave concern is the presence of toxigenic and diarrheagenic *E. coli* strains that cause diarrhoea and substantial mortalities in several populations worldwide^4,5,9–11^.

Antibiotic-resistant *E. coli* has been described in foods, farms, animals, the environment and in clinical settings in South Africa, with varying resistance to colistin (*mcr-1*), carbapenems (NDM, OXA, KPC, IMP, and VIM), cephalosporins (TEM, SHV, OXA, and PER), fluoroquinolones (OqxAB, QnrA/B/C/S, AAC(6′)-Ib-cr, qepA, and mutations in *parCE* and *gyrAB*), tetracyclines (*tetA/B/C*), aminoglycosides etc.^3,12–17^. A recent report on third-generation cephalosporins and ciprofloxacin resistance in *E. coli* respectively found 27% and 30% resistance rates (Personal communication, Olga Perovic, 2019). As well, a study by Habte *et al*. (2009) on hospital- and community-acquired uropathogens found *E. coli* to be the most common (39%), and were mostly found to be producers of extended-spectrum β-lactamases (ESBLs)^18^.

Globally, the spread of antibiotic resistance (ABR) has been attributed to the lateral transfer of genetic material^19–23^. Owing to the community- and healthcare-associated infections caused by *E. coli*, the exchange of multi-drug resistance in this species is of particular importance^9,12^. Mobile genetic elements (MGEs) enable the transfer of ABR genes in *Enterobacteriaceae*^20–22^. Several MGEs have been described, including plasmids, which are circular, extra-chromosomal segments of DNA that can acquire insertion sequences, integrons and transposons to disseminate resistance genes^24^. Integrons are genetic elements characterised by an *int* integrase site-specific gene, an *att1* recombination site and a *p* promoter gene that enables the transcription of cassettes, captured by the integron. Class 1 integrons are the most common and have been described in approximately 10% of sequenced bacterial genomes^25^. Gene cassettes are within integrons, usually consisting of single, but sometimes multiple genes associated with resistance to more than one class of antibiotics^26,27^. Integrons themselves are not mobile, lacking functions for self-mobility, and can be either chromosomal integrons, when found on the bacterial chromosome, or mobile integrons, when transposed on or associated with plasmids^28^.

MGEs and ABR genes associated with *E. coli* have been well described globally but few studies have described the relationship between circulating MGEs and ABR genes in *E. coli* isolates from Africa, particularly South Africa. This study used whole-genome sequencing to identify MGEs, including integrons, plasmids and cassette arrays associated with MDR *E. coli* isolates in South Africa, determining their phylogenetic relationship with other South Africa, African and global isolates.

## Materials and Methods

### Bacterial isolates

The study sample consisted of twenty consecutive MDR *E. coli* isolates that were collected between April and November 2013 as part of a larger study where ESBL-producing Enterobacteriaceae, co-resistant to fluoroquinolones and aminoglycosides were collected from a referral laboratory that serves at least two secondary and three tertiary academic hospitals within the Gauteng province, South Africa. These isolates were collected from blood (n = 5), urine (n = 11), and unknown sources (n = 4) from patients having bacterial infections.

### Identification and antimicrobial susceptibility testing

The isolates were isolated after growing them on blood agar and subsequently on Mueller-Hinton agar at 37 °C for 24 hours. They were then screened for ESBL production using cefoxitin, ceftazidime, and clavulanic acid antibiotic discs on Mueller-Hinton agar plates according to already reported protocols^6^. The species and antimicrobial sensitivity of the isolates were determined with the MicroScan WalkWay7465 (Beckman Coulter California USA) using antibiotic panels involving 32 antibiotics: penicillins, cephems, carbapenems, polymyxins, fluoroquinolones, aminoglycosides, tetracyclines, tigecycline, sulphamethoxazole-trimethoprim, nitrofurantoin and fosfomycin (Table S1). The MICs were interpreted according to the CLSI guidelines (CLSI M100 29^th^ Ed., 2019)^29^, except for antibiotics such as colistin and tigecycline for which EUCAST (2019) breakpoints were used due to the absence of CLSI breakpoints^30–32^. The identification of the species was confirmed by the NCBI’s ANI (average nucleotide identity) database.

### Analysis of whole genome sequence data

Whole-genome sequencing (WGS) was performed on the Ion torrent (Covaris, USA) and the Illumina Miseq (San Diego, USA) systems using already described methods^21,33,34^. Briefly, the genomic DNA of the isolates were extracted and sheared to 200-bp libraries; 280-bp (for Ion Proton) and 350 bp (for Illumina Miseq) fragments were selected using 2% agarose gels and Pippen prep (Sage Science, Beverly, MA, USA). Individual libraries were pooled and sequenced on the Ion Proton (ThermoFisher, Waltham, MA, USA) or Illumina Miseq (San Diego, USA). The generated raw reads were de novo assembled using the SPAdes assembler.

Assembled sequences were annotated using ResFinder (https://cge.cbs.dtu.dk/services/ResFinder/) at default threshold ID (90%) and minimum length (60%) values to identify resistance genes. MLST 2.0 (https://cge.cbs.dtu.dk/services/MLST/) was used to identify the sequence types of the isolates. The INTEGRALL database (http://integrall.bio.ua.pt/) was used to identify integrons and gene cassettes within the genomic sequences. NCBI’s PGAP^35^, ISFinder (https://isfinder.biotoul.fr/) and the RAST SEEDVIEWER (http://rast.nmpdr.org/seedviewer.cgi) were used to annotate and identify the insertion sequences (ISs) and transposons bracketing the resistance genes. PlasmidFinder 2.1 (https://cge.cbs.dtu.dk/services/PlasmidFinder/) and pMLST 2.0 (https://cge.cbs.dtu.dk/services/pMLST/) were used to identify the plasmid replicons and incompatibility groups on the various contigs. The sequences have been deposited at GenBank under the Bioproject PRJNA355910, with the individual accession numbers delineated in Table 1, S1 and S2. Mutations in *gyrAB, parCE, mgrB, pmrAB*, and *phoPQ* were manually curated from NCBI’s BLAST by comparing the genomes of the isolates to wild type reference sequences^14^.

**Table 1:** Patient demographics and resistance, virulence and plasmid replicon genes in the Escherichia coli strains.

Strain	Accession number	Age (yrs)	Gender	Referral hospital	Specimen	ESBL*	MLST^†^	Resistance genes	Virulence genes	Plasmids	pMLST^‡^
E003	NXIZ00000000	ND^§^	M**	Tshwane Academic	Blood	+^††^	ST-744	*strA, aadA5, strB, sul2, sul1, dfrA17, tet(B), catA1, blaCMY-2*	*tsh, mchF, iroN, iss, gad*	IncQ1	IncF[F16:A-:B1], IncI1[Unknown ST]
E005	NXLF00000000	3	M	Kalafong	Blood	+	ST-131	*strA, aac(3)-IIa, strB, aadA5, aac(3)-IId, mph(A), sul1, sul2, dfrA17, tet(A), catB3, blaCTX-M-15, blaTEM-1B, blaOXA-1, aac(6’)Ib-cr*	*Iha, gad, iss, ccI, senB, sat*	IncY, ColRNAI, Col156, IncX4, Col(MG828)	IncF[F2:A2:B-]
E009	NXLH00000000	3	M	Kalafong	Blood	+	ST-131	*strA, aac(3)-IId, strB, aadA5, sul1, sul2, dfrA17, tet(A), catB3, blaCTX-M-15, blaTEM-1B, blaOXA-1, aac(6’)Ib-cr*	*Iha, gad, iss, ccI, senB, sat*	IncY, ColRNAI, Col156, IncX4, Col(MG828)	IncF[F2:A2:B-]
E011	NXKR00000000	7	M	Tshwane Academic	Urine	+	ST-131	*strA, aadA5, strB, aac(3)-IIa, mph(A), sul2, sul1, dfrA14, dfrA17, catB3, blaCTX-M-15, blaOXA-1, aac(6’)Ib-cr*	*cnf1, iha, iss, gad*	IncR, IncB/O/K/Z, Col156, ColRNAI	IncF[F31:A4:B1]
E013	NXIN00000000	63	F^‡‡^	Kalafong	Urine	+	ST-131	*strA:strB:sul2:tet(A), blaCTX-M-27*	*sat, iss, iha, gad*	Col(BS512), Col156Col(MG828)	IncF[F1:A2:B-]
E019	NXLG00000000	66	F	Tshwane Academic	ND	+	ST-617	*aadA5, aac(3)-IId, strA, strB, mph(A), sul1, sul2, dfrA17, tet(B), catB3, catA1, blaTEM-1B, blaCTX-M-15, blaOXA-1, aac(6’)Ib-cr*	*Iss, gad*	Col8282, ColRNAI	IncF[F22*:A4:B1]
E020	NXJB00000000	66	F	Tshwane Academic	ND	+	ST-617	*strA, aadA5, aac(3)-IId, strB, mph(A), sul2, sul1, dfrA17, tet(B), catB3, catA1, blaCTX-M-15, blaTEM-1B, blaOXA-1, aac(6’)Ib-cr*	*iss, gad*	Col8282, ColRNAI	IncF[F22*:A4:B1]
E021	NXIO00000000	12	F	Tshwane Academic	Urine	+	ST-131	*aadA5, aac(3)-Ila, strA:strB, dfrA17:sul1:mph(A), sul2:tet(A), blaCTX-M-15, aac(6’)Ib-cr:blaOXA-1:catB3*	*gad, iha, iss, senB, gad, iha, iss, senB*	Col156, Col(MG828)	IncF[F1:A1:B16]
E035	NXJC00000000	29	F	Kalafong	Urine	+	ST-10	*aadA1, strA, strB, aph(3’)-IIa, aadA2, sul1, oqxA, oqxB, dfrA12, tet(A), tet(B), cmlA1, blaTEM-1B*	*katP, cba, aaiC, astA, astA, gad*	ColRNAI	IncHI2[ST-3], IncN[ST-1], IncF[F29:A-:B24]
E040	NXIP00000000	68	F	Tshwane Academic	Urine	+	ST-95	*fosA, aph(3’)-IIa, aadA1:cmlA1:aadA2, strA:strB, sul2, sul3, dfrA5, blaCTX-M-14, blaTEM-1B*	*mchF, ireA, gad, vat, iroN, iss, iss*	IncQ1, Col156, Col(MG828)	IncF[F2:A-:B1], IncHI2[ST-3]
E053	NXIR00000000	53	F	Kalafong	Urine	+	ST-73	*strA:strB:sul2, aac(3)-IIa, aph(3’)-Ia, dfrA7:sul1, tet(A), catA1, blaCTX-M-15::blaTEM-1B, catB3:blaOXA-1:aac(6’)Ib-cr*	*cnf1, mchF, iha, mchC, vat, iss, pic, iroN, senB, mcm, mchB*	IncY, IncQ1, Col156	IncF[F87*:A-:B10]
E056	NXJD00000000	49	F	Tshwane Academic	ND	+	ST-131	*aac(3)-IIa, catB3, aac(6’)Ib-cr, blaCTX-M-15, blaOXA-1, tet(A)*	*sat, nfaE, iha, iss, gad*	Col(BS512), Col156, ColRNAI	IncF[F4:A-:B52]
E057	NXIS00000000	57	M	Tshwane Academic	Urine	+	ST-665	*tet(A), blaCMY-2*	*Tsh, mchF, iroN, iss, iss*	IncI2, Col(MG828), ColRNAI	IncF[F-:A5:B1], IncI1[ST-12]
E058	NXLI00000000	72	M	Kalafong	ND	+	ST-131	*aac(3)-IIa, catB3, aac(6’)Ib-cr, blaCTX-M-15, blaOXA-1, tet(A)*	*sat, iha, iss, gad*	Col(BS512), ColRNAI	IncI1[Unknown ST]
E060	NXLJ0000000		F	Kalafong	Urine	+	ST-131	*aac(3)-IIa, aadA5, strA, strB, mph(A), sul1, sul2, dfrA17, tet(A), catB3, blaCTX-M-15, blaTEM-1B, blaOXA-1, aac(6’)Ib-cr*	*sat, nfaE, iha, iss, gad*	Col(BS512)	IncF[F2:A1:B-]
E062	NXJE00000000	72	M	Kalafong	Blood	+	ST-131	*tet(A), catB3, aac(6’)Ib-cr, blaCTX-M-15, blaOXA-1*	*sat, iha, iss, gad*	Col(BS512), Col156, ColRNAI	IncI1[Unknown ST]
E063	NXIT00000000	34	F	Kalafong	Urine	+	ST-131	*strA:strB, sul2, dfrA14, blaTEM-1B:blaCTX-M-15, tet(A)*	*sat, iha, iss, gad*	IncX1, IncB/O/K/Z, ColRNAI	IncF[F87*:A4:B1]
K011	NXKS00000000	39	F	Tshwane Academic	Urine	+	ST-410	*ARR-2, strA, aac(3)-IIa, strB, aadA1, mph(A), sul2, sul1, dfrA23, tet(A), catB3, cmlA1, floR, blaOXA-10, blaCTX-M-15, blaOXA-1, aac(6’)Ib-cr*	*lpfA, ccI*	Col(MG828), IncA/C2, IncX4, ColRNAI	IncF[F31:A4:B1]
K075	NXKJ00000000	32	F	Tshwane Academic	Blood	+	ST-648	*strA, strB, aadA2, aph(3’)-IIa, aadA1, fosA, sul3, tet(A), cmlA1*	*air, gad, mchC, mchF, mchF, lpfA, iss, iss, eilA, tsh, iroN, mchB, astA, iha*	Col8282, Col156, ColpVC	IncF[F18:A-:B1], IncHI2[ST-3]
K091	NXKQ00000000	59	M	Tshwane Academic	Urine	+	ST-998	*tet(B), sul1, dfrA1, blaTEM-1B, blaCTX-M-15, aadA1*	*vat, cnf1, senB, iss, gad*	Col8282, Col156	IncF[F1:A1:B23]

*Extended-spectrum β-lactamase.

^†^Multi-locus sequence typing.

^‡^Plasmid MLST (multi-locus sequence typing).

^§^Not detected, missing, or not tested for.

**Male.

^††^ESBL-positive i.e., the isolate is an ESBL producer.

^‡‡^Female.

### Phylogenomic analysis

Whole-genome sequences of *E. coli* isolates curated at the PATRIC website (https://www.patricbrc.org/), between 2013 and 2018, including South African isolates, were downloaded and used alongside this study’s isolates for the whole-genome phylogeny analysis to ensure a current epidemiological and evolutionary analysis (Dataset 1). The phylogeny of the *E. coli* isolates was characterised using Parsnp (https://harvest.readthedocs.io/en/latest/content/parsnp.html)^36^ and edited with Figtree (http://tree.bio.ed.ac.uk/software/figtree/). Isolates of the same clade are highlighted with the same colour whilst those of the same countries have the same label (strain name) colours. The source of the strains viz., animal, environment and human, are shown with distinct colours and annotations. BacWGSTdb was used to type and associate the isolates to international clones, their resistance genes and clinical data^37^. The resistome of strains of close phylogenetic relationship with this study’s isolates were searched for using NCBI’s Pathogen Detection database (https://www.ncbi.nlm.nih.gov/pathogens/isolates#/search/).

## Results

### Patient demographics and isolate characteristics

The 20 isolates were obtained from eight males and 12 females (Table 1) within the ages of 3 and 72, from mainly blood (n = 5) and urine (n = 11). The isolates were all obtained from Kalafong (n = 9) and Steve Biko/Tshwane Academic (n = 11) tertiary academic hospitals, all based in Pretoria, South Africa.

### Antibiotic susceptibility

All the isolates were resistant to the penicillins (amoxicillin and piperacillin), and 3^rd^ and 4^th^ generation cephalosporins, but were susceptible to amoxicillin-clavulanate/sulbactam, piperacillin-tazobactam, cefotaxime-clavulanate, ceftazidime-clavulanate, cephamycin (cefoxitin), and carbapenems. For non-β-lactam antibiotics, almost all isolates were resistant to gentamicin, tobramycin, ciprofloxacin, levofloxacin, nalidixic, tetracycline, and sulphamethoxazole-trimethoprim (SXT), but were susceptible to amikacin and norfloxacin (they were however resistant to norfloxacin according to EUCAST breakpoints) (Supplementary Table S1). Only eight isolates were resistant to minocycline whilst seven were resistant to chloramphenicol and four were resistant to colistin. Two isolates were categorically defined as resistant to tigecycline; however, the MICs (minimum inhibitory concentrations) of the remaining ten isolates (≤1 mg/L) are such that they could be either resistant (>1 mg/L) or intermediate resistant (0.5 mg/L). All but two of the isolates were susceptible to fosfomycin and nitrofurantoin, which are important urinary tract infection (UTI) antibiotics^23,38^. There was categorical agreement between the CLSI and EUCAST MIC breakpoint interpretations for all the isolates and all antibiotics except for norfloxacin (all isolates) and ceftazidime in only E003 (Supplementary Table S1).

### Genomic characteristics

The genomic characteristics of the sequences, in terms of N50, L50, coverage, CRISPR arrays, coding sequences etc. are shown in Supplementary Table S2. The draft genome size of the isolates ranged from 4.8 Mb to 5.5 Mb, with a GC content of 50.2–50.9; except for K091, the coverage of all the isolates were between 90 and 99 (Supplementary Table S2).

### Antimicrobial resistance genes and MGEs

Overall, the *bla*_*CTX-M*_ gene was the most frequently identified in all the *E. coli* isolates. The *bla*_*CTX-M-15*_ gene was identified in 14/20 (70%) isolates. Two other isolates had the *bla*_*CTX-M-14*_ (E040) or the *bla*_*CTX-M-27*_ (E013) gene. The *bla*_*OXA*_ gene was detected in 12 isolates and isolate K11 encoded both *bla*_*OXA-1*_ and one *bla*_*OXA-10*_. The *bla*_*TEM-1B*_ gene was identified in 10 isolates. Nine isolates simultaneously harboured the *bla*_*CTX-M-15*_ and *bla*_*TEM-1B*_ genes. The isolates harboured all three β–lactamase genes. The *bla*_*CMY-2*_ gene was identified in two isolates, E003 and E057 (Table 1), which were however susceptible to cefoxitin. In all these isolates, resistance to penicillins and cephems, except cefoxitin, was observed. However, the isolates became susceptible to the penicillins and cephalosporins in the presence of β-lactamase inhibitors (clavulanate, sulbactam and tazobactam). No carbapenemase genes were found, and carbapenem resistance was absent in all the strains (Table 1 and S1).

We identified only two types of plasmid-mediated quinolone resistance (PMQR) genes in the isolates (Table 1). The *aac(6’*)*Ib-cr* gene was present in 12/20 (60%) isolates, whilst the *OqxAB* gene was present in only one isolate, E035. Q*nr* genes were absent. We identified mutations in the *gyrA, gyrB, parC* and *parE* quinolone resistance-determining region (*QRDR*) genes in all the isolates. *gyrA* had two mutations (A**828**S, D**678**E), *gyrB* had five (E**219**K, A**618**T, R**206**L, E**185**D and S**492**N), *parC* had seven (E**62**K, A**620**V, A**192**V, A**471**G, D**475**E and Q**481**H) and *parE* had three mutations (V**136**I, S**458**A, T**172**A) (Table 2). One isolate (E053) had mutations in all the genes. The most frequent QRDR mutations were I**529**L in *parE*, S**80**1, E**84**V, A**192**V, A**471**G, and Q**841**H in *parC*, S**83**L, D**87**N, and E**678**D in *gyrA*, as well as D**185**E and A**618**T in *gyrB* (Table 2). Sixteen (80%) isolates had mutations in all four QRDR genes. Except for E003, for which no PMQR gene was found, all the strains were resistant to at least three of the fluoroquinolones.

**Table 2:** Mutations in the gyrA, gyrB, parC and parE in the *E. coli* isolates.

Isolate ID	gyrA	gyrB	parC	parE
E003	S83L, D87N, S828A, E678D*	D185E, E219K*	S80I, E475D, E62K*, A620V*	I136V
E005	S83L, D87N, E678D*	D185E, A618T*	S80I, E84V, A192V*, A471G*, Q481H*, E62K*, D475E*	I529L, V136I*
E009	S83L, D87N, E678D, A828S*	D185E, A618T*	S80I, E84V, A192V*, A471G, Q481H*, E62K*, A471G*, D475E*	I529L, V136I*
E011	S83L, D87N, E678D, A828S*	D185E, A618T*	S80I, E84V, E62K*, A192V*, A471G*, D475E*, Q481H*	I529L, V136I*
E013	S83L, D87N, E678D	D185E, A618T*	S80I, E84V, A192V*, A471G*, Q481H*	I529L
E019	S83L, D87N, S828A	D185E	S80I, E475D	I136V, S458A*
E020	S83L, D87N, S828A, D678E*	D185E	S80I, E475D, E62K*	I136V, S458A*
E021	S83L, D87N, E678D, A828S*	D185E, A618T*	S80I, E84V, E62K*, A192V*, A471G*, Q481H*	I529L
E035	D678E*, A828S*	D185E, R206L*	T718A*, E62K*, E475D*	V136I*
E040	—	E185D*	T718A*, E62K*, E475D*	V136I*
E053	D678E*, A828S*	E185D*	E62K*, D475E*	V136I*
E056	S83L, D87N, E678D, A828S*	D185E, A618T*	S80I, E84V, E62K*, A192V*, A471G*, Q481H*, E62K*, D475E*	I529L, V136I*
E057	E678D, S828A,	D185E	E475D*, E62K*	I136V,
E058	S83L, D87N, E678D, A828S*	D185E, A618T*	S80I, E84V, E62K*, A192V*, A471G*, Q481H*, E62K*, D475E*	I529L, V136I*
E060	S83L, D87N, E678D, A828S*	D185E, A618T*	S80I, E84V, E62K*, A192V*, A471G*, D475E*, Q481H*	I529L, V136I*
E062	S83L, D87N, E678D, A828S*	D185E, A618T*	S80I, E84V, E62K* A192V*, A471G*, D475E*, Q481H*	I529L, V136I*
E063	S83L, D87N, E678D, A828*	D185E, A618T*	S80I, E84V, E62K* A192V*, A471G*, D475E*, Q481H*	I529L, V136I*
K011	S83L, D87N, S828A, E678D	D185E	S80I, E475D, E62K*	I136V, S458A
K075	S83L, D678E*, A828S*	D185E, S492N*, A618T*, E656D*	R710C*, E62K*, D475E*	I136V, T172A*
K091	D678E*, A828S*	D185E, R206L*	T718A*, E62K*, D475E*	V136I*

*Putatively novel mutations.

Aminoglycoside resistance genes *aac, aad* and *aph* were also identified. Fourteen (70%) isolates contained *strA* and *strB* genes (Supplementary Table S1). Sixteen (80%) isolates had different *aad* genes; including *aadA5*, *aadA1* and *aadA2* (Tables 1 & S2). Twelve (60%) isolates had *aac* genes: *aac(3*)*-IIa* (n = 8) and *aac(3)-IId* (n = 4). *aph* was found in four isolates: *aph(3’)11a* (3/20, 15%) and *aph(3’)1a* (1/20, 5%) genes. E063 was susceptible to all aminoglycosides, although it contained *strA* and *strB* genes. Furthermore, E035 and K091 both had *aadA*, but were susceptible to tobramycin (Table 1 & S1*)*.

Several isolates contained both *sul* and *dfr* genes, and were co-resistant to trimethoprim and sulfamethoxazole (Table 1 & S1). We identified *sul1* and *sul2* genes in 12/20 (60%) and 13/20 (65%) isolates, respectively. The *sul3* gene, rarely described in the literature, was identified in 2/20 (10%) isolates (E040 and K075). We identified the *dfr* gene in 11/20 (55%) isolates, specifically *dfrA17* (n = 8), *dfrA14* (n = 2) *dfrA1* (n = 1), *dfrA5* (n = 1), *dfrA7* (n = 1), *dfrA12* (n = 1) and *dfrA23* (n = 1). The *sul* and *dfr* genes occurred in diverse combinations in the isolates. In the two isolates with the *sul3* gene, the fosfomycin *fosA* resistance gene was also detected. E003, E056, E057 and E058 were susceptible to SXT, albeit E003 alone had *sul1, sul2* and *dfrA17* genes (Table 1 & S1*)*.

Ten tetracycline-resistant isolates had the *tet*(A) gene, and four isolates had the *tet*(B) gene. Among the two tetracycline-susceptible strains, E011 and E053, E053 harboured a *tet*(A) gene. The chloramphenicol acetylating transferase, *cat*, gene was detected in several isolates: 13/20 (65%) had *catB3* and four had *catA1*. The *cml* and *floR* efflux genes were identified in four and one isolate respectively, albeit only seven isolates expressed chloramphenicol resistance (Table 1 & S1). The *mph(A)* macrolide phosphotransferase gene was found alongside β-lactamases, *sul, cat, str* and aminoglycoside modifying enzyme genes in five isolates, whilst the rifampicin ADP ribosylating transferase *aar*2 gene was only identified in one isolate. In one multidrug-resistant isolate, we detected the chloramphenicol, macrolide and rifampicin resistance genes, together with those conferring resistance to aminoglycosides, fluoroquinolones, β-lactamases, tetracycline and trimethoprim/sulfamethoxazole.

Two isolates had increased colistin MICs, although only E035 had a chromosomal mutation (H**6**R) in the *phoQ* gene, but no mutation was detected in isolate E053. Other mutations were detected in *pmrB* (H**2**R, E**123**D, D**283**G and V**351**I) and *pmrA* (T**31**S, I**128**N and G**144**S) genes (Table 3). No plasmid-mediated colistin gene mutations were detected in either isolate (Table 1). The molecular mechanisms underlying tigecycline resistance in the two isolates remains unknown; no *tet(X)* resistance genes were found in the genomes^31,39^. The resistome of all the isolates are found in Dataset 2.

**Table 3:** Colistin MICs and mutations in pmrB, pmrA, phoP, phoQ and mgrB in the *E. coli* isolates.

Isolate ID	MIC (mg/L)	*pmr*B	*pmr*A	*pho*P	*pho*Q	*mgr*B
E035	>4	—	Del RRHN (113–116), T31S*, I28N*, G144S*	—	H6R	—
E053	>4	H2R*, E123D*, D283G*, V351I*	Del RRHN (113–116), T31S*, I128N*, G144S*	—	—	—
K075	4	D123E, I351V, A360V	Del RRHN (113–116), S31T, N124I, S140G	—	H6R, L467M	A8V
K091	4	—	Del RRHN (113–116)	—	H6R	—

*Putatively novel mutations.

The most common plasmid replicon found in the genomes was IncF (n = 17, 85%). The isolates also contained the IncI (4/20, 20%), IncN (1/20, 5%) and IncH (3/20, 15%) replicons. Five (25%) isolates had more than one plasmid replicon (Table 1). The E035 isolate had the IncH plasmid with an ln369 integron (Table 4).

**Table 4:** Class 1 integrons, gene cassettes and sequence types found in the *Escherichia coli* isolates.

Sample code	MLST	pMLST	Integron	Cassette arrays					
				GC1	GC2	GC3	GC4	GC5	GC6
E013	ST131	IncF	—	—	—	—	—	—	—
E021	ST131	IncF	ln54	*dfrA17*	*aadA5*	—	—	—	—
E040	ST95	IncF	ln13/641	*dfrA5*	*psp*	*aadA2*	*cmlA1a*	*aadA1*	*qacH2*
E053	ST73	IncF	ln22	*dfrA7*	—	—	—	—	—
E057	ST665	IncF	—	—	—	—	—	—	—
E063	ST131	IncF	—	—	—	—	—	—	—
E003	ST744	IncF	ln54	*dfrA17*	*aadA5*	—	—	—	—
E020	ST617	IncF	ln54	*dfrA17*	*aadA5*	—	—	—	—
E035	ST10	IncH	ln369	*dfrA1b*	*aadA1b*	—	—	—	—
E056	ST131	IncF	—	—	—	—	—	—	—
E062	ST131	IncF	—	—	—	—	—	—	—
K075	ST648	IncF	—	*estX3*	*psp*	*aadA2*	*cmlA1a*	*aadA1*	*qacH2*
K091	ST998	IncI	ln369	*dfrA1b*	*aadA1b*	—	—	—	—
E011	ST131	IncF	ln54	*dfrA17*	*aadA5*	—	—	—	—
K011	ST410	—	ln467	*arr2*	*cmlA1g*	*blaoxa*—*10, aadA1e*	—	—	—
E005	ST131	IncI	ln54	*dfrA17*	*aadA5*	—	—	—	—
E019	ST617	IncF	ln54	*dfrA17*	*aadA5*	—	—	—	—
E009	ST131	IncF	ln54	*dfrA17*	*aadA5*	—	—	—	—
E058	ST131	IncF	—	—	—	—	—	—	
E060	ST131	IncF	ln54	*dfrA17*	*aadA5*	—	—	—	—

Notably, *dfrA, aadA* and *QacEΔ1* genes were almost always found as gene cassettes within the class 1 integrons; all the isolates contained only class 1 integrons, which were associated with a variety of cassette arrays (Table 4). The most frequently identified cassettes were *dfrA17* and *aadA5*, which were always associated with integron ln54 and MLST ST131. Cassette arrays *dfrA5-psp-aadA2-cmlA1a-aadA1-qac* and *estX3-psp-aadA2-cmlA1a-aadA1a-qac* were detected in isolates i.e., E040 and K075: *fosA* and *sul3* genes were identified in both these MDR isolates. Other integron types identified were ln369, ln22, ln13, ln54, ln467, ln641 and ln369. Isolate E040 had two integron types: ln13 and ln641.

As shown in Table 5, most of the resistance genes were bracketed by either class 1 integrons, ISs and transposons or by all three. Composite and Tn3 transposons as well as IS6 insertion sequences were most common, with IS*Ec9* being commonly found with *bla*_CTX-M_ genes. The resistomes and mobilomes in these isolates were found to have between 98% and 100% sequence identity and length coverage with already deposited genome sequences at Genbank; the most common among these were *E. xiangfangensis* WCHEX045001 chromosome (CP043382.1) and *E. coli* GZ04-0086 plasmid pCTXM-GZ04 (CP042337.1) (Table 5).

**Table 5:** MGEs associated with antibiotic resistance genes in the *E. coli* strains.

Strain (MLST)	Contig	Synteny of resistance genes and MGEs	Plasmid/chromosomal sequence with closest nucleotide homology (accession number)
E003 (ST744)	36	*sugE::bla*_*CMY-2*_*:IS*1380 *(ISEc9)*	*Salmonella* Derby strain 116 plasmid (MK191846.1)
	73	IS*1*:*catA1::Tn3(TnAs3)*:::*IntI1:dfrA17:ant(3”)-Ia:QacEΔ1:sul1*	*E. coli* 1223 chromosome (CP023383.1)
	99	*aph(*6*)-Id:aph(3”)-Ib:sul2*	*E. coli* O111:H- 110512 plasmid pO111-110512_1 (AP019762.1)
	110	*tet*(B):*tetR*:*ArsR:IS1*	*E. coli* strain GZ04-0086 plasmid pCTXM-GZ04 (CP042337.1)
E005 (ST131)	50	*bla*_TEM-1B_:*IS1*	*S. sonnei* 183660 plasmid p183660 (KX008967.1)
	51	*Sul2:aph(3”)-Ib:aph(*6*)-Id:relaxase:tetR:tet*(A):::*Tn*3	*E. coli* Es_ST410_NW1_NDM_09_2017 plasmid pEsST410_NW_3 (CP031233.1)
	52	*QacEΔ1:sul1::::resolvase:IS6 (IS6100)::tetR::mph(A)*	*E. coli* CVM N56639 plasmid pN56639 (CP043753.1)
	67	IS1380(ISEc9):*bla*_CTX-M-15_	*E. coli* 1500 plasmid pEc1500_CTX (CP040270.1)
	70	*aadA5:dfrA17:IntI1:IS6-like IS26*	*E. coli* 131 plasmid p146-1 (CP041573.1)
	74	*IS6-like IS26:catB3:bla*_*OXA-1*_*:aac(6’)-Ib-cr5:IS6-like IS26*	*E. xiangfangensis* WCHEX045001 chromosome (CP043382.1); *E. coli* GZ04-0086 plasmid pCTXM-GZ04 (CP042337.1)
E009 (ST131)	51	*bla*_TEM-1B_:IS*1*	*Shigella sonnei* 183660 plasmid p183660 (KX008967.1)
	52	*IS6 IS15DIV::::Sul2:aph(3”)-Ib:aph(6)-Id::relaxase:tetR:tet*(A)*:::Tn3*	*E. coli* Es_ST410_NW1_NDM_09_2017 plasmid pEsST410_NW_3 (CP031233.1)
	57	*QacEΔ1:sul1:::::resolvase:IS6::tetR*	*E. coli* CVM N56639 plasmid pN56639 (CP043753.1)
	68	IS1380(IS*Ec9)*:*bla*_CTX-M-15_	*E. coli* 1500 plasmid pEc1500_CTX (CP040270.1)
	70	*aadA5:dfrA17:IntI1:IS*6*-like IS2*6	*E. coli* 131 plasmid p146-1 (CP041573.1)
	74	*IS*6*-like IS*26:*catB3:bla*_*OXA-1*_*:aac(6’)-Ib-cr5: IS6-like IS*26	*E. xiangfangensis* WCHEX045001 chromosome (CP043382.1); *E. coli* GZ04-0086 plasmid pCTXM-GZ04 (CP042337.1)
	79	*aac(3)-IId::IS4*	*E. coli* AR_0086 plasmid unnamed1 (CP032202.1)
E011 (ST131)	46	IS*91:aph(*6*)-Id:aph(3’):dfrA14:aph(3”)-Ib:sul2*	*E. coli* Ec20-Lar plasmid unnamed (MK396099.1)
	63	*IntI1:dfrA17:aadA5:QacEΔ1:::::resolvase:*IS6*::tetR::mph*(A):IS*6*	*E. coli* Ecol_AZ146 plasmid pECAZ146_1 (CP018990.1)
	88	IS*6*:Tn*3*::*bla*_CTX-M-15_::IS*1380*	*E. coli* Ecol_AZ146 chromosome (CP018991.1); *E. coli* 219 plasmid unnamed (CP020515.1)
	96	*aac(3)-IIa*::IS3:IS6	*E. xiangfangensis* WCHEX045001 chromosome (CP043382.1)
	103	IS6*:catB3:bla*_*OXA-1*_*:aac(6’)-Ib-cr5:IS6*	
E013 (ST131)	60	IS*6*IS*15DI:tet(A):tetR::aph(6)-Id:aph(3”)-Ib:sul2:*IS*6*(IS*15DIV)*	*E. coli* strain H105 plasmid pH105 (CP021871.1)
	79	IS*6-*like (IS*26):bla*_*CTX-M-15/27*_*:IS6-like* (*IS26*)	*E. coli* strain 131 plasmid p146-1 (CP041573.1)
E019 (ST617)	64	*IntI1:dfrA17:AadA5:QacEΔ1:sul1:::::resolvase:IS6::tetR::mph*(A)::IS*6*-like(IS*26*)	*E. coli* VRES-hospital6495150 plasmid: 1 (LR595886.1)
	74	IS91*::::::sul2:aph(3”)-Ib*	*S*. Manhattan SA20084699 plasmid unnamed2 (CP022499.1)
	76	Tn*3::bla*_*CTX-M-*1*5*_*:IS1380 (ISEc9)*	*K. pneumoniae* FDAARGOS_447 plasmid unnamed3 (CP023950.1)
	77	IS*1::::tetR:tet*(B):*tet*(C):*:*IS*4 (ISVsa5)*	*S. flexneri* FDAARGOS_535 chromosome (CP034060.1)
	79	*RepA:*IS*6(*IS*15DIV)*:Tn*3::catA1:IS1*	*E. coli* 675SK2 plasmid p675SK2_B (CP027703.1)
	87	IS6-like IS*26*:::::*aac(3)-IId::IS4*	*K. pneumoniae* PIMB15ND2KP27 plasmid pKP27-NDM4 (CP041642.1)
	97	IS*6-*like IS*26:aac(6’)-Ib-cr5:bla*_*OXA-1*_*:catB3:IS6-like (IS26)*	*E. xiangfangensis* WCHEX045001 chromosome (CP043382.1); *E. coli* GZ04-0086 plasmid pCTXM-GZ04 (CP042337.1)
E020 (ST617)	69	IS*6*::*IntI1(In54):dfrA17:aadA5:QacEΔ1:Sul1::::::IS6:tetR:tet:mph(A):*IS*6*	*Shigella flexneri* ID134382 plasmid pSf1 (MG767300.1)
	82	*Sul2:aph(3”)-Ib:aph(6)-Id:*IS*91*	*E. coli* WCHEC005237 plasmid p1_005237 (CP026572.2)
	83	*bla*_TEM-1B_:IS*1*	*E. coli* plasmid pI1-34TF (LN850163.1)
	84	IS6 (IS*15DIV)*:Tn3::*bla*_CTX-M-15_:IS1380 (ISEc9)::IS6	*K. pneumoniae* FDAARGOS_447 plasmid unnamed3 (CP023950.1)
	88	IS1::::*tetR*:*tet*(B):*tet*(C)*:*IS4	*S. enterica Wien ZM3 plasmid pZM3* (MK797990.1)
	89	IS6 (IS*15DIV*)::Tn3(Tn*As3)*:*catA1*	*E. coli* 6*75SK2 plasmid p*6*75SK2_B* (CP027703.1)
	105	IS*6:catB*3*:bla*_OXA-1_:*aac(*6*’)-Ib-cr5:*IS*6*	*E. coli* strain GZ04-0086 plasmid pCTXM-GZ04 (CP042337.1)
E021 (ST131)	56	IS6-like (IS26)::*mph*(A)::*tetR::: IS6-like IS6100::::::sul1:QacEΔ1:aadA5:dfrA17:IntI1(In54)*	*E. coli* 131 plasmid p2629-1 (CP041542.1)
	62	IS*6*(IS*15DIV)*:::*sul2:aph(3”)-Ib:aph(6)-Id::tetR:tet*(A)*::::IS6* (*IS15DI)*	*E. coli* strain 4/4 plasmid p4_4.1 (CP023827.1)
	82	IS1380-like (ISEc9):*bla*_CTX-M-15_::Tn*3*-like Tn3 family	*E. coli* CFSAN061761 chromosome (CP042903.1); plasmid: *K. pneumoniae* p14ARS_VSM0843-1(LR697132.1)
	84	IS*6*-like(IS26):IS3 family::*aac(3)-IIa:*IS*6-*like IS26	*E. xiangfangensis* WCHEX045001 chromosome (CP043382.1); *E. coli* Ec-050 plasmid pEc-050-NDM-5 (CP043230.1)
	90	IS6-like IS26:*catB3:bla*_*OXA-1*_:*aac(6’)-Ib-cr5*:IS6-like IS26	*E. xiangfangensis* WCHEX045001 chromosome (CP043382.1)
E035 (ST10)	36	*IS3:IS21(IS1326):::sul1:QacEΔ1:AadA1:dfrA1:IntI1*	*E. coli* O16:H48 strain PG20180175 plasmid pPG20180175.1-IncAC2 (CP043190.1)
	41	*bla*_TEM-1B_:recombinase::IS1380(IS*Ec9)*:*bla*_CTX-M-15_::Tn*3:*IS1	*S. Typhi* WGS1146 plasmid unnamed (CP040574.1)
	130	*ArsR*:*tetR:tet*(B):*:*Is1	*E. coli* strain CFSAN061761 chromosome (CP042903.1)
E040 (ST95)	55	IS256:QacL:aadA1:CmlA1:AadA2:::*IntI1*:::Tn3-like Tn*As1*	*E. coli* CFSAN061772 plasmid pCFSAN061772_02 (CP042895.1)
	83	IS26 (*tnpA*26):*sul3:::mefB-*IS26*:tnpA*26	*E. coli* F2_14D plasmid pF2_14D_HI2 (MK461931.1)
	84	IS*26*:*repA*-IS*26*:*repC:sul2:strA*	*E. coli* 2009-52 plasmid pSDJ2009-52F (MH195200.1)
	86	IS4:*aph(3’)-IIa*	*E. coli* NCYU-25-82 plasmid pNCYU-25-82-7 (CP042634.1)
	89	IS91:*bla*_TEM-1B_	*E. coli* O111:H- 110512 plasmid pO111-110512_1 DNA (AP019762.1)
	91	*IS6-like IS26::fosA3*:IS5/IS1182	*E. coli* AR Bank #0349 plasmid pAR349 (CP041997.1)
	99	*aph(6)-Id:aph(*3*”)-Ib*	*E. coli* CVM N16EC0140 plasmid pN16EC0140-1 (CP043748.1)
	107	IS5/IS1182:*bla*_CTX-M-14_::IS6-like IS26	*E. coli* AR Bank #0349 plasmid pAR349 (CP041997.1)
	119	*IntI1:dfrA5:IntI1-IS26*	*E. coli* 2009-52 plasmid pSDJ2009-52F ()
E053 (ST73)	45	Tn*3*::*tetR:tet*(A)::::::::::::IS110::::IS21	*E. coli* PU-1 chromosome (CP042246.1)
	60	IS6:Tn3::*bla*_CTX-M-15_:IS1380 (IS*Ec9)*	*E. cloacae* NH77 chromosome (CP040827.1)
	77	*aph(6)-Id:aph(*3*”)-Ib:sul2*	*E. coli* FDAARGOS_772 chromosome (CP041002.1)
	80	Tn3-like (TnAs3):::IntI1:*dfrA7:QacEΔ1::IS6* (*IS15DIV)*	*E. coli* O104:H4 FWSEC. 0009 chromosome (CP031902.1)
	93	IS6::aac(3)-IIa::IS*3*	*E. xiangfangensis* WCHEX045001 chromosome (CP043382.1)
	103	IS6-like IS26:*catB*3*:bla*_*OXA-1*_:*aac(6’)-Ib-cr5:IS6-like IS26*	
	110	Tn3:*catA1*	*S. enterica Wien ZM3 plasmid pZM3* (MK797990.1)
	137	IS6:*aph(3’)-Ia*	*K. pneumoniae* WCHKP7E2 plasmid pCMY2_085072 (CP028804.2)
E056 (ST131)	53	*tetR:tet*(A)::*Tn1721:resolvase:Tn5403:Tn5403:::Tn2 tnpA*	*E. coli* strain 661 (LT985271.1)
	84	IS1380-like IS*Ec9*:*bla*_CTX-M-15_::Tn*3*-like Tn3	*E. coli* strain CFSAN061761 chromosome (CP042903.1)
	97	*IS6-like IS26:IS3::aac(3)-IIa:IS6-like IS26*	*E. coli* GZ04-0086 plasmid pCTXM-GZ04 (CP042337.1)
	102	IS6-like IS26*:catB3*:*bla*_0XA-1_:*aac(6’)-Ib-cr5: IS6-like IS26*	
E057 (ST665)	18	IS1380 IS*Ec9:bla*_CMY-2_::*sugE*	*Salmonella* Derby strain 116 plasmid (MK191846.1)
	72	*Tn*3:::*tet*(A):*tetR:*relaxase	*E. coli* plasmid pHN32wt (MH450052.1)
E058 (ST131)	42	*Tn*3*:::Tn*3*:resolvase:Tn*3*:::tet(A):tetR*	*E. coli* 661 plasmid: RCS59_p (LT985271.1)
	51	*Tn3::bla*_*CTX-M-15*_*:IS*1380 *ISEc9*	*K. pneumoniae* FDAARGOS_447 plasmid unnamed3 (CP023950.1)
	61	IS3*:aac(*3*)-IIa*	*E. coli AR21*6*.2b* plasmid pMPNDM-5 (CP043944.1)
	66	*IS6-like IS*26*:catB3:bla*_*OXA-1*_*:aac(6’)-Ib-cr5:IS6-like IS26*	*E. xiangfangensis* WCHEX045001 chromosome (CP043382.1); *E. coli* GZ04-0086 plasmid pCTXM-GZ04 (CP042337.1)
E060 (ST131)	14	Tn*3:resolvase*:Tn3:::*tet*(A):*tetR*	*E. coli* 4/0 chromosome (CP023849.1)
	26	*bla*_TEM-1B_:recombinase:Tn3	*E. coli* 131 plasmid p2448-1 (CP041547.1)
	39	IS1380 ISEc9:*bla*_CTX-M-15_::Tn*3*	*E. coli* 131 chromosome (CP041581.1)
	43	*IntI1*:*dfrA17:aadA5:QacEΔ1:sul1:::::resolvase:*IS*6::tetR::mph*(A)	*E. coli* VRES-hospital6495150 plasmid: 1 (LR595886.1)
	52	IS91*::::::sul2:aph(3”)-Ib:*	*S*. Manhattan SA20084699 plasmid unnamed2 (CP022499.1)
	66	IS3*:aac(3)-IIa*	*E. coli* AR216.2b plasmid pMPNDM-5 (Sequence ID: CP043944.1)
	70	*catB3:bla*_*OXA-1*_*:aac(6’)-Ib-cr5:resolvase*	*E. xiangfangensis* WCHEX045001 chromosome (CP043382.1); *E. coli* GZ04-0086 plasmid pCTXM-GZ04 (CP042337.1)
E062 (ST131)	52	*tetR:tet*(A):::Tn*3* family::Tn*3* family:::Tn*3* family	*E. coli* 661 plasmid: RCS59_p (LT985271.1)
	70	IS*1380* family IS*Ec9*:*bla*_CTX-M-15_::Tn*3* family	*K. pneumoniae* FDAARGOS_447 plasmid unnamed3 (CP023950.1)
	83	IS*6*-like IS*26*:*catB3:bla*_*OXA-1*_:*aac(6’)-Ib-cr5:IS6-like IS26*	*E. xiangfangensis* WCHEX045001 chromosome (CP043382.1); *K. pneumoniae* 18-2374 plasmid pSECR18-2374A (CP041928.1)
E063 (ST131)	25	IS91:*aph(6)-Id:aph(3’)-Ib:dfrA14:aph(3”)-Ib:sul2*::::::::: *bla*_*TEM-1*_:::IS1380 ISEc9:*bla*_CTX-M-15_::Tn3 family	*E. coli* Ecol_AZ161 plasmid pECAZ161_1 (CP019011.1)
	72	Tn3 family:::*tet*(A):*tetR:relaxase:Tn3 family*	*E. coli* Ec-050 plasmid pEc-050-NDM-5 (CP043230.1)
K011 *(ST410)*	19	*aph(6)-Id:aph(3”)-Ib:sul2*	*K. pneumoniae* PIMB15ND2KP27 plasmid pKP27-MCR1 (CP041641.1)
	52	*IntI1:Arr-2:CmlA5:bla*_*OXA-10*_*:aadA1:QacEΔ1:sul1:IS91:::dfrA23:::: IS110:Tn3:resolvase::IntI1:repA*	*E. coli* C600_pConj125k plasmid pConj125k (MK033499.1)
	71	*Resolvase*::IS*91:floR:lysR*	*E. coli* O16:H48 PG20180173 plasmid pPG20180173.1-IncAC2 (CP043192.1)
	76	*IS6::tetR::mph*(A)	*K. pneumoniae* 555 plasmid pSCKLB555-4 (CP043936.1)
	78	IS1380:*bla*_CTX-M-15_::*Tn*3	*E. coli* 219 plasmid unnamed (CP020515.1)
	80	*Tn3:::tet(A):tetR:relaxase*	*E. coli* CVM N16EC0879 plasmid pN16EC0879-1 (CP043745.1)
	84	IS*3::aac(3)-IIa*	*E. coli* strain AR216.2b plasmid pMPNDM-5 (CP043944.1)
	86	*catB3:bla*_*OXA-1*_*:aac(6’)-Ib-cr5:resolvase*	*E. xiangfangensis* WCHEX045001 chromosome (CP043382.1);
K075 (ST648)	78	*IS6:IntI1::aadA2:CmlA1:aadA1:QacL:IS256:IS6*	*S*. Typhimurium sg_wt7 chromosome/plasmid (CP036168.1)
	83	IS5/IS1133-like IS903B:IS*3*:*aph (3”)-Ib:aph(6)-Id*	
	94	*aph(3’)-IIa:*IS4::IS91 family	*E. coli* 13P477T plasmid p13P477T-7 (CP021103.1)
	96	Tn3-like Tn*As1::tet*(A):*tetR::Tn3 family*	*E. coli* CVM N16EC0879 plasmid pN16EC0879-1 (CP043745.1)
	111	IS*2*6*:sul3::mefB-IS26*	*E. coli* F2_14D plasmid pF2_14D_HI2 (MK461931.1)
	122	IS5/IS1182:*fosA3*::IS*6*	*E. coli* AR Bank #0349 plasmid pAR349 (CP041997.1)
	143	IS5/IS1182:*bla*_CTX-M-14_::IS6-like IS26	*E. coli* 1106 plasmid p1106-IncHI2 (MG825373.1); *E. coli* AR Bank #0349 plasmid pAR349 (CP041997.1)
K091 (ST998)	37	IS6*:IntI1:dfrA1:aadA1:QacEΔ1:sul1::::IS21 IS1326:IS3:::*IS*6*	*E. coli* O16:H48 PG20180173 plasmid pPG20180173.1-IncAC2 (CP043192.1)
	41	*bla*_*TEM-1*_*:recombinase::IS1380 ISEc9:bla*_*CTX-M-15*_*::Tn3:IS1*	*E. coli* ECONIH6 plasmid pECO-6dfa (CP026200.1)
	54	*ArsR:tetR:tet*(B):*:IS1*	*E. coli* CFSAN061761 chromosome (CP042903.1)

### Sequence types and phylogenomics

**Figure 1:**
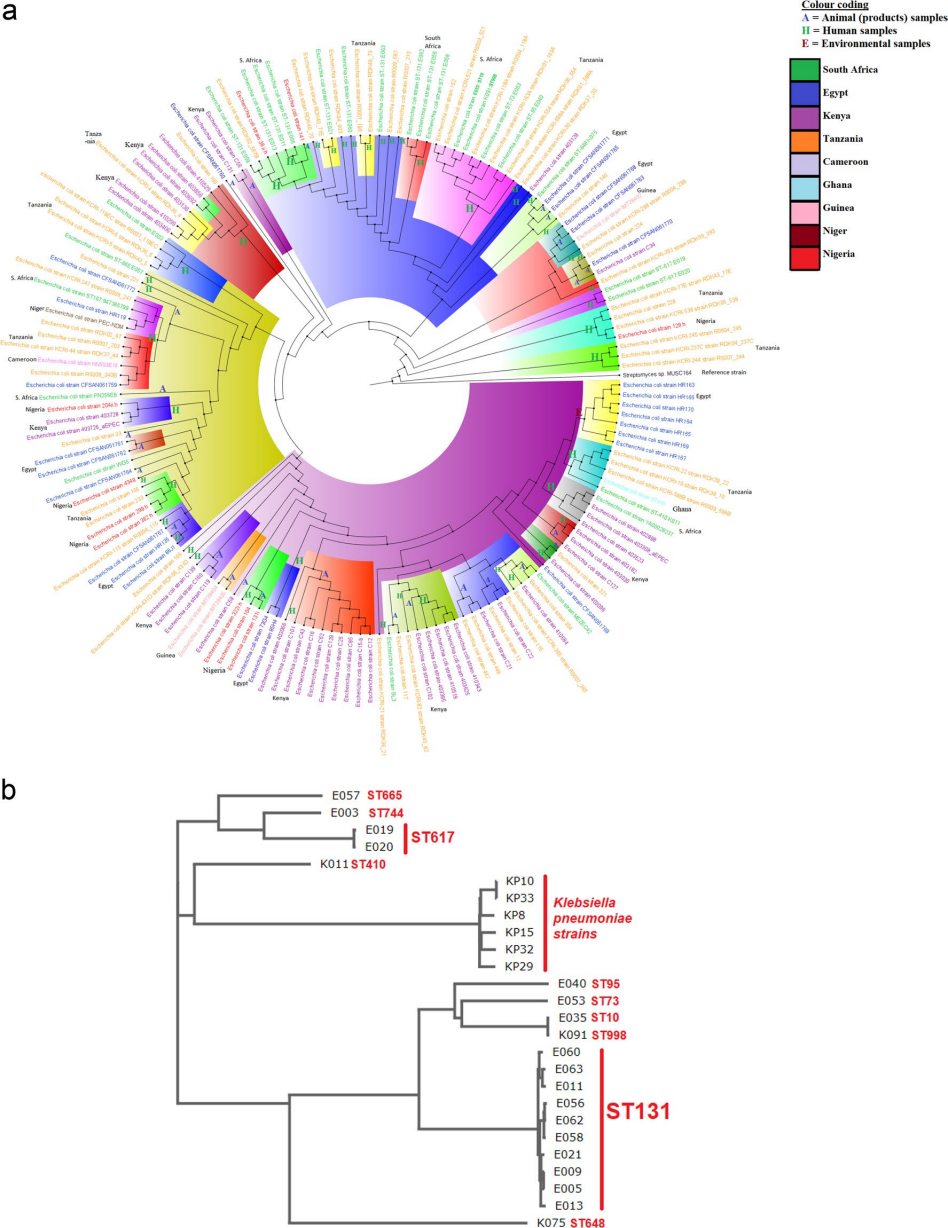
A neighbour-joining phylogenetic tree of African *E. coli* isolates. (**A**) The annotations show that the S. African strains used in this study were basically related to strains from Tanzania and Egypt. Strains of same and different clones clustered together in many instances, with isolates of the same clone only clustering together in a few instances. (**B**) The strains clustered according to sequence types, with ST131 and ST617 strains being on the same branches. However, ST10 and ST998 were also found on the same branch, showing the higher resolution of whole-genome MLST over conventional MLST.

**Figure 2:**
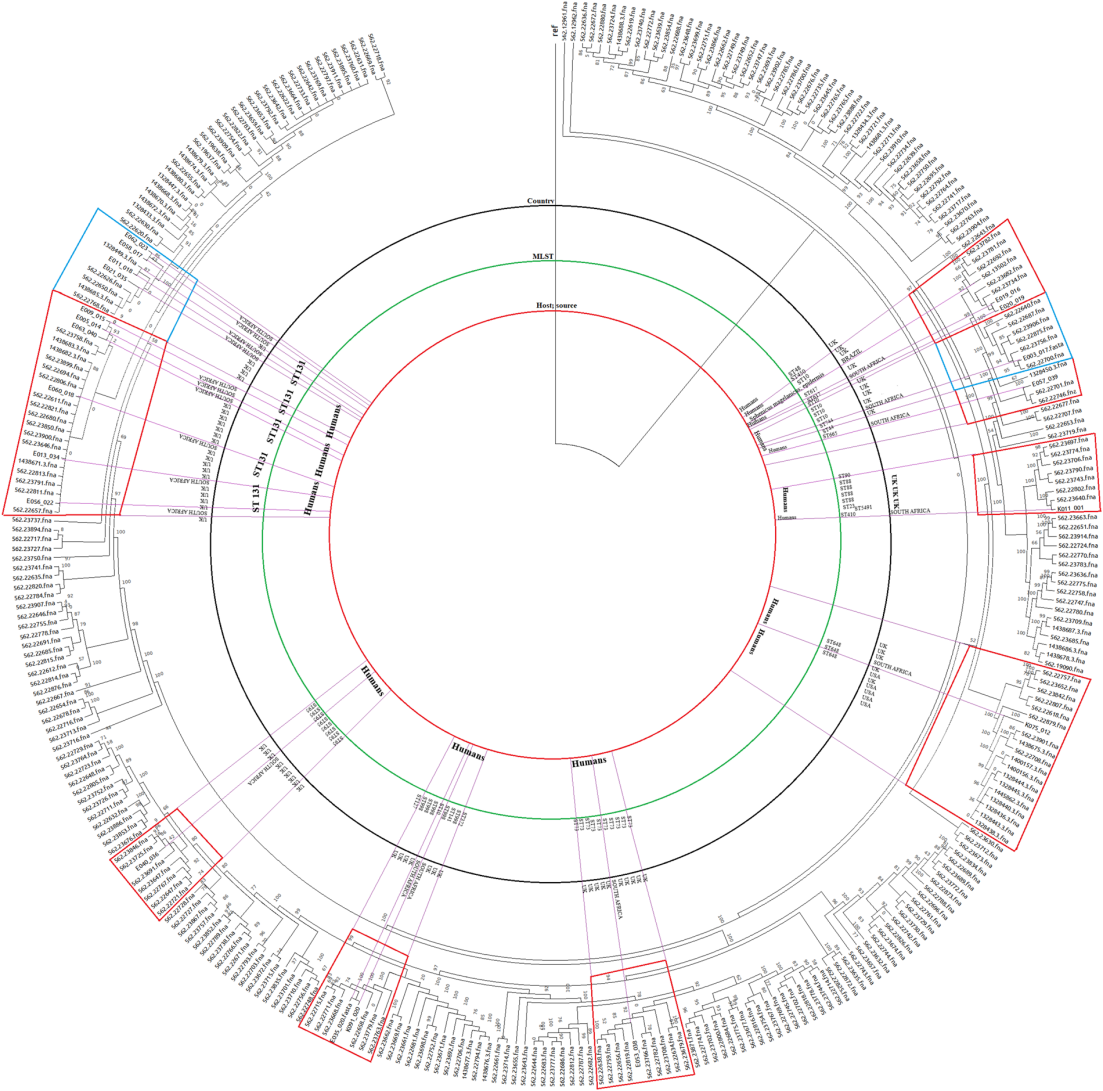
A neighbour-joining phylogenetic tree of global *E. coli* isolates depicting lineages between the Pretoria (South Africa) sequences in red clusters, Durban (South Africa) sequences in blue clusters and international sequences. The S. African strains were mainly related to strains from the UK.

The *E. coli* isolates were multiclonal, with ST131 (n = 10), ST617 (n = 2) and singletons of ST10, ST73, ST95, ST410, ST648, ST665, ST744 and ST998 being identified (Tables 1, 4 & 5). The two ST617 strains (E019 and E020) virtually have the same plasmid replicon types, resistome, virulome, integron types, genomic features and patient characteristics (66-year old female from Tshwane Academic hospital). A slightly similar observation was also made for isolates E005 and E009 (ST131). However, these patterns were not observed among the other strains of ST131 that had different patient demographics (different sexes, ages and hospitals) (Figs 1, 2)

The phylogenomic analyses of the isolates showed that they were more closely aligned to strains from Tanzania and Egypt than to any other country; none of the isolates were phylogenetically related to any strain from South Africa. Specifically, E019 and E0120 (ST617), described above to have the same resistome, mobilome, virulome, genomic and demographic features, as well as K091 (ST998) and E035 (ST10), were found to be on the same clade/node whilst E005 and E009, found to also have very similar genomic characteristics, were distantly placed on different branches on the tree (Figs 1, 2; Dataset 1). E062, E056 and E058, as well as E005 and E011, all ST131, clustered together on one branch and clade. However, ST131 strains such as E063 and E060 were distant from other ST131 strains; other STs such as E003 (ST744), E057 (ST665) and K011 (ST410) were not closely related to any strains on the tree (Fig. 1A; Supplementary Dataset 1).

K075 was on the same clonal node as CFSAN061771 (ST1485) from Egypt and same clade/branch as CFSAN061765 (ST1722:*bla*_CMY-2_, *bla*_EC_, *bla*_OXA-244_), also from Egypt. E040 and RDK06_554 (Tanzania: *aph(*3*”)-Ib, aph(6)-Id, bla*_EC_*, bla*_TEM-1_, *dfrA5, sul2*), E053 and R0004_118A (Tanzania ST73: *aph(3”)-Ib, aph(6)-Id, bla*_EC-5_, *bla*_TEM-1_, *dfrA7, qacEΔ1, sul1, sul2*), E021 and RDK40_71E (Tanzania, ST131: *aac(3)-IIa, aac(6’)-Ib-cr5, aadA5, aph(3”)-Ib, aph(6)-Id, bla*_CTX-M-15_, *bla*_EC_, *bla*_OXA-1_, *catB3, dfrA17, mph(A), qacEdelta1, sul1, sul2*, *tet*(A)), as well as E009 and RDK02_567B (Tanzania ST10: *aadA5, bla*_CTX-M_*, bla*_EC_*, dfrA17, mph(A), qacEΔ1, sul1*), were all closely related on the same clonal branches. Globally, the strains were closely related to strains from mainly the UK (Fig. 2), including *E. coli* 05:H4 strain ECO0291 (with E057) and *E. coli* 021:H52 strain ECO0336 (with E003), all of phylogroup A. Evidently, the STs and resistance genes between this study’s isolates and those from Egypt and Tanzania were different.

### Virulome

**Figure 3:**
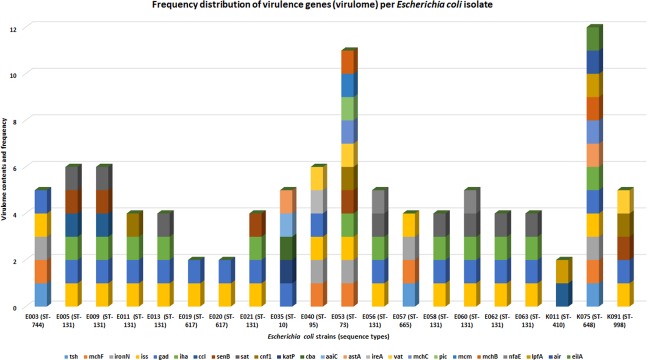
Frequency distribution of virulence (virulome) genes found per *Escherichia coli* isolate. Several virulence genes were found in the isolates, ranging from two to 24. Some isolates had more virulence genes diversity than others, with some virulence genes being found in only an isolate from blood (K075).

**Figure 4:**
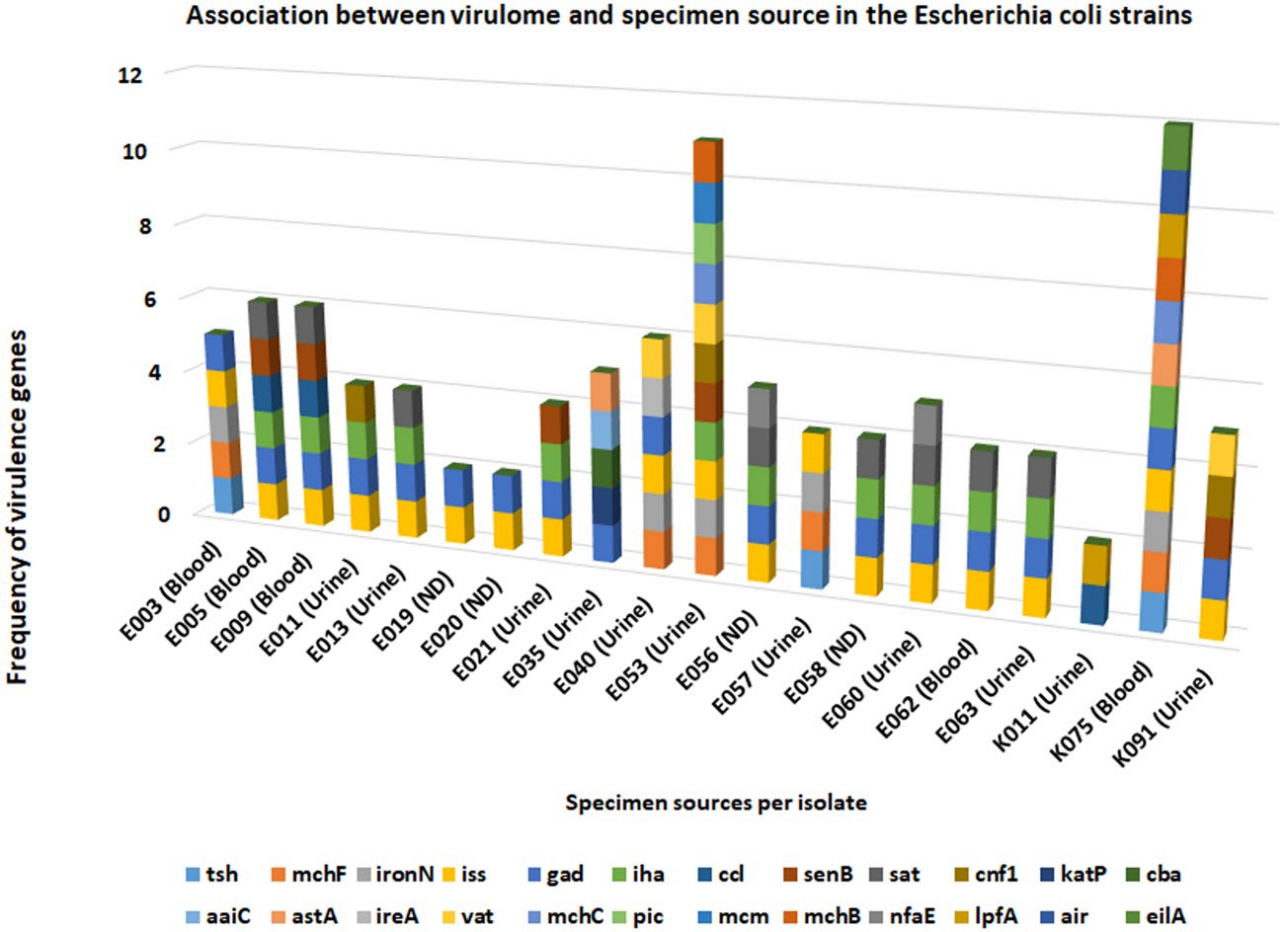
Association between the virulome and specimen source of each *Escherichia coli* isolate. The isolate with the highest virulome composition and diversity was from blood (K075) followed by one from urine (E053). Thus, there is little to suggest that isolates from blood had more virulence genes than those from urine as shown in the chart.

A total of 24 virulence genes (Table 1) were recorded in all the isolates combined, with E053 (n = 11 virulence genes) and K075 (n = 12 virulence genes) having the most repertoire of virulence genes; K011, E019 and E020 had the least (n = 2 virulence genes). Virulome similarity could be seen between isolates belonging to the same clone than between those of different clones. E035 (ST10) and E040 (ST95) had unique set of virulence genes, whilst K075 and E053 had very diverse set of virulence genes. The commonest virulence genes among the strains were *iss* (n = 18 isolates)*, gad* (n = 17 isolates) and *iha* (n = 12 isolates), with *katP, cba, aaiC, ireA, pic, mcm, air*, and *eilA* occurring in single isolates (Fig. 3; Supplementary File 2). A specimen source-virulome association comparison was made (Fig. 4; Supplementary file 2) and there was little evidence to suggest that strains from blood had more virulence genes than those from urine, albeit the strain with the most virulence genes was from blood. *eilA, air*, and *lpfA* were only found in a single strain (K075) from blood.

## Discussion

In this study, 20 clinical *E. coli* isolates showed an extensive repertoire of resistance genes bracketed by composite Tn*3* transposons, ISs and class 1 integrons on contigs containing mainly IncF plasmid replicons in multiclonal and same clone strains. The strains can be rightly defined as MDR strains due to their phenotypic resistance to penicillins, cephalosporins, aztreonam, fluoroquinolones, aminoglycoside, tetracycline and SXT. The ESBL phenotypes of the strains, as confirmed by the disc synergy test, was confirmed by the presence of ESBL genes (*bla*_CTX-M_, *bla*_OXA_, and *bla*_TEM-1B_) and the susceptibility of the strains to the β-lactamase inhibitors viz., clavulanic acid, sulbactam and tazobactam, when combined with either the penicillins or cephalosporins. A major observation was the cefoxitin susceptibility of E003 and E057, both of which harboured *bla*_CMY-2_ within the same genetic context of IS1380 IS*Ec9:bla*_CMY-2_::*sugE* (the reverse orientation was found in E003) that was of closest nucleotide identity with *Salmonella* Derby strain 116 plasmid (MK191846.1). Whereas the genetic context of these *bla*_CMY-2_ suggests that they might have been acquired horizontally, the host strains could not show phenotypic resistance to cefoxitin as expected of strains with acquired *bla*_CMY-2_^40,41^.

Moreover, AmpC β-lactamases such as *bla*_CMY-2_ are not expected to be inhibited by β-lactamase inhibitors (clavulanate, sulbactam and tazobactam) as was observed in E003 and E057, which were susceptible to β-lactam/β-lactamase inhibitor combinations. These observations strongly suggest that the *bla*_CMY-2_ genes in these two isolates might not have been expressed or were not active in the isolates^40,41^. Besides these two isolates, other resistance discrepancies were observed between the phenomes and genomes of other isolates that harboured resistance genes but did not express resistance phenotypically. Examples include the susceptibility of E063, E035 and K091 strains to tobramycin although they had *aadA, aph(3’)-IIa, or strA/B* genes. Other such discrepancies, already stated in the results above, can be found by studying Supplementary Tables 1 and 2.

The *bla*_*CTX-M-15*_ gene, surrounded by composite transposons mostly including IS*Ec9*, was found in almost all the *E. coli* isolates, which is higher than that reported by two other studies from South Africa where 45% and 59% of isolates in Port Elizabeth and Cape Town, respectively, possessed this gene. Furthermore, the co-existence of *bla*_*OXA-10*_ and *bla*_*TEM*_ genes is consistent with observations in local studies^17,42^ as well as in studies from China^42,43^. Similarly, the co-occurrence of *aac(6’)Ib-cr* and *bla*_*CTX-M-15*_ are consistent with observations made in isolates from China and the USA^44,45^. IS*Ec9* and IncF plasmids have been implicated in the mobilization and dissemination of *bla*_*CTX-M-15*_ globally^20,21,45,46^; the IncF plasmids mobilizing *bla*_*CTX-M-15*_ also co-harboured *aac(6’)Ib-cr, bla*_*OXA-10*_ and *bla*_*TEM*_ genes within *E. coli* ST131^46^. Our findings support this global data and shows that these resistance genes are both clonally and horizontally disseminated.

Unfortunately, the individual effects of the various mutations found in the QRDR of *parCE* and *gyrAB* as well as in *mgrB, pmrAB*, and *phoPQ*, in conferring resistance to fluoroquinolones and colistin could not ascertained in this study. This limitation makes it difficult to determine which resistance mechanism underlies the observed resistance, particularly as PMQR genes were also found in some of the isolates. The mutations observed in the *parCE* and *gyrAB* genes were not found in isolates that were reported from Durban, South Africa, except for R206L and E185D (in *gyrB*) and S458A in *parE*^14^. Similar studies in Portugal and India reported similar QRDR mutations^47,48^ (Table 2). We did not find *qnr* genes in the isolates, although a similar work in Durban reported several *qnr* variants^14,49,50^. However, the presence of *OqxAB* efflux genes have been reported in bacterial isolates from South Africa^14^.

*tet* genes are commonly reported from South Africa, Africa and worldwide on chromosomes or plasmids alongside *bla*_*CTX-M-15*,_ *aac(6*′)*Ib-cr, bla*_*OXA-10*_ and *bla*_*TEM*_^22,51–53^; specifically, the *tet(A/B)* genes in these isolates were mostly bracketed by Tn3 and composite transposons as well as by ISs. Despite chloramphenicol rarely being used to treat *E. coli* infections, several isolates contained the *cat* gene, indicating co-selection and/or transmission of chloramphenicol resistance genes by other antibiotics. Notably, all *catB3* genes were found as *catB3:bla*_*OXA-1*_*:aac(*6*’)-Ib-cr5* within composite transposons, suggesting that the use of fluoroquinolones, aminoglycosides and β-lactams could co-select and drive the dissemination of this resistance gene even in the absence of phenicols. Moreover, *cat* genes have been shown to be co-transmitted on plasmids with *aad* and *sul* genes through horizontal transmission and not natural selection^54^. Notably, *sul* and *aad* genes were identified in 9/11 (82%) of the isolates in which the *cat* gene was also identified.

The *sul* and *dfr* gene cassettes identified in the isolates were previously reported in a study done in *Enterobacteriaceae* in Tunisia^55^. However, these genes have only been reported in *Streptococcus pneumoniae* isolates from South Africa^56^, whilst the rare *sul3* gene was only recently reported in clinical isolates in Tanzania^57^. The genetic environment of *sul*1 *and sul2* genes were mostly consistent, with *sul*1 being mostly associated with *QacEΔ1* and *aadA* genes (*aadA1/5:QacEΔ1:sul1*) and *sul2* being always found with *aph* genes (*aph(6)-Id:aph(3*′)*-Ib:sul2*) within composite transposons or ISs. The association of these genes on the same MGEs might explain the co-resistance to SXT, chloramphenicol and macrolides in these strains as these antibiotics are not prescribed for treatment of infections caused by *E. coli* in South Africa.

Although *mph*(A), which is responsible for macrolide resistance, is not clinically important in *Enterobacteriaceae*, they can be transferred to medically important Gram-positive bacteria for which macrolides are indicated^58^. The *mph*(A) gene were normally found alongside *tetR* and IS*6* (*IS6::tetR::mph*(A)). The simultaneous presence of *cat, mph*(A) and *floR* genes in clinical *E. coli* isolates in South Africa has not been previously described, although similar findings were reported from Nigerian poultry and American calves^59,60^.

The frequency of class 1 integrons in these strains (95%) was much higher than isolates reported from Tunisia (64%), India (61%) and Korea (54%)^61–64^. The dominance of the *dfrA17* and *aadA5* cassettes, conferring resistance to trimethoprim and streptomycin, respectively, and their association with class 1 integrons has been described in several countries worldwide but not South Africa^26,62,65,66^. The isolates contained seven different cassette arrays, more than previously described from any single location^65^. We also identified the β-lactamase *bla*_*OXA-10*_ cassette in one isolate, which was previously described in a South China study^43^. We found no cassettes encoding *bla*_CTX-M_ and *bla*_TEM_, confirming that these genes are rarely spread by integrons. The integrons carrying the *dfrA5-psp-aadA2-cmlA1a-aadA1-qac* (E040) and *estX3-psp-aadA2-cmlA1a-aadA1a-qac* (K075) cassette arrays are the first to be described in Africa. There is a close similarity between the arrays of these two integrons, although their host strains were of different STs, and their resistomes and mobilomes were different (Tables 1 and 5); further analysis would be required to clarify this similarity.

As shown in Table 5, all the class 1 integrons were bracketed by ISs and composite transposons that can mobilize these resistance genes from plasmids to chromosomes and vice versa. The synteny and localization of several resistance genes within these MGEs suggest the presence of resistance genomic islands within the genomes. However, the transferability of these genes and MGEs were not experimentally ascertained, although the horizontal transmission of these resistance genes through MGEs within and across species cannot be entirely ruled out. Further, the close sequence identity of the contigs bearing the resistance genes and MGEs with already known plasmids and chromosomes confirms the location of these contigs on either chromosomes or plasmids.

The resolving power of WGS over MLST (multi-locus sequence typing) is clearly observed in Figs 1 and 2 in that strains of the same STs were found on different branches and nodes. The demographics, virulome, resistome, mobilome and genomic features of E005 and E009 as well as of E019 and E020 suggest that they might have originated from the same patients. Although 10 isolates were of ST131, only three (E062, E056 and E058) and two (E095 and E011) groups were phylogenetically related on the same clade, with the others clustering with other strains of different STs. These differences were further seen between this study’s isolates and those from Egypt and Tanzania, with which they closely clustered (Fig. 1). As seen in Fig. 2, they also varied in ST from those from the UK. These seeming discrepancies is due to the lower resolving power of MLST, which only uses seven house-keeping genes to type bacteria.

In addition, the difference in resistance genes between the closely clustered strains from South Africa and Egypt and Tanzania, further shows that not all these resistance genes were chromosomal. This is because the phylogenetic tree was drawn with the core genomes of the individual isolates without their accessory genomes (plasmids)^37^. The absence of any close relationship between the isolates in this study and other South African strains demonstrates the absence of an intra-country dissemination of *E. coli;* however, further investigations are necessary to confirm this assertion. Interestingly, the isolates were closely related to strains from Egypt, Tanzania and UK, suggesting the possible exchange of people between South Africa and these countries. Therefore, it is necessary for public health officials to screen patients coming from other countries (for medical tourism) for resistance genes to reduce the exchange of resistance genes across borders^3^.

The diversity and multiplicity of virulence genes found in these isolates, that were mainly obtained from blood and urine, is quite concerning. This is more so as the isolates were also MDR. Evidently, the small sample size of strains made it impossible to obtain a better association between specimen source and the virulome as suggested by Irenge *et al*.^67^. However, it is worthy of consideration, that isolates from the urine would also need virulence genes to initiate infection; hence, it is not surprising that the virulome of urine and blood isolates were comparable. Thus, it would be better to rather compare clinical with environmental strains in terms of virulence. The diversity and complexity of the virulome found in this study is quite comparable to that reported recently from the DRC^67^, although more virulence genes were reported in DRC than was observed herein.

The findings of this study present a worrying presence of a rich repertoire of resistance and virulence genes as well as MGEs in clonal and multiclonal *E. coli* strains within Pretoria. Although no carbapenemase, *mcr* and *tet(X3/4)* genes respectively mediating resistance to carbapenems, colistin and tigecycline were found, the chromosomally mediated colistin and tigecycline resistance in some of the strains is a cause for concern. We recommend additional molecular surveillance studies to provide statistically stronger data to inform pertinent interventions to contain these MDR strains from further dissemination.

### Ethical approval

Ethical approval was provided by the Human Research Ethics Committee of the University of Witwatersrand (Ref M1710100). All protocols and consent forms were executed according to the agreed ethical approval terms and conditions. All clinical samples were obtained from a reference laboratory and not directly from patients, who agreed to our using their specimens for this research. The guidelines stated by the Declaration of Helsinki for involving human participants were followed in the study.

## Supplementary Information

Supplementary information

Supplementary information

Supplementary information

Supplementary information

## References

[1] Dijkshoorn L, Nemec A, Seifert H (2007). An increasing threat in hospitals: multidrug-resistant Acinetobacter baumannii. Nat. Rev. Microbiol..

[2] Osei Sekyere J (2014). Antibiotic types and handling practices in disease management among pig farms in Ashanti Region, Ghana. J. Vet. Med..

[3] Osei Sekyere J (2016). Current State of Resistance to Antibiotics of Last-Resort in South Africa: A Review from a Public Health. Perspective. Front. Public Heal..

[4] WHO. *Global Antimicrobial Resistance Surveillance System (GLASS) Report*, 10.1016/S1473-3099(18)30060-4 (2017).

[5] WHO. *Antimicrobial Resistance: Global Report on surveillance*. (World Health Organisation, 2014).

[6] Chirindze LM (2018). Faecal colonization of E. coli and Klebsiella spp. producing extended-spectrum beta-lactamases and plasmid-mediated AmpC in Mozambican university students. BMC Infect. Dis..

[7] Osei Sekyere J, Asante J (2018). Emerging mechanisms of antimicrobial resistance in bacteria and fungi: advances in the era of genomics. Future Microbiol..

[8] Asante, J. & Osei Sekyere, J. Understanding antimicrobial discovery and resistance from a metagenomic and metatranscriptomic perspective: Advances and applications. *Environ. Microbiol. Rep*. 1–25, 10.1111/1758-2229.12735 (2019). 10.1111/1758-2229.12735 30637962

[9] Poirel, L. *et al*. Antimicrobial Resistance in Escherichia coli. *Microbiol. Spectr*. **6** (2018). 10.1128/microbiolspec.arba-0026-2017 PMC11633601 30003866

[10] Viazis S, Akhtar M, Feirtag J, Diez-Gonzalez F (2011). Reduction of Escherichia coli O157:H7 viability on leafy green vegetables by treatment with a bacteriophage mixture and trans-cinnamaldehyde. Food Microbiol..

[11] Gerhardts A, Hammer TR, Balluff C, Mucha H, Hoefer D (2012). A model of the transmission of micro-organisms in a public setting and its correlation to pathogen infection risks. J. Appl. Microbiol..

[12] Poirel L (2016). Genetic Features of MCR-1-Producing Colistin-Resistant Escherichia coli Isolates in South Africa. Antimicrob. Agents Chemother..

[13] Mhlongo N, Essack S, Govinden U (2015). NDM-1, novel TEM-205, novel TEM-213 and other extended-spectrum β-lactamases co-expressed in isolates from cystic fibrosis patients from South Africa. South. African J. Infect. Dis..

[14] Osei Sekyere J, Amoako DG (2017). Genomic and Phenotypic Characterisation of Fluoroquinolone Resistance Mechanisms in Enterobacteriaceae in Durban, South Africa. PLoS One.

[15] Abia ALK, Ubomba-Jaswa E, Momba MNB (2015). High prevalence of multiple-antibiotic-resistant (MAR) Escherichia coli in river bed sediments of the Apies River, South Africa. Environ. Monit. Assess..

[16] Teklehaimanot GZ, Coetzee MAA, Momba MNB (2014). Faecal pollution loads in the wastewater effluents and receiving water bodies: a potential threat to the health of Sedibeng and Soshanguve communities, South Africa. Environ. Sci. Pollut. Res. Int..

[17] Gqunta K, Govender S (2015). Characterization of ESBL-producing Escherichia coli ST131 isolates from Port Elizabeth. Diagn. Microbiol. Infect. Dis..

[18] Habte TM, Dube S, Ismail N, Hoosen AA (2009). Hospital and community isolates of uropathogens at a tertiary hospital in South Africa. S. Afr. Med. J..

[19] Stokes HW, Gillings MR (2011). Gene flow,mobile genetic elements and the recruitment of antibiotic resistance genes into Gram-negative pathogens. FEMS Microbiol. Rev..

[20] Kopotsa, K., Osei Sekyere, J. & Mbelle, N. M. Plasmid Evolution in Carbapenemase-Producing Enterobacteriaceae: *A Review. Ann*. New Accepted, (2019). 10.1111/nyas.14223 31469443

[21] Pedersen, T. *et al*. Spread of Plasmid-Encoded NDM-1 and GES-5 Carbapenemases among Extensively Drug-Resistant and Pandrug-Resistant Clinical Enterobacteriaceae in Durban, South Africa. *Antimicrob. Agents Chemother*. AAC.02178–17, 10.1128/AAC.02178-17 (2018). 10.1128/AAC.02178-17 PMC5923139 29507063

[22] Mbelle, N. M. *et al*. Genomic analysis of a multidrug-resistant clinical Providencia rettgeri (PR002) strain with the novel integron ln1483 and an A/C plasmid replicon. *Ann. N. Y. Acad. Sci*. **Accepted** (2019). 10.1111/nyas.14237 31549428

[23] Mbelle, N. *et al*. Genomic Analysis of Two Drug‐Resistant Clinical Morganella morganii Strains Isolated from UTI Patients in Pretoria, South Africa. *Lett. Appl. Microbiol*. Accepted, lam.13237 (2019). 10.1111/lam.13237 31630429

[24] Carattoli A (2009). Resistance Plasmid Families in Enterobacteriaceae. Antimicrob. Agents Chemother..

[25] Boucher Y, Labbate M, Koenig JE, Stokes HW (2007). Integrons: mobilizable platforms that promote genetic diversity in bacteria. Trends Microbiol..

[26] Partridge SR (2011). Analysis of antibiotic resistance regions in Gram-negative bacteria. FEMS Microbiol. Rev..

[27] Partridge SR, Kwong SM, Firth N, Jensen SO (2018). Mobile Genetic Elements Associated with Antimicrobial Resistance. Clin. Microbiol. Rev..

[28] Cambray G, Guerout A-M, Mazel D (2010). Integrons. Annu. Rev. Genet..

[29] Clinical and Laboratory Standards Institute (CLSI). *Performance standards for Antimicrobial Susceptibility Testing; Twenty-Seventh Informational Supplement M100-S27*. (CLSI, Wayne, PA, USA, 2019).

[30] Osei Sekyere, J. *Mcr* colistin resistance gene: a systematic review of current diagnostics and detection methods. *Microbiologyopen* e00682, 10.1002/mbo3.682 (2018). 10.1002/mbo3.682 PMC6530528 29974640

[31] Osei Sekyere J, Govinden U, Bester LA, Essack SY (2016). Colistin and Tigecycline Resistance In Carbapenemase-Producing Gram Negative Bacteria: Emerging Resistance Mechanisms And Detection Methods. J. Appl. Microbiol..

[32] European Committee on Antimicrobial Susceptibility Testing. *Breakpoint tables for interpretation of MICs and zone diameters. Version 9.0, 2019*, http://www.eucast.org/fileadmin/src/media/PDFs/EUCAST_files/Breakpoint_tables/v_9.0_Breakpoint_Tables.pdf (2019).

[33] Mbelle NM (2017). First Report of a Whole-Genome Shotgun Sequence of a Clinical Enterococcus faecalis Sequence Type 6 Strain from South Africa. Genome Announc..

[34] Mbelle NM (2017). Draft Genome Sequence of a Clinical Enterococcus faecium Sequence Type 18 Strain from South Africa. Genome Announc..

[35] Tatusova, T. *et al*. NCBI prokaryotic genome annotation pipeline. **44**, 6614–6624 (2016). 10.1093/nar/gkw569 PMC5001611 27342282

[36] Treangen TJ, Ondov BD, Koren S, Phillippy AM (2014). The Harvest suite for rapid core-genome alignment and visualization of thousands of intraspecific microbial genomes. Genome Biol..

[37] Ruan Z, Feng Y (2016). BacWGSTdb, a database for genotyping and source tracking bacterial pathogens. Nucleic Acids Res..

[38] Osei Sekyere, J. Genomic insights into nitrofurantoin resistance mechanisms and epidemiology in clinical Enterobacteriaceae. *Futur. Sci. OA* **4**, FSO293 (2018). 10.4155/fsoa-2017-0156 PMC5961450 29796297

[39] He T (2019). Emergence of plasmid-mediated high-level tigecycline resistance genes in animals and humans. Nat. Microbiol.

[40] Jacoby, G. A. AmpC beta-lactamases. *Clin. Microbiol. Rev*. **22**, 161–82, Table of Contents (2009). 10.1128/CMR.00036-08 PMC2620637 19136439

[41] Arpin C (2012). Evolution of an incompatibility group IncA/C plasmid harboring blaCMY-16 and qnrA6 genes and its transfer through three clones of Providencia stuartii during a two-year outbreak in a Tunisian burn unit. Antimicrob. Agents Chemother..

[42] Peirano G, van Greune CHJ, Pitout JDD (2011). Characteristics of infections caused by extended-spectrum β-lactamase–producing Escherichia coli from community hospitals in South Africa. Diagn. Microbiol. Infect. Dis..

[43] Sun J (2013). Class 1 Integrons in Urinary Isolates of Extended-Spectrum β-Lactamase-Producing *Escherichia coli* and *Klebsiella pneumoniae* in Southern China During the Past Five Years. Microb. Drug Resist..

[44] Cao X (2014). Genotypic characteristics of multidrug-resistant *Escherichia coli* isolates associated with urinary tract infections. APMIS.

[45] Peirano G, Pitout JDD (2010). Molecular epidemiology of Escherichia coli producing CTX-M β-lactamases: the worldwide emergence of clone ST131 O25:H4. Int. J. Antimicrob. Agents.

[46] Coque TM, Novais A, Caratolli A (2008). & al., et. Dissemination of clonally related Escherichia coli strains expressing extended-spectrum β-lactamase CTX-M-15. Emerg Infect Dis.

[47] Correia S (2012). High prevalence of ESBL-producing Escherichia coli isolates among hemodialysis patients in Portugal: appearance of ST410 with the blaCTX-M-14 gene. Diagn. Microbiol. Infect. Dis..

[48] Pathak A, Chandran SP, Mahadik K, Macaden R, Lundborg CS (2013). Frequency and factors associated with carriage of multi-drug resistant commensal Escherichia coliamong women attending antenatal clinics in Central India. BMC Infect. Dis..

[49] Chenia HY (2016). Prevalence and characterization of plasmid-mediated quinolone resistance genes in Aeromonas spp. isolated from South African freshwater fish. Int. J. Food Microbiol..

[50] Chenia H, Pillay B, Pillay D (2006). Analysis of the mechanisms of fluoroquinolone resistance in urinary tract pathogens. J Antimicrob Chemother.

[51] Adefisoye MA, Okoh AI (2016). Identification and antimicrobial resistance prevalence of pathogenic *Escherichia coli* strains from treated wastewater effluents in Eastern Cape, South Africa. Microbiologyopen.

[52] McMillan EA (2019). Antimicrobial Resistance Genes, Cassettes, and Plasmids Present in Salmonella enterica Associated With United States Food Animals. Front. Microbiol..

[53] Jeong S (2018). Extensively Drug-Resistant Escherichia coli Sequence Type 1642 Carrying an IncX3 Plasmid Containing the blaKPC-2 Gene Associated with Transposon Tn4401a. Ann. Lab. Med..

[54] Zhang X-X, Zhang T, Fang HHP (2009). Antibiotic resistance genes in water environment. Appl. Microbiol. Biotechnol..

[55] Dahmen S, Mansour W, Boujaafar N, Arlet G, Bouallègue O (2010). Distribution of Cotrimoxazole Resistance Genes Associated with Class 1 Integrons in Clinical Isolates of Enterobacteriaceae in a University Hospital in Tunisia. Microb. Drug Resist..

[56] Adrian PV, Klugman KP (1997). Mutations in the dihydrofolate reductase gene of trimethoprim-resistant isolates of Streptococcus pneumoniae. Antimicrob. Agents Chemother..

[57] Manyahi J (2017). Molecular Characterization of Cotrimoxazole Resistance Genes and Their Associated Integrons in Clinical Isolates of Gram-Negative Bacteria from Tanzania. Microb. Drug Resist..

[58] Jones-Dias D (2016). Architecture of Class 1, 2, and 3 Integrons from Gram Negative Bacteria Recovered among Fruits and Vegetables. Front. Microbiol..

[59] Nsofor A (2013). Detection of antibiotic resistance genes of Escherichia coli from domestic livestock in south east Nigeria with DNA microarray. J. Cell Anim. Biol..

[60] Davis MA (2011). Genotypic-phenotypic discrepancies between antibiotic resistance characteristics of Escherichia coli isolates from calves in management settings with high and low antibiotic use. Appl. Environ. Microbiol..

[61] Ben Slama K (2011). Diversity of Genetic Lineages Among CTX-M-15 and CTX-M-14 Producing Escherichia coli Strains in a Tunisian Hospital. Curr. Microbiol..

[62] Akram M, Shakil S, Khan AU (2011). Prevalence of Integrons, *bla*_CTX-M_ and *bla*_TEM_ Resistance Markers among ESBL-Producing Uropathogenic *Escherichia coli* Isolates: First Report of Genomic *bla*_CTX-M_ from India. J. Chemother..

[63] Mac Aogáin M, Mooij MJ, Adams C, Clair J, O’Gara F (2010). Emergence of extended-spectrum β-lactamase and fluoroquinolone resistance genes among Irish multidrug-resistant isolates. Diagn. Microbiol. Infect. Dis..

[64] Yu HS (2003). Changes in gene cassettes of class 1 integrons among Escherichia coli isolates from urine specimens collected in Korea during the last two decades. J. Clin. Microbiol..

[65] Nielsen KM, Domingues S, da Silva GJ (2015). Global dissemination patterns of common gene cassette arrays in class 1 integrons. Microbiology.

[66] Wei Q (2013). Diversity of Gene Cassette Promoter Variants of Class 1 Integrons in Uropathogenic Escherichia coli. Curr. Microbiol..

[67] Irenge LM (2019). Whole-genome sequences of multidrug-resistant Escherichia coli in South-Kivu Province, Democratic Republic of Congo: characterization of phylogenomic changes, virulence and resistance genes. BMC Infect. Dis..

