# Publisher Correction: The Resistome, Mobilome, Virulome and Phylogenomics of Multidrug-Resistant *Escherichia coli* Clinical Isolates from Pretoria, South Africa

**DOI:** 10.1038/s41598-020-58160-x

**Published:** 2020-01-22

**Authors:** Nontombi Marylucy Mbelle, Charles Feldman, John Osei Sekyere, Nontuthuko Excellent Maningi, Lesedi Modipane, Sabiha Yusuf Essack

**Affiliations:** 1 Department of Medical Microbiology, Faculty of Health Sciences, University of Pretoria, Pretoria, South Africa; 2 National Health Laboratory Service, Tshwane Academic Division, Pretoria, South Africa; 3 Department of Internal Medicine, Faculty of Health Sciences, University of the Witwatersrand, Johannesburg, South Africa; 4 Antimicrobial Research Unit, College of Health Sciences, University of KwaZulu/Natal, Durban, South Africa

Correction to: *Scientific Reports* 10.1038/s41598-019-52859-2, published online 11 November 2019

The Author information section in this Article is incorrect.

“These author contributed equally: Charles Feldman and Sabiha Yusuf Essack

These authors jointly supervised this work: Nontombi Marylucy Mbelle and John Osei Sekyere.”

should read:

“These authors contributed equally: Nontombi Marylucy Mbelle and John Osei Sekyere

These authors jointly supervised this work: Charles Feldman and Sabiha Yusuf Essack.”

