# Supplementary material for: The Resistome, Mobilome, Virulome and Phylogenomics of Multidrug-Resistant Escherichia coli Clinical Isolates from Pretoria, South Africa
Source: Sci Rep. 2019 Nov 11;9:16457. doi: 10.1038/s41598-019-52859-2 (PMC6848087; doi:10.1038/s41598-019-52859-2)
Supplement: Supplementary file 4 — Supplementary information [file 41598_2019_52859_MOESM4_ESM.docx]

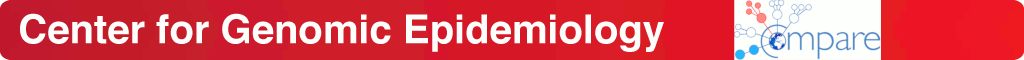


|  | Home | Services | Instructions | Output | Overview of genes | Article abstract |  |
| --- | --- | --- | --- | --- | --- | --- | --- |

**ResFinder-3.2 Server - Results**

**Input Files: *E005.fasta***

Show Acquired antimicrobial resistance results

**Acquired antimicrobial resistance gene - Results**

| **Rifampicin** | | | | | | |
| --- | --- | --- | --- | --- | --- | --- |
| **Resistance gene** | **Identity** | **Query / Template length** | **Contig** | **Position in contig** | **Predicted phenotype** | **Accession number** |
| No hit found | | | | | | |

| **Glycopeptide** | | | | | | |
| --- | --- | --- | --- | --- | --- | --- |
| **Resistance gene** | **Identity** | **Query / Template length** | **Contig** | **Position in contig** | **Predicted phenotype** | **Accession number** |
| No hit found | | | | | | |

| **Tetracycline** | | | | | | |
| --- | --- | --- | --- | --- | --- | --- |
| **Resistance gene** | **Identity** | **Query / Template length** | **Contig** | **Position in contig** | **Predicted phenotype** | **Accession number** |
| tet(A) | 99.92 | 1200 / 1200 | NXLF01000051.1 Escherichia coli strain ST-131:E005 NODE_51_length_7793_cov_38.7485_ID_101, whole genome shotgun sequence | 4150..5349 | Tetracycline resistance | [AJ517790](http://www.ncbi.nlm.nih.gov/nuccore/AJ517790) |

| **Colistin** | | | | | | |
| --- | --- | --- | --- | --- | --- | --- |
| **Resistance gene** | **Identity** | **Query / Template length** | **Contig** | **Position in contig** | **Predicted phenotype** | **Accession number** |
| No hit found | | | | | | |

| **Macrolide** | | | | | | |
| --- | --- | --- | --- | --- | --- | --- |
| **Resistance gene** | **Identity** | **Query / Template length** | **Contig** | **Position in contig** | **Predicted phenotype** | **Accession number** |
| mdf(A) | 97.81 | 1233 / 1233 | NXLF01000012.1 Escherichia coli strain ST-131:E005 NODE_12_length_151655_cov_50.7131_ID_23, whole genome shotgun sequence | 50536..51768 | Warning: gene is missing from Notes file. Please inform curator. | [Y08743](http://www.ncbi.nlm.nih.gov/nuccore/Y08743) |
| mph(A) | 99.89 | 906 / 906 | NXLF01000052.1 Escherichia coli strain ST-131:E005 NODE_52_length_7696_cov_47.1951_ID_103, whole genome shotgun sequence | 6624..7528 | Macrolide resistance | [D16251](http://www.ncbi.nlm.nih.gov/nuccore/D16251) |

| **Phenicol** | | | | | | |
| --- | --- | --- | --- | --- | --- | --- |
| **Resistance gene** | **Identity** | **Query / Template length** | **Contig** | **Position in contig** | **Predicted phenotype** | **Accession number** |
| catB3 | 100 | 442 / 633 | NXLF01000074.1 Escherichia coli strain ST-131:E005 NODE_74_length_2383_cov_117.947_ID_147, whole genome shotgun sequence | 99..540 | Phenicol resistance | [AJ009818](http://www.ncbi.nlm.nih.gov/nuccore/AJ009818) |
| catB3 | 100 | 442 / 633 | NXLF01000074.1 Escherichia coli strain ST-131:E005 NODE_74_length_2383_cov_117.947_ID_147, whole genome shotgun sequence | 99..540 | Phenicol resistance | [U13880](http://www.ncbi.nlm.nih.gov/nuccore/U13880) |

| **Beta-lactam** | | | | | | |
| --- | --- | --- | --- | --- | --- | --- |
| **Resistance gene** | **Identity** | **Query / Template length** | **Contig** | **Position in contig** | **Predicted phenotype** | **Accession number** |
| blaCTX-M-15 | 100 | 876 / 876 | NXLF01000067.1 Escherichia coli strain ST-131:E005 NODE_67_length_2972_cov_216.032_ID_133, whole genome shotgun sequence | 1706..2581 | Beta-lactam resistance Alternate name; UOE-1 | [AY044436](http://www.ncbi.nlm.nih.gov/nuccore/AY044436) |
| blaOXA-1 | 100 | 831 / 831 | NXLF01000074.1 Escherichia coli strain ST-131:E005 NODE_74_length_2383_cov_117.947_ID_147, whole genome shotgun sequence | 678..1508 | Beta-lactam resistance | [HQ170510](http://www.ncbi.nlm.nih.gov/nuccore/HQ170510) |
| blaTEM-1B | 100 | 861 / 861 | NXLF01000050.1 Escherichia coli strain ST-131:E005 NODE_50_length_8371_cov_133.702_ID_99, whole genome shotgun sequence | 236..1096 | Beta-lactam resistance Alternate name; RblaTEM-1 | [AY458016](http://www.ncbi.nlm.nih.gov/nuccore/AY458016) |

| **Aminoglycoside** | | | | | | |
| --- | --- | --- | --- | --- | --- | --- |
| **Resistance gene** | **Identity** | **Query / Template length** | **Contig** | **Position in contig** | **Predicted phenotype** | **Accession number** |
| aac(3)-IIa | 99.77 | 861 / 861 | NXLF01000068.1 Escherichia coli strain ST-131:E005 NODE_68_length_2797_cov_68.1042_ID_135, whole genome shotgun sequence | 1795..2655 | Aminoglycoside resistance | [X51534](http://www.ncbi.nlm.nih.gov/nuccore/X51534) |
| aac(3)-IId | 99.83 | 583 / 861 | NXLF01000097.1 Escherichia coli strain ST-131:E005 NODE_97_length_848_cov_153.244_ID_193, whole genome shotgun sequence | 266..848 | Aminoglycoside resistance | [EU022314](http://www.ncbi.nlm.nih.gov/nuccore/EU022314) |
| aac(6')-Ib-cr | 100 | 600 / 600 | NXLF01000074.1 Escherichia coli strain ST-131:E005 NODE_74_length_2383_cov_117.947_ID_147, whole genome shotgun sequence | 1639..2238 | Fluoroquinolone and aminoglycoside resistance | [DQ303918](http://www.ncbi.nlm.nih.gov/nuccore/DQ303918) |
| aadA5 | 100 | 789 / 789 | NXLF01000070.1 Escherichia coli strain ST-131:E005 NODE_70_length_2654_cov_68.6297_ID_139, whole genome shotgun sequence | 263..1051 | Aminoglycoside resistance | [AF137361](http://www.ncbi.nlm.nih.gov/nuccore/AF137361) |
| aph(3'')-Ib | 100 | 804 / 804 | NXLF01000051.1 Escherichia coli strain ST-131:E005 NODE_51_length_7793_cov_38.7485_ID_101, whole genome shotgun sequence | 1457..2260 | Aminoglycoside resistance Alternate name; aph(3'')-Ib | [AF321551](http://www.ncbi.nlm.nih.gov/nuccore/AF321551) |
| aph(6)-Id | 100 | 831 / 831 | NXLF01000051.1 Escherichia coli strain ST-131:E005 NODE_51_length_7793_cov_38.7485_ID_101, whole genome shotgun sequence | 2266..3096 | Aminoglycoside resistance Alternate name; aph(6)-Id | [CP000971](http://www.ncbi.nlm.nih.gov/nuccore/CP000971) |

| **Fusidicacid** | | | | | | |
| --- | --- | --- | --- | --- | --- | --- |
| **Resistance gene** | **Identity** | **Query / Template length** | **Contig** | **Position in contig** | **Predicted phenotype** | **Accession number** |
| No hit found | | | | | | |

| **Fosfomycin** | | | | | | |
| --- | --- | --- | --- | --- | --- | --- |
| **Resistance gene** | **Identity** | **Query / Template length** | **Contig** | **Position in contig** | **Predicted phenotype** | **Accession number** |
| No hit found | | | | | | |

| **Nitroimidazole** | | | | | | |
| --- | --- | --- | --- | --- | --- | --- |
| **Resistance gene** | **Identity** | **Query / Template length** | **Contig** | **Position in contig** | **Predicted phenotype** | **Accession number** |
| No hit found | | | | | | |

| **Sulphonamide** | | | | | | |
| --- | --- | --- | --- | --- | --- | --- |
| **Resistance gene** | **Identity** | **Query / Template length** | **Contig** | **Position in contig** | **Predicted phenotype** | **Accession number** |
| sul1 | 100 | 840 / 840 | NXLF01000052.1 Escherichia coli strain ST-131:E005 NODE_52_length_7696_cov_47.1951_ID_103, whole genome shotgun sequence | 474..1313 | Sulphonamide resistance | [U12338](http://www.ncbi.nlm.nih.gov/nuccore/U12338) |
| sul2 | 100 | 816 / 816 | NXLF01000051.1 Escherichia coli strain ST-131:E005 NODE_51_length_7793_cov_38.7485_ID_101, whole genome shotgun sequence | 581..1396 | Sulphonamide resistance | [AY034138](http://www.ncbi.nlm.nih.gov/nuccore/AY034138) |

| **Trimethoprim** | | | | | | |
| --- | --- | --- | --- | --- | --- | --- |
| **Resistance gene** | **Identity** | **Query / Template length** | **Contig** | **Position in contig** | **Predicted phenotype** | **Accession number** |
| dfrA17 | 100 | 474 / 474 | NXLF01000070.1 Escherichia coli strain ST-131:E005 NODE_70_length_2654_cov_68.6297_ID_139, whole genome shotgun sequence | 1182..1655 | Trimethoprim resistance | [FJ460238](http://www.ncbi.nlm.nih.gov/nuccore/FJ460238) |

| **Oxazolidinone** | | | | | | |
| --- | --- | --- | --- | --- | --- | --- |
| **Resistance gene** | **Identity** | **Query / Template length** | **Contig** | **Position in contig** | **Predicted phenotype** | **Accession number** |
| No hit found | | | | | | |

| **Quinolone** | | | | | | |
| --- | --- | --- | --- | --- | --- | --- |
| **Resistance gene** | **Identity** | **Query / Template length** | **Contig** | **Position in contig** | **Predicted phenotype** | **Accession number** |
| aac(6')-Ib-cr | 100 | 600 / 600 | NXLF01000074.1 Escherichia coli strain ST-131:E005 NODE_74_length_2383_cov_117.947_ID_147, whole genome shotgun sequence | 1639..2238 | Fluoroquinolone and aminoglycoside resistance | [DQ303918](http://www.ncbi.nlm.nih.gov/nuccore/DQ303918) |

extended output

**Selected %ID threshold:  *90 %***

**Selected minimum length:  *60 %***

Top of Form

Bottom of Form

Top of Form

Bottom of Form

Top of Form

Bottom of Form

Top of Form

Bottom of Form

Show Point mutation results

**Chromosomal point mutations - Results**

**Species:*escherichia_coli***

**Known Mutations**

| **16S_rrsC** | | | | |
| --- | --- | --- | --- | --- |
| No known mutations found in 16S_rrsC |  |  |  |  |

| **gyrB** | | | | |
| --- | --- | --- | --- | --- |
| No known mutations found in gyrB |  |  |  |  |

| **16S_rrsB** | | | | |
| --- | --- | --- | --- | --- |
| No known mutations found in 16S_rrsB |  |  |  |  |

| **16S_rrsH** | | | | |
| --- | --- | --- | --- | --- |
| No known mutations found in 16S_rrsH |  |  |  |  |

| **ampC** | | | | |
| --- | --- | --- | --- | --- |
| No known mutations found in ampC |  |  |  |  |

| **parE** | | | | |
| --- | --- | --- | --- | --- |
| **Mutation** | **Nucleotide change** | **Amino acid change** | **Resistance** | **PMID** |
| parE p.I529L | ATT ➝ CTT | I ➝ L | Nalidixic acid,Ciprofloxacin | [14506034](http://www.ncbi.nlm.nih.gov/pubmed/14506034) |

| **23S** | | | | |
| --- | --- | --- | --- | --- |
| No known mutations found in 23S |  |  |  |  |

| **pmrB** | | | | |
| --- | --- | --- | --- | --- |
| No known mutations found in pmrB |  |  |  |  |

| **pmrA** | | | | |
| --- | --- | --- | --- | --- |
| No known mutations found in pmrA |  |  |  |  |

| **gyrA** | | | | |
| --- | --- | --- | --- | --- |
| **Mutation** | **Nucleotide change** | **Amino acid change** | **Resistance** | **PMID** |
| gyrA p.S83L | TCG ➝ TTG | S ➝ L | Nalidixic acid,Ciprofloxacin | [8891148](http://www.ncbi.nlm.nih.gov/pubmed/8891148) |
| gyrA p.D87N | GAC ➝ AAC | D ➝ N | Nalidixic acid,Ciprofloxacin | [12654733](http://www.ncbi.nlm.nih.gov/pubmed/12654733) |

| **rpoB** | | | | |
| --- | --- | --- | --- | --- |
| No known mutations found in rpoB |  |  |  |  |

| **parC** | | | | |
| --- | --- | --- | --- | --- |
| **Mutation** | **Nucleotide change** | **Amino acid change** | **Resistance** | **PMID** |
| parC p.S80I | AGC ➝ ATT | S ➝ I | Nalidixic acid,Ciprofloxacin | [8851598](http://www.ncbi.nlm.nih.gov/pubmed/8851598) |
| parC p.E84V | GAA ➝ GTA | E ➝ V | Nalidixic acid,Ciprofloxacin | [12654733](http://www.ncbi.nlm.nih.gov/pubmed/12654733) |

| **folP** | | | | |
| --- | --- | --- | --- | --- |
| No known mutations found in folP |  |  |  |  |

Top of Form

Bottom of Form

Top of Form

Bottom of Form

**CITATIONS**

For publication of results, please cite:

- Identification of acquired antimicrobial resistance genes.
  Zankari E, Hasman H, Cosentino S, Vestergaard M, Rasmussen S, Lund O, Aarestrup FM, Larsen MV.
  J Antimicrob Chemother. 2012 Jul 10.
  PMID: [22782487](http://www.ncbi.nlm.nih.gov/pubmed/22782487)         doi: [10.1093/jac/dks261](http://dx.doi.org/10.1093/jac/dks261)


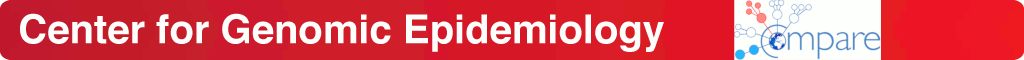


|  | Home | Services | Instructions | Output | Overview of genes | Article abstract |  |
| --- | --- | --- | --- | --- | --- | --- | --- |

**ResFinder-3.2 Server - Results**

**Input Files: *E003.fasta***

Show Acquired antimicrobial resistance results

**Acquired antimicrobial resistance gene - Results**

| **Macrolide** | | | | | | |
| --- | --- | --- | --- | --- | --- | --- |
| **Resistance gene** | **Identity** | **Query / Template length** | **Contig** | **Position in contig** | **Predicted phenotype** | **Accession number** |
| mdf(A) | 99.92 | 1233 / 1233 | NXIZ01000009.1 Escherichia coli strain E003 NODE_9_length_143897_cov_47.6126, whole genome shotgun sequence | 12037..13269 | Warning: gene is missing from Notes file. Please inform curator. | [Y08743](http://www.ncbi.nlm.nih.gov/nuccore/Y08743) |

| **Tetracycline** | | | | | | |
| --- | --- | --- | --- | --- | --- | --- |
| **Resistance gene** | **Identity** | **Query / Template length** | **Contig** | **Position in contig** | **Predicted phenotype** | **Accession number** |
| tet(B) | 100 | 1206 / 1206 | NXIZ01000110.1 Escherichia coli strain E003 NODE_110_length_2799_cov_25.8559, whole genome shotgun sequence | 517..1722 | Tetracycline resistance | [AF326777](http://www.ncbi.nlm.nih.gov/nuccore/AF326777) |

| **Nitroimidazole** | | | | | | |
| --- | --- | --- | --- | --- | --- | --- |
| **Resistance gene** | **Identity** | **Query / Template length** | **Contig** | **Position in contig** | **Predicted phenotype** | **Accession number** |
| No hit found | | | | | | |

| **Fosfomycin** | | | | | | |
| --- | --- | --- | --- | --- | --- | --- |
| **Resistance gene** | **Identity** | **Query / Template length** | **Contig** | **Position in contig** | **Predicted phenotype** | **Accession number** |
| No hit found | | | | | | |

| **Aminoglycoside** | | | | | | |
| --- | --- | --- | --- | --- | --- | --- |
| **Resistance gene** | **Identity** | **Query / Template length** | **Contig** | **Position in contig** | **Predicted phenotype** | **Accession number** |
| aadA5 | 100 | 789 / 789 | NXIZ01000073.1 Escherichia coli strain E003 NODE_73_length_11594_cov_47.2424, whole genome shotgun sequence | 7638..8426 | Aminoglycoside resistance | [AF137361](http://www.ncbi.nlm.nih.gov/nuccore/AF137361) |
| aph(3'')-Ib | 100 | 804 / 804 | NXIZ01000099.1 Escherichia coli strain E003 NODE_99_length_4640_cov_45.1682, whole genome shotgun sequence | 972..1775 | Aminoglycoside resistance Alternate name; aph(3'')-Ib | [AF321551](http://www.ncbi.nlm.nih.gov/nuccore/AF321551) |
| aph(6)-Id | 100 | 837 / 837 | NXIZ01000099.1 Escherichia coli strain E003 NODE_99_length_4640_cov_45.1682, whole genome shotgun sequence | 136..972 | Aminoglycoside resistance Alternate name; aph(6)-Id | [M28829](http://www.ncbi.nlm.nih.gov/nuccore/M28829) |

| **Quinolone** | | | | | | |
| --- | --- | --- | --- | --- | --- | --- |
| **Resistance gene** | **Identity** | **Query / Template length** | **Contig** | **Position in contig** | **Predicted phenotype** | **Accession number** |
| No hit found | | | | | | |

| **Beta-lactam** | | | | | | |
| --- | --- | --- | --- | --- | --- | --- |
| **Resistance gene** | **Identity** | **Query / Template length** | **Contig** | **Position in contig** | **Predicted phenotype** | **Accession number** |
| blaCMY-2 | 100 | 1146 / 1146 | NXIZ01000036.1 Escherichia coli strain E003 NODE_36_length_35478_cov_71.7592, whole genome shotgun sequence | 9202..10347 | Beta-lactam resistance | [X91840](http://www.ncbi.nlm.nih.gov/nuccore/X91840) |

| **Rifampicin** | | | | | | |
| --- | --- | --- | --- | --- | --- | --- |
| **Resistance gene** | **Identity** | **Query / Template length** | **Contig** | **Position in contig** | **Predicted phenotype** | **Accession number** |
| No hit found | | | | | | |

| **Trimethoprim** | | | | | | |
| --- | --- | --- | --- | --- | --- | --- |
| **Resistance gene** | **Identity** | **Query / Template length** | **Contig** | **Position in contig** | **Predicted phenotype** | **Accession number** |
| dfrA17 | 100 | 474 / 474 | NXIZ01000073.1 Escherichia coli strain E003 NODE_73_length_11594_cov_47.2424, whole genome shotgun sequence | 7034..7507 | Trimethoprim resistance | [FJ460238](http://www.ncbi.nlm.nih.gov/nuccore/FJ460238) |

| **Phenicol** | | | | | | |
| --- | --- | --- | --- | --- | --- | --- |
| **Resistance gene** | **Identity** | **Query / Template length** | **Contig** | **Position in contig** | **Predicted phenotype** | **Accession number** |
| catA1 | 99.85 | 660 / 660 | NXIZ01000073.1 Escherichia coli strain E003 NODE_73_length_11594_cov_47.2424, whole genome shotgun sequence | 351..1010 | Phenicol resistance | [V00622](http://www.ncbi.nlm.nih.gov/nuccore/V00622) |

| **Sulphonamide** | | | | | | |
| --- | --- | --- | --- | --- | --- | --- |
| **Resistance gene** | **Identity** | **Query / Template length** | **Contig** | **Position in contig** | **Predicted phenotype** | **Accession number** |
| sul1 | 100 | 840 / 840 | NXIZ01000073.1 Escherichia coli strain E003 NODE_73_length_11594_cov_47.2424, whole genome shotgun sequence | 8973..9812 | Sulphonamide resistance | [U12338](http://www.ncbi.nlm.nih.gov/nuccore/U12338) |
| sul2 | 100 | 816 / 816 | NXIZ01000099.1 Escherichia coli strain E003 NODE_99_length_4640_cov_45.1682, whole genome shotgun sequence | 1836..2651 | Sulphonamide resistance | [HQ840942](http://www.ncbi.nlm.nih.gov/nuccore/HQ840942) |

| **Colistin** | | | | | | |
| --- | --- | --- | --- | --- | --- | --- |
| **Resistance gene** | **Identity** | **Query / Template length** | **Contig** | **Position in contig** | **Predicted phenotype** | **Accession number** |
| No hit found | | | | | | |

| **Glycopeptide** | | | | | | |
| --- | --- | --- | --- | --- | --- | --- |
| **Resistance gene** | **Identity** | **Query / Template length** | **Contig** | **Position in contig** | **Predicted phenotype** | **Accession number** |
| No hit found | | | | | | |

extended output

**Selected %ID threshold:  *90 %***

**Selected minimum length:  *60 %***

Top of Form

Bottom of Form

Top of Form

Bottom of Form

Top of Form

Bottom of Form

Top of Form

Bottom of Form

Show Point mutation results

**Chromosomal point mutations - Results**

**Species:*escherichia_coli***

**Known Mutations**

| **folP** | | | | |
| --- | --- | --- | --- | --- |
| No mutations found in folP |  |  |  |  |

| **pmrA** | | | | |
| --- | --- | --- | --- | --- |
| No mutations found in pmrA |  |  |  |  |

| **16S_rrsH** | | | | |
| --- | --- | --- | --- | --- |
| No known mutations found in 16S_rrsH |  |  |  |  |

| **rpoB** | | | | |
| --- | --- | --- | --- | --- |
| No mutations found in rpoB |  |  |  |  |

| **pmrB** | | | | |
| --- | --- | --- | --- | --- |
| No mutations found in pmrB |  |  |  |  |

| **parC** | | | | |
| --- | --- | --- | --- | --- |
| **Mutation** | **Nucleotide change** | **Amino acid change** | **Resistance** | **PMID** |
| parC p.A56T | GCC ➝ ACC | A ➝ T | Nalidixic acid,Ciprofloxacin | [12654733](http://www.ncbi.nlm.nih.gov/pubmed/12654733) |
| parC p.S80I | AGC ➝ ATC | S ➝ I | Nalidixic acid,Ciprofloxacin | [8851598](http://www.ncbi.nlm.nih.gov/pubmed/8851598) |

| **16S_rrsC** | | | | |
| --- | --- | --- | --- | --- |
| No known mutations found in 16S_rrsC |  |  |  |  |

| **16S_rrsB** | | | | |
| --- | --- | --- | --- | --- |
| No mutations found in 16S_rrsB |  |  |  |  |

| **ampC** | | | | |
| --- | --- | --- | --- | --- |
| No mutations found in ampC |  |  |  |  |

| **gyrA** | | | | |
| --- | --- | --- | --- | --- |
| **Mutation** | **Nucleotide change** | **Amino acid change** | **Resistance** | **PMID** |
| gyrA p.S83L | TCG ➝ TTG | S ➝ L | Nalidixic acid,Ciprofloxacin | [8891148](http://www.ncbi.nlm.nih.gov/pubmed/8891148) |
| gyrA p.D87N | GAC ➝ AAC | D ➝ N | Nalidixic acid,Ciprofloxacin | [12654733](http://www.ncbi.nlm.nih.gov/pubmed/12654733) |

| **23S** | | | | |
| --- | --- | --- | --- | --- |
| No known mutations found in 23S |  |  |  |  |

| **parE** | | | | |
| --- | --- | --- | --- | --- |
| No mutations found in parE |  |  |  |  |

| **gyrB** | | | | |
| --- | --- | --- | --- | --- |
| No known mutations found in gyrB |  |  |  |  |

Top of Form

Bottom of Form

Top of Form

Bottom of Form

**CITATIONS**

For publication of results, please cite:

- Identification of acquired antimicrobial resistance genes.
  Zankari E, Hasman H, Cosentino S, Vestergaard M, Rasmussen S, Lund O, Aarestrup FM, Larsen MV.
  J Antimicrob Chemother. 2012 Jul 10.
  PMID: [22782487](http://www.ncbi.nlm.nih.gov/pubmed/22782487)         doi: [10.1093/jac/dks261](http://dx.doi.org/10.1093/jac/dks261)


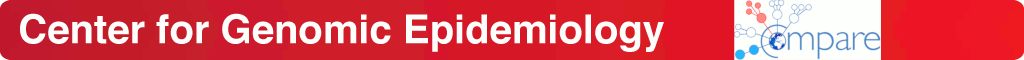


|  | Home | Services | Instructions | Output | Overview of genes | Article abstract |  |
| --- | --- | --- | --- | --- | --- | --- | --- |

**ResFinder-3.2 Server - Results**

**Input Files: *E009.fasta***

Show Acquired antimicrobial resistance results

**Acquired antimicrobial resistance gene - Results**

| **Aminoglycoside** | | | | | | |
| --- | --- | --- | --- | --- | --- | --- |
| **Resistance gene** | **Identity** | **Query / Template length** | **Contig** | **Position in contig** | **Predicted phenotype** | **Accession number** |
| aac(3)-IIa | 99.12 | 794 / 861 | NXLH01000079.1 Escherichia coli strain ST-131:E009 NODE_79_length_2003_cov_64.7426_ID_157, whole genome shotgun sequence | 1..794 | Aminoglycoside resistance | [L22613](http://www.ncbi.nlm.nih.gov/nuccore/L22613) |
| aac(3)-IId | 99.12 | 794 / 861 | NXLH01000079.1 Escherichia coli strain ST-131:E009 NODE_79_length_2003_cov_64.7426_ID_157, whole genome shotgun sequence | 1..794 | Aminoglycoside resistance | [EU022314](http://www.ncbi.nlm.nih.gov/nuccore/EU022314) |
| aac(6')-Ib-cr | 100 | 600 / 600 | NXLH01000074.1 Escherichia coli strain ST-131:E009 NODE_74_length_2383_cov_57.8052_ID_147, whole genome shotgun sequence | 1639..2238 | Fluoroquinolone and aminoglycoside resistance | [DQ303918](http://www.ncbi.nlm.nih.gov/nuccore/DQ303918) |
| aadA5 | 100 | 789 / 789 | NXLH01000070.1 Escherichia coli strain ST-131:E009 NODE_70_length_2653_cov_37.9824_ID_139, whole genome shotgun sequence | 263..1051 | Aminoglycoside resistance | [AF137361](http://www.ncbi.nlm.nih.gov/nuccore/AF137361) |
| aph(3'')-Ib | 100 | 804 / 804 | NXLH01000052.1 Escherichia coli strain ST-131:E009 NODE_52_length_7789_cov_17.7293_ID_103, whole genome shotgun sequence | 1456..2259 | Aminoglycoside resistance Alternate name; aph(3'')-Ib | [AF321551](http://www.ncbi.nlm.nih.gov/nuccore/AF321551) |
| aph(6)-Id | 100 | 831 / 831 | NXLH01000052.1 Escherichia coli strain ST-131:E009 NODE_52_length_7789_cov_17.7293_ID_103, whole genome shotgun sequence | 2265..3095 | Aminoglycoside resistance Alternate name; aph(6)-Id | [CP000971](http://www.ncbi.nlm.nih.gov/nuccore/CP000971) |

| **Colistin** | | | | | | |
| --- | --- | --- | --- | --- | --- | --- |
| **Resistance gene** | **Identity** | **Query / Template length** | **Contig** | **Position in contig** | **Predicted phenotype** | **Accession number** |
| No hit found | | | | | | |

| **Nitroimidazole** | | | | | | |
| --- | --- | --- | --- | --- | --- | --- |
| **Resistance gene** | **Identity** | **Query / Template length** | **Contig** | **Position in contig** | **Predicted phenotype** | **Accession number** |
| No hit found | | | | | | |

| **Glycopeptide** | | | | | | |
| --- | --- | --- | --- | --- | --- | --- |
| **Resistance gene** | **Identity** | **Query / Template length** | **Contig** | **Position in contig** | **Predicted phenotype** | **Accession number** |
| No hit found | | | | | | |

| **Sulphonamide** | | | | | | |
| --- | --- | --- | --- | --- | --- | --- |
| **Resistance gene** | **Identity** | **Query / Template length** | **Contig** | **Position in contig** | **Predicted phenotype** | **Accession number** |
| sul1 | 100 | 840 / 840 | NXLH01000057.1 Escherichia coli strain ST-131:E009 NODE_57_length_6124_cov_27.371_ID_113, whole genome shotgun sequence | 474..1313 | Sulphonamide resistance | [U12338](http://www.ncbi.nlm.nih.gov/nuccore/U12338) |
| sul2 | 99.88 | 816 / 816 | NXLH01000052.1 Escherichia coli strain ST-131:E009 NODE_52_length_7789_cov_17.7293_ID_103, whole genome shotgun sequence | 581..1395 | Sulphonamide resistance | [AY034138](http://www.ncbi.nlm.nih.gov/nuccore/AY034138) |

| **Rifampicin** | | | | | | |
| --- | --- | --- | --- | --- | --- | --- |
| **Resistance gene** | **Identity** | **Query / Template length** | **Contig** | **Position in contig** | **Predicted phenotype** | **Accession number** |
| No hit found | | | | | | |

| **Phenicol** | | | | | | |
| --- | --- | --- | --- | --- | --- | --- |
| **Resistance gene** | **Identity** | **Query / Template length** | **Contig** | **Position in contig** | **Predicted phenotype** | **Accession number** |
| catB3 | 100 | 442 / 633 | NXLH01000074.1 Escherichia coli strain ST-131:E009 NODE_74_length_2383_cov_57.8052_ID_147, whole genome shotgun sequence | 99..540 | Phenicol resistance | [AJ009818](http://www.ncbi.nlm.nih.gov/nuccore/AJ009818) |
| catB3 | 100 | 442 / 633 | NXLH01000074.1 Escherichia coli strain ST-131:E009 NODE_74_length_2383_cov_57.8052_ID_147, whole genome shotgun sequence | 99..540 | Phenicol resistance | [U13880](http://www.ncbi.nlm.nih.gov/nuccore/U13880) |

| **Fusidicacid** | | | | | | |
| --- | --- | --- | --- | --- | --- | --- |
| **Resistance gene** | **Identity** | **Query / Template length** | **Contig** | **Position in contig** | **Predicted phenotype** | **Accession number** |
| No hit found | | | | | | |

| **Tetracycline** | | | | | | |
| --- | --- | --- | --- | --- | --- | --- |
| **Resistance gene** | **Identity** | **Query / Template length** | **Contig** | **Position in contig** | **Predicted phenotype** | **Accession number** |
| tet(A) | 99.92 | 1200 / 1200 | NXLH01000052.1 Escherichia coli strain ST-131:E009 NODE_52_length_7789_cov_17.7293_ID_103, whole genome shotgun sequence | 4147..5346 | Tetracycline resistance | [AJ517790](http://www.ncbi.nlm.nih.gov/nuccore/AJ517790) |

| **Oxazolidinone** | | | | | | |
| --- | --- | --- | --- | --- | --- | --- |
| **Resistance gene** | **Identity** | **Query / Template length** | **Contig** | **Position in contig** | **Predicted phenotype** | **Accession number** |
| No hit found | | | | | | |

| **Beta-lactam** | | | | | | |
| --- | --- | --- | --- | --- | --- | --- |
| **Resistance gene** | **Identity** | **Query / Template length** | **Contig** | **Position in contig** | **Predicted phenotype** | **Accession number** |
| blaCTX-M-15 | 100 | 876 / 876 | NXLH01000068.1 Escherichia coli strain ST-131:E009 NODE_68_length_2972_cov_109.368_ID_135, whole genome shotgun sequence | 1706..2581 | Beta-lactam resistance Alternate name; UOE-1 | [AY044436](http://www.ncbi.nlm.nih.gov/nuccore/AY044436) |
| blaOXA-1 | 100 | 831 / 831 | NXLH01000074.1 Escherichia coli strain ST-131:E009 NODE_74_length_2383_cov_57.8052_ID_147, whole genome shotgun sequence | 678..1508 | Beta-lactam resistance | [HQ170510](http://www.ncbi.nlm.nih.gov/nuccore/HQ170510) |
| blaTEM-1B | 100 | 861 / 861 | NXLH01000051.1 Escherichia coli strain ST-131:E009 NODE_51_length_8371_cov_78.5783_ID_101, whole genome shotgun sequence | 236..1096 | Beta-lactam resistance Alternate name; RblaTEM-1 | [AY458016](http://www.ncbi.nlm.nih.gov/nuccore/AY458016) |

| **Macrolide** | | | | | | |
| --- | --- | --- | --- | --- | --- | --- |
| **Resistance gene** | **Identity** | **Query / Template length** | **Contig** | **Position in contig** | **Predicted phenotype** | **Accession number** |
| mdf(A) | 97.81 | 1233 / 1233 | NXLH01000001.1 Escherichia coli strain ST-131:E009 NODE_1_length_609707_cov_28.8805_ID_1, whole genome shotgun sequence | 422292..423524 | Warning: gene is missing from Notes file. Please inform curator. | [Y08743](http://www.ncbi.nlm.nih.gov/nuccore/Y08743) |

| **Trimethoprim** | | | | | | |
| --- | --- | --- | --- | --- | --- | --- |
| **Resistance gene** | **Identity** | **Query / Template length** | **Contig** | **Position in contig** | **Predicted phenotype** | **Accession number** |
| dfrA17 | 100 | 474 / 474 | NXLH01000070.1 Escherichia coli strain ST-131:E009 NODE_70_length_2653_cov_37.9824_ID_139, whole genome shotgun sequence | 1182..1655 | Trimethoprim resistance | [FJ460238](http://www.ncbi.nlm.nih.gov/nuccore/FJ460238) |

| **Quinolone** | | | | | | |
| --- | --- | --- | --- | --- | --- | --- |
| **Resistance gene** | **Identity** | **Query / Template length** | **Contig** | **Position in contig** | **Predicted phenotype** | **Accession number** |
| aac(6')-Ib-cr | 100 | 600 / 600 | NXLH01000074.1 Escherichia coli strain ST-131:E009 NODE_74_length_2383_cov_57.8052_ID_147, whole genome shotgun sequence | 1639..2238 | Fluoroquinolone and aminoglycoside resistance | [DQ303918](http://www.ncbi.nlm.nih.gov/nuccore/DQ303918) |

| **Fosfomycin** | | | | | | |
| --- | --- | --- | --- | --- | --- | --- |
| **Resistance gene** | **Identity** | **Query / Template length** | **Contig** | **Position in contig** | **Predicted phenotype** | **Accession number** |
| No hit found | | | | | | |

extended output

**Selected %ID threshold:  *90 %***

**Selected minimum length:  *60 %***

Top of Form

Bottom of Form

Top of Form

Bottom of Form

Top of Form

Bottom of Form

Top of Form

Bottom of Form

Show Point mutation results

**Chromosomal point mutations - Results**

**Species:*escherichia_coli***

**Known Mutations**

| **16S_rrsC** | | | | |
| --- | --- | --- | --- | --- |
| No known mutations found in 16S_rrsC |  |  |  |  |

| **pmrB** | | | | |
| --- | --- | --- | --- | --- |
| No known mutations found in pmrB |  |  |  |  |

| **parC** | | | | |
| --- | --- | --- | --- | --- |
| **Mutation** | **Nucleotide change** | **Amino acid change** | **Resistance** | **PMID** |
| parC p.S80I | AGC ➝ ATT | S ➝ I | Nalidixic acid,Ciprofloxacin | [8851598](http://www.ncbi.nlm.nih.gov/pubmed/8851598) |
| parC p.E84V | GAA ➝ GTA | E ➝ V | Nalidixic acid,Ciprofloxacin | [12654733](http://www.ncbi.nlm.nih.gov/pubmed/12654733) |

| **gyrB** | | | | |
| --- | --- | --- | --- | --- |
| No known mutations found in gyrB |  |  |  |  |

| **pmrA** | | | | |
| --- | --- | --- | --- | --- |
| No known mutations found in pmrA |  |  |  |  |

| **parE** | | | | |
| --- | --- | --- | --- | --- |
| **Mutation** | **Nucleotide change** | **Amino acid change** | **Resistance** | **PMID** |
| parE p.I529L | ATT ➝ CTT | I ➝ L | Nalidixic acid,Ciprofloxacin | [14506034](http://www.ncbi.nlm.nih.gov/pubmed/14506034) |

| **gyrA** | | | | |
| --- | --- | --- | --- | --- |
| **Mutation** | **Nucleotide change** | **Amino acid change** | **Resistance** | **PMID** |
| gyrA p.S83L | TCG ➝ TTG | S ➝ L | Nalidixic acid,Ciprofloxacin | [8891148](http://www.ncbi.nlm.nih.gov/pubmed/8891148) |
| gyrA p.D87N | GAC ➝ AAC | D ➝ N | Nalidixic acid,Ciprofloxacin | [12654733](http://www.ncbi.nlm.nih.gov/pubmed/12654733) |

| **folP** | | | | |
| --- | --- | --- | --- | --- |
| No known mutations found in folP |  |  |  |  |

| **23S** | | | | |
| --- | --- | --- | --- | --- |
| No known mutations found in 23S |  |  |  |  |

| **rpoB** | | | | |
| --- | --- | --- | --- | --- |
| No known mutations found in rpoB |  |  |  |  |

| **16S_rrsH** | | | | |
| --- | --- | --- | --- | --- |
| No known mutations found in 16S_rrsH |  |  |  |  |

| **ampC** | | | | |
| --- | --- | --- | --- | --- |
| No known mutations found in ampC |  |  |  |  |

| **16S_rrsB** | | | | |
| --- | --- | --- | --- | --- |
| No known mutations found in 16S_rrsB |  |  |  |  |

Top of Form

Bottom of Form

Top of Form

Bottom of Form

**CITATIONS**

For publication of results, please cite:

- Identification of acquired antimicrobial resistance genes.
  Zankari E, Hasman H, Cosentino S, Vestergaard M, Rasmussen S, Lund O, Aarestrup FM, Larsen MV.
  J Antimicrob Chemother. 2012 Jul 10.
  PMID: [22782487](http://www.ncbi.nlm.nih.gov/pubmed/22782487)         doi: [10.1093/jac/dks261](http://dx.doi.org/10.1093/jac/dks261)


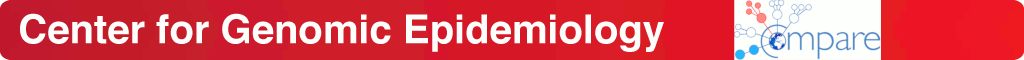


|  | Home | Services | Instructions | Output | Overview of genes | Article abstract |  |
| --- | --- | --- | --- | --- | --- | --- | --- |

**ResFinder-3.2 Server - Results**

**Input Files: *E011.fasta***

Show Acquired antimicrobial resistance results

**Acquired antimicrobial resistance gene - Results**

| **Nitroimidazole** | | | | | | |
| --- | --- | --- | --- | --- | --- | --- |
| **Resistance gene** | **Identity** | **Query / Template length** | **Contig** | **Position in contig** | **Predicted phenotype** | **Accession number** |
| No hit found | | | | | | |

| **Fusidicacid** | | | | | | |
| --- | --- | --- | --- | --- | --- | --- |
| **Resistance gene** | **Identity** | **Query / Template length** | **Contig** | **Position in contig** | **Predicted phenotype** | **Accession number** |
| No hit found | | | | | | |

| **Rifampicin** | | | | | | |
| --- | --- | --- | --- | --- | --- | --- |
| **Resistance gene** | **Identity** | **Query / Template length** | **Contig** | **Position in contig** | **Predicted phenotype** | **Accession number** |
| No hit found | | | | | | |

| **Beta-lactam** | | | | | | |
| --- | --- | --- | --- | --- | --- | --- |
| **Resistance gene** | **Identity** | **Query / Template length** | **Contig** | **Position in contig** | **Predicted phenotype** | **Accession number** |
| blaCTX-M-15 | 100 | 876 / 876 | NXKR01000088.1 Escherichia coli strain ST-131:E011 NODE_88_length_3369_cov_145.789, whole genome shotgun sequence | 1894..2769 | Beta-lactam resistance Alternate name; UOE-1 | [AY044436](http://www.ncbi.nlm.nih.gov/nuccore/AY044436) |
| blaOXA-1 | 100 | 831 / 831 | NXKR01000103.1 Escherichia coli strain ST-131:E011 NODE_103_length_2484_cov_65.9737, whole genome shotgun sequence | 729..1559 | Beta-lactam resistance | [HQ170510](http://www.ncbi.nlm.nih.gov/nuccore/HQ170510) |

| **Glycopeptide** | | | | | | |
| --- | --- | --- | --- | --- | --- | --- |
| **Resistance gene** | **Identity** | **Query / Template length** | **Contig** | **Position in contig** | **Predicted phenotype** | **Accession number** |
| No hit found | | | | | | |

| **Trimethoprim** | | | | | | |
| --- | --- | --- | --- | --- | --- | --- |
| **Resistance gene** | **Identity** | **Query / Template length** | **Contig** | **Position in contig** | **Predicted phenotype** | **Accession number** |
| dfrA14 | 100 | 474 / 474 | NXKR01000046.1 Escherichia coli strain ST-131:E011 NODE_46_length_21384_cov_30.9932, whole genome shotgun sequence | 10903..11376 | Trimethoprim resistance | [AF393510](http://www.ncbi.nlm.nih.gov/nuccore/AF393510) |
| dfrA17 | 100 | 474 / 474 | NXKR01000063.1 Escherichia coli strain ST-131:E011 NODE_63_length_10755_cov_77.7079, whole genome shotgun sequence | 1543..2016 | Trimethoprim resistance | [FJ460238](http://www.ncbi.nlm.nih.gov/nuccore/FJ460238) |

| **Aminoglycoside** | | | | | | |
| --- | --- | --- | --- | --- | --- | --- |
| **Resistance gene** | **Identity** | **Query / Template length** | **Contig** | **Position in contig** | **Predicted phenotype** | **Accession number** |
| aac(3)-IIa | 99.77 | 861 / 861 | NXKR01000096.1 Escherichia coli strain ST-131:E011 NODE_96_length_2804_cov_41.0062, whole genome shotgun sequence | 99..959 | Aminoglycoside resistance | [X51534](http://www.ncbi.nlm.nih.gov/nuccore/X51534) |
| aac(6')-Ib-cr | 100 | 600 / 600 | NXKR01000103.1 Escherichia coli strain ST-131:E011 NODE_103_length_2484_cov_65.9737, whole genome shotgun sequence | 1690..2289 | Fluoroquinolone and aminoglycoside resistance | [DQ303918](http://www.ncbi.nlm.nih.gov/nuccore/DQ303918) |
| aadA5 | 100 | 789 / 789 | NXKR01000063.1 Escherichia coli strain ST-131:E011 NODE_63_length_10755_cov_77.7079, whole genome shotgun sequence | 2147..2935 | Aminoglycoside resistance | [AF137361](http://www.ncbi.nlm.nih.gov/nuccore/AF137361) |
| aph(3'')-Ib | 99.81 | 529 / 804 | NXKR01000046.1 Escherichia coli strain ST-131:E011 NODE_46_length_21384_cov_30.9932, whole genome shotgun sequence | 11396..11923 | Aminoglycoside resistance Alternate name; aph(3'')-Ib | [AF321551](http://www.ncbi.nlm.nih.gov/nuccore/AF321551) |
| aph(6)-Id | 100 | 837 / 837 | NXKR01000046.1 Escherichia coli strain ST-131:E011 NODE_46_length_21384_cov_30.9932, whole genome shotgun sequence | 9717..10553 | Aminoglycoside resistance Alternate name; aph(6)-Id | [M28829](http://www.ncbi.nlm.nih.gov/nuccore/M28829) |

| **Macrolide** | | | | | | |
| --- | --- | --- | --- | --- | --- | --- |
| **Resistance gene** | **Identity** | **Query / Template length** | **Contig** | **Position in contig** | **Predicted phenotype** | **Accession number** |
| mdf(A) | 97.81 | 1233 / 1233 | NXKR01000012.1 Escherichia coli strain ST-131:E011 NODE_12_length_165049_cov_25.0754, whole genome shotgun sequence | 64137..65369 | Warning: gene is missing from Notes file. Please inform curator. | [Y08743](http://www.ncbi.nlm.nih.gov/nuccore/Y08743) |
| mph(A) | 100 | 906 / 906 | NXKR01000063.1 Escherichia coli strain ST-131:E011 NODE_63_length_10755_cov_77.7079, whole genome shotgun sequence | 9632..10537 | Macrolide resistance | [D16251](http://www.ncbi.nlm.nih.gov/nuccore/D16251) |

| **Tetracycline** | | | | | | |
| --- | --- | --- | --- | --- | --- | --- |
| **Resistance gene** | **Identity** | **Query / Template length** | **Contig** | **Position in contig** | **Predicted phenotype** | **Accession number** |
| No hit found | | | | | | |

| **Quinolone** | | | | | | |
| --- | --- | --- | --- | --- | --- | --- |
| **Resistance gene** | **Identity** | **Query / Template length** | **Contig** | **Position in contig** | **Predicted phenotype** | **Accession number** |
| aac(6')-Ib-cr | 100 | 600 / 600 | NXKR01000103.1 Escherichia coli strain ST-131:E011 NODE_103_length_2484_cov_65.9737, whole genome shotgun sequence | 1690..2289 | Fluoroquinolone and aminoglycoside resistance | [DQ303918](http://www.ncbi.nlm.nih.gov/nuccore/DQ303918) |

| **Phenicol** | | | | | | |
| --- | --- | --- | --- | --- | --- | --- |
| **Resistance gene** | **Identity** | **Query / Template length** | **Contig** | **Position in contig** | **Predicted phenotype** | **Accession number** |
| catB3 | 100 | 442 / 633 | NXKR01000103.1 Escherichia coli strain ST-131:E011 NODE_103_length_2484_cov_65.9737, whole genome shotgun sequence | 150..591 | Phenicol resistance | [AJ009818](http://www.ncbi.nlm.nih.gov/nuccore/AJ009818) |
| catB3 | 100 | 442 / 633 | NXKR01000103.1 Escherichia coli strain ST-131:E011 NODE_103_length_2484_cov_65.9737, whole genome shotgun sequence | 150..591 | Phenicol resistance | [U13880](http://www.ncbi.nlm.nih.gov/nuccore/U13880) |

| **Sulphonamide** | | | | | | |
| --- | --- | --- | --- | --- | --- | --- |
| **Resistance gene** | **Identity** | **Query / Template length** | **Contig** | **Position in contig** | **Predicted phenotype** | **Accession number** |
| sul1 | 99.88 | 840 / 840 | NXKR01000063.1 Escherichia coli strain ST-131:E011 NODE_63_length_10755_cov_77.7079, whole genome shotgun sequence | 3482..4321 | Sulphonamide resistance | [U12338](http://www.ncbi.nlm.nih.gov/nuccore/U12338) |
| sul2 | 100 | 816 / 816 | NXKR01000046.1 Escherichia coli strain ST-131:E011 NODE_46_length_21384_cov_30.9932, whole genome shotgun sequence | 11984..12799 | Sulphonamide resistance | [AY034138](http://www.ncbi.nlm.nih.gov/nuccore/AY034138) |

| **Colistin** | | | | | | |
| --- | --- | --- | --- | --- | --- | --- |
| **Resistance gene** | **Identity** | **Query / Template length** | **Contig** | **Position in contig** | **Predicted phenotype** | **Accession number** |
| No hit found | | | | | | |

| **Oxazolidinone** | | | | | | |
| --- | --- | --- | --- | --- | --- | --- |
| **Resistance gene** | **Identity** | **Query / Template length** | **Contig** | **Position in contig** | **Predicted phenotype** | **Accession number** |
| No hit found | | | | | | |

| **Fosfomycin** | | | | | | |
| --- | --- | --- | --- | --- | --- | --- |
| **Resistance gene** | **Identity** | **Query / Template length** | **Contig** | **Position in contig** | **Predicted phenotype** | **Accession number** |
| No hit found | | | | | | |

extended output

**Selected %ID threshold:  *90 %***

**Selected minimum length:  *60 %***

Top of Form

Bottom of Form

Top of Form

Bottom of Form

Top of Form

Bottom of Form

Top of Form

Bottom of Form

Show Point mutation results

**Chromosomal point mutations - Results**

**Species:*escherichia_coli***

**Known Mutations**

| **16S_rrsH** | | | | |
| --- | --- | --- | --- | --- |
| No known mutations found in 16S_rrsH |  |  |  |  |

| **folP** | | | | |
| --- | --- | --- | --- | --- |
| No known mutations found in folP |  |  |  |  |

| **pmrB** | | | | |
| --- | --- | --- | --- | --- |
| No known mutations found in pmrB |  |  |  |  |

| **rpoB** | | | | |
| --- | --- | --- | --- | --- |
| No known mutations found in rpoB |  |  |  |  |

| **parE** | | | | |
| --- | --- | --- | --- | --- |
| **Mutation** | **Nucleotide change** | **Amino acid change** | **Resistance** | **PMID** |
| parE p.I529L | ATT ➝ CTT | I ➝ L | Nalidixic acid,Ciprofloxacin | [14506034](http://www.ncbi.nlm.nih.gov/pubmed/14506034) |

| **gyrA** | | | | |
| --- | --- | --- | --- | --- |
| **Mutation** | **Nucleotide change** | **Amino acid change** | **Resistance** | **PMID** |
| gyrA p.S83L | TCG ➝ TTG | S ➝ L | Nalidixic acid,Ciprofloxacin | [8891148](http://www.ncbi.nlm.nih.gov/pubmed/8891148) |
| gyrA p.D87N | GAC ➝ AAC | D ➝ N | Nalidixic acid,Ciprofloxacin | [12654733](http://www.ncbi.nlm.nih.gov/pubmed/12654733) |

| **parC** | | | | |
| --- | --- | --- | --- | --- |
| **Mutation** | **Nucleotide change** | **Amino acid change** | **Resistance** | **PMID** |
| parC p.S80I | AGC ➝ ATT | S ➝ I | Nalidixic acid,Ciprofloxacin | [8851598](http://www.ncbi.nlm.nih.gov/pubmed/8851598) |
| parC p.E84V | GAA ➝ GTA | E ➝ V | Nalidixic acid,Ciprofloxacin | [12654733](http://www.ncbi.nlm.nih.gov/pubmed/12654733) |

| **pmrA** | | | | |
| --- | --- | --- | --- | --- |
| No known mutations found in pmrA |  |  |  |  |

| **ampC** | | | | |
| --- | --- | --- | --- | --- |
| No known mutations found in ampC |  |  |  |  |

| **16S_rrsC** | | | | |
| --- | --- | --- | --- | --- |
| No known mutations found in 16S_rrsC |  |  |  |  |

| **gyrB** | | | | |
| --- | --- | --- | --- | --- |
| No known mutations found in gyrB |  |  |  |  |

| **23S** | | | | |
| --- | --- | --- | --- | --- |
| No known mutations found in 23S |  |  |  |  |

| **16S_rrsB** | | | | |
| --- | --- | --- | --- | --- |
| No known mutations found in 16S_rrsB |  |  |  |  |

Top of Form

Bottom of Form

Top of Form

Bottom of Form

**CITATIONS**

For publication of results, please cite:

- Identification of acquired antimicrobial resistance genes.
  Zankari E, Hasman H, Cosentino S, Vestergaard M, Rasmussen S, Lund O, Aarestrup FM, Larsen MV.
  J Antimicrob Chemother. 2012 Jul 10.
  PMID: [22782487](http://www.ncbi.nlm.nih.gov/pubmed/22782487)         doi: [10.1093/jac/dks261](http://dx.doi.org/10.1093/jac/dks261)


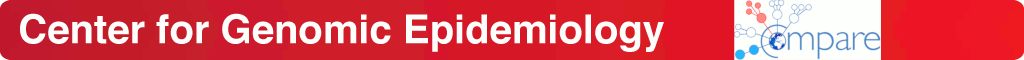


|  | Home | Services | Instructions | Output | Overview of genes | Article abstract |  |
| --- | --- | --- | --- | --- | --- | --- | --- |

**ResFinder-3.2 Server - Results**

**Input Files: *E019.fasta***

Show Acquired antimicrobial resistance results

**Acquired antimicrobial resistance gene - Results**

| **Trimethoprim** | | | | | | |
| --- | --- | --- | --- | --- | --- | --- |
| **Resistance gene** | **Identity** | **Query / Template length** | **Contig** | **Position in contig** | **Predicted phenotype** | **Accession number** |
| dfrA17 | 100 | 474 / 474 | NXLG01000064.1 Escherichia coli strain ST-617:E019 NODE_64_length_10136_cov_53.4991_ID_127, whole genome shotgun sequence | 975..1448 | Trimethoprim resistance | [FJ460238](http://www.ncbi.nlm.nih.gov/nuccore/FJ460238) |

| **Sulphonamide** | | | | | | |
| --- | --- | --- | --- | --- | --- | --- |
| **Resistance gene** | **Identity** | **Query / Template length** | **Contig** | **Position in contig** | **Predicted phenotype** | **Accession number** |
| sul1 | 100 | 840 / 840 | NXLG01000064.1 Escherichia coli strain ST-617:E019 NODE_64_length_10136_cov_53.4991_ID_127, whole genome shotgun sequence | 2914..3753 | Sulphonamide resistance | [U12338](http://www.ncbi.nlm.nih.gov/nuccore/U12338) |
| sul2 | 100 | 816 / 816 | NXLG01000074.1 Escherichia coli strain ST-617:E019 NODE_74_length_6277_cov_243.491_ID_147, whole genome shotgun sequence | 4027..4842 | Sulphonamide resistance | [AY034138](http://www.ncbi.nlm.nih.gov/nuccore/AY034138) |

| **Aminoglycoside** | | | | | | |
| --- | --- | --- | --- | --- | --- | --- |
| **Resistance gene** | **Identity** | **Query / Template length** | **Contig** | **Position in contig** | **Predicted phenotype** | **Accession number** |
| aac(3)-IId | 99.88 | 861 / 861 | NXLG01000087.1 Escherichia coli strain ST-617:E019 NODE_87_length_3875_cov_69.9187_ID_173, whole genome shotgun sequence | 2730..3590 | Aminoglycoside resistance | [EU022314](http://www.ncbi.nlm.nih.gov/nuccore/EU022314) |
| aac(6')-Ib-cr | 100 | 600 / 600 | NXLG01000097.1 Escherichia coli strain ST-617:E019 NODE_97_length_2384_cov_121.065_ID_193, whole genome shotgun sequence | 146..745 | Fluoroquinolone and aminoglycoside resistance | [DQ303918](http://www.ncbi.nlm.nih.gov/nuccore/DQ303918) |
| aadA5 | 100 | 789 / 789 | NXLG01000064.1 Escherichia coli strain ST-617:E019 NODE_64_length_10136_cov_53.4991_ID_127, whole genome shotgun sequence | 1579..2367 | Aminoglycoside resistance | [AF137361](http://www.ncbi.nlm.nih.gov/nuccore/AF137361) |
| aph(3'')-Ib | 100 | 801 / 804 | NXLG01000074.1 Escherichia coli strain ST-617:E019 NODE_74_length_6277_cov_243.491_ID_147, whole genome shotgun sequence | 4906..5706 | Aminoglycoside resistance Alternate name; aph(3'')-Ib | [AF321551](http://www.ncbi.nlm.nih.gov/nuccore/AF321551) |
| aph(6)-Id | 100 | 572 / 837 | NXLG01000074.1 Escherichia coli strain ST-617:E019 NODE_74_length_6277_cov_243.491_ID_147, whole genome shotgun sequence | 5706..6277 | Aminoglycoside resistance Alternate name; aph(6)-Id | [18676889](http://www.ncbi.nlm.nih.gov/nuccore/18676889) |
| aph(6)-Id | 100 | 572 / 837 | NXLG01000074.1 Escherichia coli strain ST-617:E019 NODE_74_length_6277_cov_243.491_ID_147, whole genome shotgun sequence | 5706..6277 | Aminoglycoside resistance Alternate name; aph(6)-Id | [AF024602](http://www.ncbi.nlm.nih.gov/nuccore/AF024602) |
| aph(6)-Id | 100 | 572 / 837 | NXLG01000074.1 Escherichia coli strain ST-617:E019 NODE_74_length_6277_cov_243.491_ID_147, whole genome shotgun sequence | 5706..6277 | Aminoglycoside resistance Alternate name; aph(6)-Id | [M28829](http://www.ncbi.nlm.nih.gov/nuccore/M28829) |

| **Glycopeptide** | | | | | | |
| --- | --- | --- | --- | --- | --- | --- |
| **Resistance gene** | **Identity** | **Query / Template length** | **Contig** | **Position in contig** | **Predicted phenotype** | **Accession number** |
| No hit found | | | | | | |

| **Quinolone** | | | | | | |
| --- | --- | --- | --- | --- | --- | --- |
| **Resistance gene** | **Identity** | **Query / Template length** | **Contig** | **Position in contig** | **Predicted phenotype** | **Accession number** |
| aac(6')-Ib-cr | 100 | 600 / 600 | NXLG01000097.1 Escherichia coli strain ST-617:E019 NODE_97_length_2384_cov_121.065_ID_193, whole genome shotgun sequence | 146..745 | Fluoroquinolone and aminoglycoside resistance | [DQ303918](http://www.ncbi.nlm.nih.gov/nuccore/DQ303918) |

| **Fusidicacid** | | | | | | |
| --- | --- | --- | --- | --- | --- | --- |
| **Resistance gene** | **Identity** | **Query / Template length** | **Contig** | **Position in contig** | **Predicted phenotype** | **Accession number** |
| No hit found | | | | | | |

| **Tetracycline** | | | | | | |
| --- | --- | --- | --- | --- | --- | --- |
| **Resistance gene** | **Identity** | **Query / Template length** | **Contig** | **Position in contig** | **Predicted phenotype** | **Accession number** |
| tet(B) | 100 | 1206 / 1206 | NXLG01000077.1 Escherichia coli strain ST-617:E019 NODE_77_length_5283_cov_179.57_ID_153, whole genome shotgun sequence | 2453..3658 | Tetracycline resistance | [AF326777](http://www.ncbi.nlm.nih.gov/nuccore/AF326777) |

| **Nitroimidazole** | | | | | | |
| --- | --- | --- | --- | --- | --- | --- |
| **Resistance gene** | **Identity** | **Query / Template length** | **Contig** | **Position in contig** | **Predicted phenotype** | **Accession number** |
| No hit found | | | | | | |

| **Beta-lactam** | | | | | | |
| --- | --- | --- | --- | --- | --- | --- |
| **Resistance gene** | **Identity** | **Query / Template length** | **Contig** | **Position in contig** | **Predicted phenotype** | **Accession number** |
| blaCTX-M-15 | 100 | 876 / 876 | NXLG01000076.1 Escherichia coli strain ST-617:E019 NODE_76_length_5583_cov_98.5852_ID_151, whole genome shotgun sequence | 2737..3612 | Beta-lactam resistance Alternate name; UOE-1 | [AY044436](http://www.ncbi.nlm.nih.gov/nuccore/AY044436) |
| blaOXA-1 | 100 | 831 / 831 | NXLG01000097.1 Escherichia coli strain ST-617:E019 NODE_97_length_2384_cov_121.065_ID_193, whole genome shotgun sequence | 876..1706 | Beta-lactam resistance | [HQ170510](http://www.ncbi.nlm.nih.gov/nuccore/HQ170510) |

| **Macrolide** | | | | | | |
| --- | --- | --- | --- | --- | --- | --- |
| **Resistance gene** | **Identity** | **Query / Template length** | **Contig** | **Position in contig** | **Predicted phenotype** | **Accession number** |
| mdf(A) | 99.92 | 1233 / 1233 | NXLG01000003.1 Escherichia coli strain ST-617:E019 NODE_3_length_235313_cov_66.8949_ID_5, whole genome shotgun sequence | 145428..146660 | Warning: gene is missing from Notes file. Please inform curator. | [Y08743](http://www.ncbi.nlm.nih.gov/nuccore/Y08743) |
| mph(A) | 100 | 906 / 906 | NXLG01000064.1 Escherichia coli strain ST-617:E019 NODE_64_length_10136_cov_53.4991_ID_127, whole genome shotgun sequence | 9063..9968 | Macrolide resistance | [D16251](http://www.ncbi.nlm.nih.gov/nuccore/D16251) |

| **Phenicol** | | | | | | |
| --- | --- | --- | --- | --- | --- | --- |
| **Resistance gene** | **Identity** | **Query / Template length** | **Contig** | **Position in contig** | **Predicted phenotype** | **Accession number** |
| catA1 | 99.75 | 407 / 660 | NXLG01000079.1 Escherichia coli strain ST-617:E019 NODE_79_length_4792_cov_68.2312_ID_157, whole genome shotgun sequence | 4260..4666 | Phenicol resistance | [V00622](http://www.ncbi.nlm.nih.gov/nuccore/V00622) |
| catB3 | 100 | 442 / 633 | NXLG01000097.1 Escherichia coli strain ST-617:E019 NODE_97_length_2384_cov_121.065_ID_193, whole genome shotgun sequence | 1844..2285 | Phenicol resistance | [AJ009818](http://www.ncbi.nlm.nih.gov/nuccore/AJ009818) |
| catB3 | 100 | 442 / 633 | NXLG01000097.1 Escherichia coli strain ST-617:E019 NODE_97_length_2384_cov_121.065_ID_193, whole genome shotgun sequence | 1844..2285 | Phenicol resistance | [U13880](http://www.ncbi.nlm.nih.gov/nuccore/U13880) |

extended output

cfdz

Show Point mutation results

**Chromosomal point mutations - Results**

**Species:*escherichia_coli***

**Known Mutations**

| **pmrB** | | | | |
| --- | --- | --- | --- | --- |
| No mutations found in pmrB |  |  |  |  |

| **16S_rrsB** | | | | |
| --- | --- | --- | --- | --- |
| No mutations found in 16S_rrsB |  |  |  |  |

| **16S_rrsC** | | | | |
| --- | --- | --- | --- | --- |
| No known mutations found in 16S_rrsC |  |  |  |  |

| **gyrA** | | | | |
| --- | --- | --- | --- | --- |
| **Mutation** | **Nucleotide change** | **Amino acid change** | **Resistance** | **PMID** |
| gyrA p.S83L | TCG ➝ TTG | S ➝ L | Nalidixic acid,Ciprofloxacin | [8891148](http://www.ncbi.nlm.nih.gov/pubmed/8891148) |
| gyrA p.D87N | GAC ➝ AAC | D ➝ N | Nalidixic acid,Ciprofloxacin | [12654733](http://www.ncbi.nlm.nih.gov/pubmed/12654733) |

| **parC** | | | | |
| --- | --- | --- | --- | --- |
| **Mutation** | **Nucleotide change** | **Amino acid change** | **Resistance** | **PMID** |
| parC p.S80I | AGC ➝ ATC | S ➝ I | Nalidixic acid,Ciprofloxacin | [8851598](http://www.ncbi.nlm.nih.gov/pubmed/8851598) |

| **23S** | | | | |
| --- | --- | --- | --- | --- |
| No known mutations found in 23S |  |  |  |  |

| **parE** | | | | |
| --- | --- | --- | --- | --- |
| **Mutation** | **Nucleotide change** | **Amino acid change** | **Resistance** | **PMID** |
| parE p.S458A | TCG ➝ GCG | S ➝ A | Nalidixic acid,Ciprofloxacin | [28598203](http://www.ncbi.nlm.nih.gov/pubmed/28598203) |

| **pmrA** | | | | |
| --- | --- | --- | --- | --- |
| No mutations found in pmrA |  |  |  |  |

| **gyrB** | | | | |
| --- | --- | --- | --- | --- |
| No mutations found in gyrB |  |  |  |  |

| **16S_rrsH** | | | | |
| --- | --- | --- | --- | --- |
| No known mutations found in 16S_rrsH |  |  |  |  |

| **rpoB** | | | | |
| --- | --- | --- | --- | --- |
| No known mutations found in rpoB |  |  |  |  |

| **ampC** | | | | |
| --- | --- | --- | --- | --- |
| No mutations found in ampC |  |  |  |  |

| **folP** | | | | |
| --- | --- | --- | --- | --- |
| No known mutations found in folP |  |  |  |  |

Top of Form

Bottom of Form

Top of Form

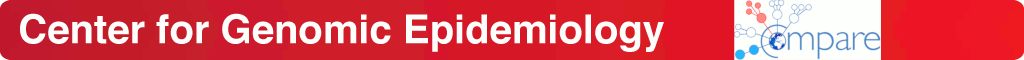


|  | Home | Services | Instructions | Output | Overview of genes | Article abstract |  |
| --- | --- | --- | --- | --- | --- | --- | --- |

**ResFinder-3.2 Server - Results**

**Input Files: *E020.fasta***

Show Acquired antimicrobial resistance results

**Acquired antimicrobial resistance gene - Results**

| **Phenicol** | | | | | | |
| --- | --- | --- | --- | --- | --- | --- |
| **Resistance gene** | **Identity** | **Query / Template length** | **Contig** | **Position in contig** | **Predicted phenotype** | **Accession number** |
| catA1 | 99.75 | 407 / 660 | NXJB01000089.1 Escherichia coli strain ST-617:E020 NODE_89_length_4719_cov_55.4455, whole genome shotgun sequence | 4260..4666 | Phenicol resistance | [V00622](http://www.ncbi.nlm.nih.gov/nuccore/V00622) |
| catB3 | 100 | 442 / 633 | NXJB01000105.1 Escherichia coli strain ST-617:E020 NODE_105_length_2484_cov_111.431, whole genome shotgun sequence | 150..591 | Phenicol resistance | [AJ009818](http://www.ncbi.nlm.nih.gov/nuccore/AJ009818) |
| catB3 | 100 | 442 / 633 | NXJB01000105.1 Escherichia coli strain ST-617:E020 NODE_105_length_2484_cov_111.431, whole genome shotgun sequence | 150..591 | Phenicol resistance | [U13880](http://www.ncbi.nlm.nih.gov/nuccore/U13880) |

| **Macrolide** | | | | | | |
| --- | --- | --- | --- | --- | --- | --- |
| **Resistance gene** | **Identity** | **Query / Template length** | **Contig** | **Position in contig** | **Predicted phenotype** | **Accession number** |
| mdf(A) | 99.92 | 1233 / 1233 | NXJB01000001.1 Escherichia coli strain ST-617:E020 NODE_1_length_354459_cov_47.0873, whole genome shotgun sequence | 208609..209841 | Warning: gene is missing from Notes file. Please inform curator. | [Y08743](http://www.ncbi.nlm.nih.gov/nuccore/Y08743) |
| mph(A) | 100 | 906 / 906 | NXJB01000069.1 Escherichia coli strain ST-617:E020 NODE_69_length_11231_cov_61.9453, whole genome shotgun sequence | 10108..11013 | Macrolide resistance | [D16251](http://www.ncbi.nlm.nih.gov/nuccore/D16251) |

| **Aminoglycoside** | | | | | | |
| --- | --- | --- | --- | --- | --- | --- |
| **Resistance gene** | **Identity** | **Query / Template length** | **Contig** | **Position in contig** | **Predicted phenotype** | **Accession number** |
| aac(3)-IId | 99.88 | 861 / 861 | NXJB01000097.1 Escherichia coli strain ST-617:E020 NODE_97_length_3830_cov_65.1862, whole genome shotgun sequence | 2729..3589 | Aminoglycoside resistance | [EU022314](http://www.ncbi.nlm.nih.gov/nuccore/EU022314) |
| aac(6')-Ib-cr | 100 | 600 / 600 | NXJB01000105.1 Escherichia coli strain ST-617:E020 NODE_105_length_2484_cov_111.431, whole genome shotgun sequence | 1690..2289 | Fluoroquinolone and aminoglycoside resistance | [DQ303918](http://www.ncbi.nlm.nih.gov/nuccore/DQ303918) |
| aadA5 | 100 | 789 / 789 | NXJB01000069.1 Escherichia coli strain ST-617:E020 NODE_69_length_11231_cov_61.9453, whole genome shotgun sequence | 2624..3412 | Aminoglycoside resistance | [AF137361](http://www.ncbi.nlm.nih.gov/nuccore/AF137361) |
| aph(3'')-Ib | 99.88 | 801 / 804 | NXJB01000082.1 Escherichia coli strain ST-617:E020 NODE_82_length_6231_cov_208.46, whole genome shotgun sequence | 2299..3098 | Aminoglycoside resistance Alternate name; aph(3'')-Ib | [AF321551](http://www.ncbi.nlm.nih.gov/nuccore/AF321551) |
| aph(6)-Id | 100 | 837 / 837 | NXJB01000082.1 Escherichia coli strain ST-617:E020 NODE_82_length_6231_cov_208.46, whole genome shotgun sequence | 3098..3934 | Aminoglycoside resistance Alternate name; aph(6)-Id | [M28829](http://www.ncbi.nlm.nih.gov/nuccore/M28829) |

| **Beta-lactam** | | | | | | |
| --- | --- | --- | --- | --- | --- | --- |
| **Resistance gene** | **Identity** | **Query / Template length** | **Contig** | **Position in contig** | **Predicted phenotype** | **Accession number** |
| blaCTX-M-15 | 100 | 876 / 876 | NXJB01000084.1 Escherichia coli strain ST-617:E020 NODE_84_length_5632_cov_81.2413, whole genome shotgun sequence | 2736..3611 | Beta-lactam resistance Alternate name; UOE-1 | [AY044436](http://www.ncbi.nlm.nih.gov/nuccore/AY044436) |
| blaOXA-1 | 100 | 831 / 831 | NXJB01000105.1 Escherichia coli strain ST-617:E020 NODE_105_length_2484_cov_111.431, whole genome shotgun sequence | 729..1559 | Beta-lactam resistance | [HQ170510](http://www.ncbi.nlm.nih.gov/nuccore/HQ170510) |
| blaTEM-1B | 100 | 861 / 861 | NXJB01000083.1 Escherichia coli strain ST-617:E020 NODE_83_length_5883_cov_100.728, whole genome shotgun sequence | 277..1137 | Beta-lactam resistance Alternate name; RblaTEM-1 | [AY458016](http://www.ncbi.nlm.nih.gov/nuccore/AY458016) |

| **Resistance gene** | **Identity** | **Query / Template length** | **Contig** | **Position in contig** | **Predicted phenotype** | **Accession number** |
| --- | --- | --- | --- | --- | --- | --- |
| sul1 | 100 | 840 / 840 | NXJB01000069.1 Escherichia coli strain ST-617:E020 NODE_69_length_11231_cov_61.9453, whole genome shotgun sequence | 3959..4798 | Sulphonamide resistance | [U12338](http://www.ncbi.nlm.nih.gov/nuccore/U12338) |
| sul2 | 100 | 816 / 816 | NXJB01000082.1 Escherichia coli strain ST-617:E020 NODE_82_length_6231_cov_208.46, whole genome shotgun sequence | 1420..2235 | Sulphonamide resistance | [AY034138](http://www.ncbi.nlm.nih.gov/nuccore/AY034138) |

| **Tetracycline** | | | | | | |
| --- | --- | --- | --- | --- | --- | --- |
| **Resistance gene** | **Identity** | **Query / Template length** | **Contig** | **Position in contig** | **Predicted phenotype** | **Accession number** |
| tet(B) | 100 | 1206 / 1206 | NXJB01000088.1 Escherichia coli strain ST-617:E020 NODE_88_length_5017_cov_114.011, whole genome shotgun sequence | 2231..3436 | Tetracycline resistance | [AF326777](http://www.ncbi.nlm.nih.gov/nuccore/AF326777) |

| **Nitroimidazole** | | | | | | |
| --- | --- | --- | --- | --- | --- | --- |
| **Resistance gene** | **Identity** | **Query / Template length** | **Contig** | **Position in contig** | **Predicted phenotype** | **Accession number** |
| No hit found | | | | | | |

| **Trimethoprim** | | | | | | |
| --- | --- | --- | --- | --- | --- | --- |
| **Resistance gene** | **Identity** | **Query / Template length** | **Contig** | **Position in contig** | **Predicted phenotype** | **Accession number** |
| dfrA17 | 100 | 474 / 474 | NXJB01000069.1 Escherichia coli strain ST-617:E020 NODE_69_length_11231_cov_61.9453, whole genome shotgun sequence | 2020..2493 | Trimethoprim resistance | [FJ460238](http://www.ncbi.nlm.nih.gov/nuccore/FJ460238) |

| **Quinolone** | | | | | | |
| --- | --- | --- | --- | --- | --- | --- |
| **Resistance gene** | **Identity** | **Query / Template length** | **Contig** | **Position in contig** | **Predicted phenotype** | **Accession number** |
| aac(6')-Ib-cr | 100 | 600 / 600 | NXJB01000105.1 Escherichia coli strain ST-617:E020 NODE_105_length_2484_cov_111.431, whole genome shotgun sequence | 1690..2289 | Fluoroquinolone and aminoglycoside resistance | [DQ303918](http://www.ncbi.nlm.nih.gov/nuccore/DQ303918) |

extended output

Show Point mutation results

**Chromosomal point mutations - Results**

**Species:*escherichia_coli***

**Known Mutations**

| **gyrA** | | | | |
| --- | --- | --- | --- | --- |
| **Mutation** | **Nucleotide change** | **Amino acid change** | **Resistance** | **PMID** |
| gyrA p.S83L | TCG ➝ TTG | S ➝ L | Nalidixic acid,Ciprofloxacin | [8891148](http://www.ncbi.nlm.nih.gov/pubmed/8891148) |
| gyrA p.D87N | GAC ➝ AAC | D ➝ N | Nalidixic acid,Ciprofloxacin | [12654733](http://www.ncbi.nlm.nih.gov/pubmed/12654733) |

| **23S** | | | | |
| --- | --- | --- | --- | --- |
| No known mutations found in 23S |  |  |  |  |

| **gyrB** | | | | |
| --- | --- | --- | --- | --- |
| No mutations found in gyrB |  |  |  |  |

| **parE** | | | | |
| --- | --- | --- | --- | --- |
| **Mutation** | **Nucleotide change** | **Amino acid change** | **Resistance** | **PMID** |
| parE p.S458A | TCG ➝ GCG | S ➝ A | Nalidixic acid,Ciprofloxacin | [28598203](http://www.ncbi.nlm.nih.gov/pubmed/28598203) |

| **16S_rrsC** | | | | |
| --- | --- | --- | --- | --- |
| No known mutations found in 16S_rrsC |  |  |  |  |

| **pmrA** | | | | |
| --- | --- | --- | --- | --- |
| No mutations found in pmrA |  |  |  |  |

| **parC** | | | | |
| --- | --- | --- | --- | --- |
| **Mutation** | **Nucleotide change** | **Amino acid change** | **Resistance** | **PMID** |
| parC p.S80I | AGC ➝ ATC | S ➝ I | Nalidixic acid,Ciprofloxacin | [8851598](http://www.ncbi.nlm.nih.gov/pubmed/8851598) |

| **16S_rrsB** | | | | |
| --- | --- | --- | --- | --- |
| No mutations found in 16S_rrsB |  |  |  |  |

| **16S_rrsH** | | | | |
| --- | --- | --- | --- | --- |
| No known mutations found in 16S_rrsH |  |  |  |  |

| **ampC** | | | | |
| --- | --- | --- | --- | --- |
| No mutations found in ampC |  |  |  |  |

| **folP** | | | | |
| --- | --- | --- | --- | --- |
| No known mutations found in folP |  |  |  |  |

| **rpoB** | | | | |
| --- | --- | --- | --- | --- |
| No mutations found in rpoB |  |  |  |  |

| **pmrB** | | | | |
| --- | --- | --- | --- | --- |
| No mutations found in pmrB |  |  |  |  |

Top of Form

Bottom of Form

Top of Form

Bottom of Form


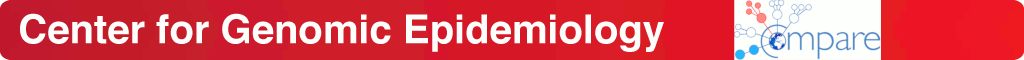


|  | Home | Services | Instructions | Output | Overview of genes | Article abstract |  |
| --- | --- | --- | --- | --- | --- | --- | --- |

**ResFinder-3.2 Server - Results**

**Input Files: *E021.fasta***

Show Acquired antimicrobial resistance results

**Acquired antimicrobial resistance gene - Results**

| **Phenicol** | | | | | | |
| --- | --- | --- | --- | --- | --- | --- |
| **Resistance gene** | **Identity** | **Query / Template length** | **Contig** | **Position in contig** | **Predicted phenotype** | **Accession number** |
| catB3 | 100 | 442 / 633 | NXIO01000090.1 Escherichia coli strain ST-131:E021 NODE_90_length_2384_cov_153.732_ID_179, whole genome shotgun sequence | 100..541 | Phenicol resistance | [AJ009818](http://www.ncbi.nlm.nih.gov/nuccore/AJ009818) |
| catB3 | 100 | 442 / 633 | NXIO01000090.1 Escherichia coli strain ST-131:E021 NODE_90_length_2384_cov_153.732_ID_179, whole genome shotgun sequence | 100..541 | Phenicol resistance | [U13880](http://www.ncbi.nlm.nih.gov/nuccore/U13880) |

| **Macrolide** | | | | | | |
| --- | --- | --- | --- | --- | --- | --- |
| **Resistance gene** | **Identity** | **Query / Template length** | **Contig** | **Position in contig** | **Predicted phenotype** | **Accession number** |
| mdf(A) | 97.81 | 1233 / 1233 | NXIO01000012.1 Escherichia coli strain ST-131:E021 NODE_12_length_166728_cov_101.803_ID_23, whole genome shotgun sequence | 99763..100995 | Warning: gene is missing from Notes file. Please inform curator. | [Y08743](http://www.ncbi.nlm.nih.gov/nuccore/Y08743) |
| mph(A) | 100 | 906 / 906 | NXIO01000056.1 Escherichia coli strain ST-131:E021 NODE_56_length_9965_cov_136.992_ID_111, whole genome shotgun sequence | 8893..9798 | Macrolide resistance | [D16251](http://www.ncbi.nlm.nih.gov/nuccore/D16251) |

| **Sulphonamide** | | | | | | |
| --- | --- | --- | --- | --- | --- | --- |
| **Resistance gene** | **Identity** | **Query / Template length** | **Contig** | **Position in contig** | **Predicted phenotype** | **Accession number** |
| sul1 | 100 | 840 / 840 | NXIO01000056.1 Escherichia coli strain ST-131:E021 NODE_56_length_9965_cov_136.992_ID_111, whole genome shotgun sequence | 2743..3582 | Sulphonamide resistance | [U12338](http://www.ncbi.nlm.nih.gov/nuccore/U12338) |
| sul2 | 100 | 816 / 816 | NXIO01000062.1 Escherichia coli strain ST-131:E021 NODE_62_length_6854_cov_124.933_ID_123, whole genome shotgun sequence | 580..1395 | Sulphonamide resistance | [AY034138](http://www.ncbi.nlm.nih.gov/nuccore/AY034138) |

| **Quinolone** | | | | | | |
| --- | --- | --- | --- | --- | --- | --- |
| **Resistance gene** | **Identity** | **Query / Template length** | **Contig** | **Position in contig** | **Predicted phenotype** | **Accession number** |
| aac(6')-Ib-cr | 100 | 600 / 600 | NXIO01000090.1 Escherichia coli strain ST-131:E021 NODE_90_length_2384_cov_153.732_ID_179, whole genome shotgun sequence | 1640..2239 | Fluoroquinolone and aminoglycoside resistance | [DQ303918](http://www.ncbi.nlm.nih.gov/nuccore/DQ303918) |

| **Tetracycline** | | | | | | |
| --- | --- | --- | --- | --- | --- | --- |
| **Resistance gene** | **Identity** | **Query / Template length** | **Contig** | **Position in contig** | **Predicted phenotype** | **Accession number** |
| tet(A) | 100 | 1200 / 1200 | NXIO01000062.1 Escherichia coli strain ST-131:E021 NODE_62_length_6854_cov_124.933_ID_123, whole genome shotgun sequence | 4149..5348 | Tetracycline resistance | [AJ517790](http://www.ncbi.nlm.nih.gov/nuccore/AJ517790) |

| **Trimethoprim** | | | | | | |
| --- | --- | --- | --- | --- | --- | --- |
| **Resistance gene** | **Identity** | **Query / Template length** | **Contig** | **Position in contig** | **Predicted phenotype** | **Accession number** |
| dfrA17 | 100 | 474 / 474 | NXIO01000056.1 Escherichia coli strain ST-131:E021 NODE_56_length_9965_cov_136.992_ID_111, whole genome shotgun sequence | 804..1277 | Trimethoprim resistance | [FJ460238](http://www.ncbi.nlm.nih.gov/nuccore/FJ460238) |

| **Beta-lactam** | | | | | | |
| --- | --- | --- | --- | --- | --- | --- |
| **Resistance gene** | **Identity** | **Query / Template length** | **Contig** | **Position in contig** | **Predicted phenotype** | **Accession number** |
| blaCTX-M-15 | 100 | 876 / 876 | NXIO01000082.1 Escherichia coli strain ST-131:E021 NODE_82_length_3051_cov_427.082_ID_163, whole genome shotgun sequence | 471..1346 | Beta-lactam resistance Alternate name; UOE-1 | [AY044436](http://www.ncbi.nlm.nih.gov/nuccore/AY044436) |
| blaOXA-1 | 100 | 831 / 831 | NXIO01000090.1 Escherichia coli strain ST-131:E021 NODE_90_length_2384_cov_153.732_ID_179, whole genome shotgun sequence | 679..1509 | Beta-lactam resistance | [HQ170510](http://www.ncbi.nlm.nih.gov/nuccore/HQ170510) |

| **Aminoglycoside** | | | | | | |
| --- | --- | --- | --- | --- | --- | --- |
| **Resistance gene** | **Identity** | **Query / Template length** | **Contig** | **Position in contig** | **Predicted phenotype** | **Accession number** |
| aac(3)-IIa | 99.77 | 861 / 861 | NXIO01000084.1 Escherichia coli strain ST-131:E021 NODE_84_length_2798_cov_179.104_ID_167, whole genome shotgun sequence | 143..1003 | Aminoglycoside resistance | [X51534](http://www.ncbi.nlm.nih.gov/nuccore/X51534) |
| aac(6')-Ib-cr | 100 | 600 / 600 | NXIO01000090.1 Escherichia coli strain ST-131:E021 NODE_90_length_2384_cov_153.732_ID_179, whole genome shotgun sequence | 1640..2239 | Fluoroquinolone and aminoglycoside resistance | [DQ303918](http://www.ncbi.nlm.nih.gov/nuccore/DQ303918) |
| aadA5 | 100 | 789 / 789 | NXIO01000056.1 Escherichia coli strain ST-131:E021 NODE_56_length_9965_cov_136.992_ID_111, whole genome shotgun sequence | 1408..2196 | Aminoglycoside resistance | [AF137361](http://www.ncbi.nlm.nih.gov/nuccore/AF137361) |
| aph(3'')-Ib | 100 | 804 / 804 | NXIO01000062.1 Escherichia coli strain ST-131:E021 NODE_62_length_6854_cov_124.933_ID_123, whole genome shotgun sequence | 1456..2259 | Aminoglycoside resistance Alternate name; aph(3'')-Ib | [AF321551](http://www.ncbi.nlm.nih.gov/nuccore/AF321551) |
| aph(6)-Id | 100 | 831 / 831 | NXIO01000062.1 Escherichia coli strain ST-131:E021 NODE_62_length_6854_cov_124.933_ID_123, whole genome shotgun sequence | 2265..3095 | Aminoglycoside resistance Alternate name; aph(6)-Id | [CP000971](http://www.ncbi.nlm.nih.gov/nuccore/CP000971) |

extended output

Show Point mutation results

**Chromosomal point mutations - Results**

**Species:*escherichia_coli***

**Known Mutations**

| **rpoB** | | | | |
| --- | --- | --- | --- | --- |
| No mutations found in rpoB |  |  |  |  |

| **parE** | | | | |
| --- | --- | --- | --- | --- |
| **Mutation** | **Nucleotide change** | **Amino acid change** | **Resistance** | **PMID** |
| parE p.I529L | ATT ➝ CTT | I ➝ L | Nalidixic acid,Ciprofloxacin | [14506034](http://www.ncbi.nlm.nih.gov/pubmed/14506034) |

| **23S** | | | | |
| --- | --- | --- | --- | --- |
| No known mutations found in 23S |  |  |  |  |

| **pmrB** | | | | |
| --- | --- | --- | --- | --- |
| No known mutations found in pmrB |  |  |  |  |

| **gyrB** | | | | |
| --- | --- | --- | --- | --- |
| No known mutations found in gyrB |  |  |  |  |

| **ampC** | | | | |
| --- | --- | --- | --- | --- |
| No known mutations found in ampC |  |  |  |  |

| **pmrA** | | | | |
| --- | --- | --- | --- | --- |
| No known mutations found in pmrA |  |  |  |  |

| **16S_rrsB** | | | | |
| --- | --- | --- | --- | --- |
| No known mutations found in 16S_rrsB |  |  |  |  |

| **folP** | | | | |
| --- | --- | --- | --- | --- |
| No known mutations found in folP |  |  |  |  |

| **parC** | | | | |
| --- | --- | --- | --- | --- |
| **Mutation** | **Nucleotide change** | **Amino acid change** | **Resistance** | **PMID** |
| parC p.S80I | AGC ➝ ATT | S ➝ I | Nalidixic acid,Ciprofloxacin | [8851598](http://www.ncbi.nlm.nih.gov/pubmed/8851598) |
| parC p.E84V | GAA ➝ GTA | E ➝ V | Nalidixic acid,Ciprofloxacin | [12654733](http://www.ncbi.nlm.nih.gov/pubmed/12654733) |

| **16S_rrsC** | | | | |
| --- | --- | --- | --- | --- |
| No known mutations found in 16S_rrsC |  |  |  |  |

| **16S_rrsH** | | | | |
| --- | --- | --- | --- | --- |
| No known mutations found in 16S_rrsH |  |  |  |  |

| **gyrA** | | | | |
| --- | --- | --- | --- | --- |
| **Mutation** | **Nucleotide change** | **Amino acid change** | **Resistance** | **PMID** |
| gyrA p.S83L | TCG ➝ TTG | S ➝ L | Nalidixic acid,Ciprofloxacin | [8891148](http://www.ncbi.nlm.nih.gov/pubmed/8891148) |
| gyrA p.D87N | GAC ➝ AAC | D ➝ N | Nalidixic acid,Ciprofloxacin | [12654733](http://www.ncbi.nlm.nih.gov/pubmed/12654733) |

Top of Form

Bottom of Form


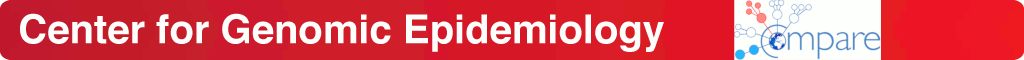


**ResFinder-3.2 Server - Results**

**Input Files: *E035.fasta***

Show Acquired antimicrobial resistance results

**Acquired antimicrobial resistance gene - Results**

| **Beta-lactam** | | | | | | |
| --- | --- | --- | --- | --- | --- | --- |
| **Resistance gene** | **Identity** | **Query / Template length** | **Contig** | **Position in contig** | **Predicted phenotype** | **Accession number** |
| blaCTX-M-15 | 100 | 876 / 876 | NXJC01000041.1 Escherichia coli strain ST-998:E035 contig_41, whole genome shotgun sequence | 3828..4703 | Beta-lactam resistance Alternate name; UOE-1 | [AY044436](http://www.ncbi.nlm.nih.gov/nuccore/AY044436) |
| blaTEM-1B | 100 | 861 / 861 | NXJC01000041.1 Escherichia coli strain ST-998:E035 contig_41, whole genome shotgun sequence | 146..1006 | Beta-lactam resistance Alternate name; RblaTEM-1 | [AY458016](http://www.ncbi.nlm.nih.gov/nuccore/AY458016) |

| **Aminoglycoside** | | | | | | |
| --- | --- | --- | --- | --- | --- | --- |
| **Resistance gene** | **Identity** | **Query / Template length** | **Contig** | **Position in contig** | **Predicted phenotype** | **Accession number** |
| aadA1 | 100 | 792 / 792 | NXJC01000036.1 Escherichia coli strain ST-998:E035 contig_36, whole genome shotgun sequence | 7763..8554 | Aminoglycoside resistance | [JX185132](http://www.ncbi.nlm.nih.gov/nuccore/JX185132) |

| **Trimethoprim** | | | | | | |
| --- | --- | --- | --- | --- | --- | --- |
| **Resistance gene** | **Identity** | **Query / Template length** | **Contig** | **Position in contig** | **Predicted phenotype** | **Accession number** |
| dfrA1 | 99.79 | 474 / 474 | NXJC01000036.1 Escherichia coli strain ST-998:E035 contig_36, whole genome shotgun sequence | 8647..9120 | Trimethoprim resistance | [AF203818](http://www.ncbi.nlm.nih.gov/nuccore/AF203818) |
| dfrA1 | 99.79 | 474 / 474 | NXJC01000036.1 Escherichia coli strain ST-998:E035 contig_36, whole genome shotgun sequence | 8647..9120 | Trimethoprim resistance | [AJ238350](http://www.ncbi.nlm.nih.gov/nuccore/AJ238350) |
| dfrA1 | 99.79 | 474 / 474 | NXJC01000036.1 Escherichia coli strain ST-998:E035 contig_36, whole genome shotgun sequence | 8647..9120 | Trimethoprim resistance | [X00926](http://www.ncbi.nlm.nih.gov/nuccore/X00926) |

| **Colistin** |
| --- |

| **Sulphonamide** | | | | | | |
| --- | --- | --- | --- | --- | --- | --- |
| **Resistance gene** | **Identity** | **Query / Template length** | **Contig** | **Position in contig** | **Predicted phenotype** | **Accession number** |
| sul1 | 100 | 840 / 840 | NXJC01000036.1 Escherichia coli strain ST-998:E035 contig_36, whole genome shotgun sequence | 6419..7258 | Sulphonamide resistance | [U12338](http://www.ncbi.nlm.nih.gov/nuccore/U12338) |

| **Tetracycline** | | | | | | |
| --- | --- | --- | --- | --- | --- | --- |
| **Resistance gene** | **Identity** | **Query / Template length** | **Contig** | **Position in contig** | **Predicted phenotype** | **Accession number** |
| tet(B) | 100 | 1206 / 1206 | NXJC01000130.1 Escherichia coli strain ST-998:E035 contig_130, whole genome shotgun sequence | 1229..2434 | Tetracycline resistance | [AF326777](http://www.ncbi.nlm.nih.gov/nuccore/AF326777) |

| **Macrolide** | | | | | | |
| --- | --- | --- | --- | --- | --- | --- |
| **Resistance gene** | **Identity** | **Query / Template length** | **Contig** | **Position in contig** | **Predicted phenotype** | **Accession number** |
| mdf(A) | 97.97 | 1233 / 1233 | NXJC01000042.1 Escherichia coli strain ST-998:E035 contig_42, whole genome shotgun sequence | 4454..5686 | Warning: gene is missing from Notes file. Please inform curator. | [Y08743](http://www.ncbi.nlm.nih.gov/nuccore/Y08743) |


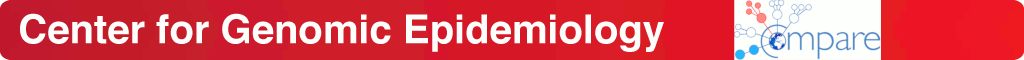


|  | Home | Services | Instructions | Output | Overview of genes | Article abstract |  |
| --- | --- | --- | --- | --- | --- | --- | --- |

**ResFinder-3.2 Server - Results**

**Input Files: *E040.fasta***

Show Acquired antimicrobial resistance results

**Acquired antimicrobial resistance gene - Results**

| **Sulphonamide** | | | | | | |
| --- | --- | --- | --- | --- | --- | --- |
| **Resistance gene** | **Identity** | **Query / Template length** | **Contig** | **Position in contig** | **Predicted phenotype** | **Accession number** |
| sul2 | 100 | 816 / 816 | NXIP01000084.1 Escherichia coli strain ST-95:E040 NODE_84_length_2903_cov_70.7707_ID_167, whole genome shotgun sequence | 128..943 | Sulphonamide resistance | [HQ840942](http://www.ncbi.nlm.nih.gov/nuccore/HQ840942) |
| sul3 | 100 | 792 / 792 | NXIP01000083.1 Escherichia coli strain ST-95:E040 NODE_83_length_2935_cov_77.4944_ID_165, whole genome shotgun sequence | 1971..2762 | Sulphonamide resistance | [AJ459418](http://www.ncbi.nlm.nih.gov/nuccore/AJ459418) |

| **Trimethoprim** | | | | | | |
| --- | --- | --- | --- | --- | --- | --- |
| **Resistance gene** | **Identity** | **Query / Template length** | **Contig** | **Position in contig** | **Predicted phenotype** | **Accession number** |
| dfrA5 | 100 | 474 / 474 | NXIP01000119.1 Escherichia coli strain ST-95:E040 NODE_119_length_887_cov_179.188_ID_237, whole genome shotgun sequence | 199..672 | Trimethoprim resistance | [X12868](http://www.ncbi.nlm.nih.gov/nuccore/X12868) |

| **Phenicol** | | | | | | |
| --- | --- | --- | --- | --- | --- | --- |
| **Resistance gene** | **Identity** | **Query / Template length** | **Contig** | **Position in contig** | **Predicted phenotype** | **Accession number** |
| cmlA1 | 99.92 | 1260 / 1260 | NXIP01000055.1 Escherichia coli strain ST-95:E040 NODE_55_length_10536_cov_146.169_ID_109, whole genome shotgun sequence | 2599..3858 | Phenicol resistance | [M64556](http://www.ncbi.nlm.nih.gov/nuccore/M64556) |

| **Beta-lactam** | | | | | | |
| --- | --- | --- | --- | --- | --- | --- |
| **Resistance gene** | **Identity** | **Query / Template length** | **Contig** | **Position in contig** | **Predicted phenotype** | **Accession number** |
| blaCTX-M-14 | 100 | 876 / 876 | NXIP01000107.1 Escherichia coli strain ST-95:E040 NODE_107_length_1376_cov_92.8724_ID_213, whole genome shotgun sequence | 349..1224 | Beta-lactam resistance Amino acid sequences of CTX-M-14 and CTX-M-18 are identical | [AF252622](http://www.ncbi.nlm.nih.gov/nuccore/AF252622) |
| blaTEM-1B | 100 | 861 / 861 | NXIP01000089.1 Escherichia coli strain ST-95:E040 NODE_89_length_2114_cov_129.714_ID_177, whole genome shotgun sequence | 634..1494 | Beta-lactam resistance Alternate name; RblaTEM-1 | [AY458016](http://www.ncbi.nlm.nih.gov/nuccore/AY458016) |

| **Macrolide** | | | | | | |
| --- | --- | --- | --- | --- | --- | --- |
| **Resistance gene** | **Identity** | **Query / Template length** | **Contig** | **Position in contig** | **Predicted phenotype** | **Accession number** |
| mdf(A) | 97.81 | 1233 / 1233 | NXIP01000004.1 Escherichia coli strain ST-95:E040 NODE_4_length_311653_cov_117.437_ID_7, whole genome shotgun sequence | 250787..252019 | Warning: gene is missing from Notes file. Please inform curator. | [Y08743](http://www.ncbi.nlm.nih.gov/nuccore/Y08743) |

| **Aminoglycoside** | | | | | | |
| --- | --- | --- | --- | --- | --- | --- |
| **Resistance gene** | **Identity** | **Query / Template length** | **Contig** | **Position in contig** | **Predicted phenotype** | **Accession number** |
| aadA1 | 100 | 792 / 792 | NXIP01000055.1 Escherichia coli strain ST-95:E040 NODE_55_length_10536_cov_146.169_ID_109, whole genome shotgun sequence | 1715..2506 | Aminoglycoside resistance | [JQ414041](http://www.ncbi.nlm.nih.gov/nuccore/JQ414041) |
| aadA2b | 99.87 | 780 / 780 | NXIP01000055.1 Escherichia coli strain ST-95:E040 NODE_55_length_10536_cov_146.169_ID_109, whole genome shotgun sequence | 4120..4899 | Warning: gene is missing from Notes file. Please inform curator. | [D43625](http://www.ncbi.nlm.nih.gov/nuccore/D43625) |
| aph(3'')-Ib | 100 | 803 / 804 | NXIP01000099.1 Escherichia coli strain ST-95:E040 NODE_99_length_1680_cov_321.589_ID_197, whole genome shotgun sequence | 845..1647 | Aminoglycoside resistance Alternate name; aph(3'')-Ib | [AF024602](http://www.ncbi.nlm.nih.gov/nuccore/AF024602) |
| aph(3'')-Ib | 99.88 | 804 / 804 | NXIP01000099.1 Escherichia coli strain ST-95:E040 NODE_99_length_1680_cov_321.589_ID_197, whole genome shotgun sequence | 845..1648 | Aminoglycoside resistance Alternate name; aph(3'')-Ib | [AF313472](http://www.ncbi.nlm.nih.gov/nuccore/AF313472) |
| aph(3'')-Ib | 99.88 | 804 / 804 | NXIP01000099.1 Escherichia coli strain ST-95:E040 NODE_99_length_1680_cov_321.589_ID_197, whole genome shotgun sequence | 845..1648 | Aminoglycoside resistance Alternate name; aph(3'')-Ib | [AF321550](http://www.ncbi.nlm.nih.gov/nuccore/AF321550) |
| aph(3'')-Ib | 99.88 | 804 / 804 | NXIP01000099.1 Escherichia coli strain ST-95:E040 NODE_99_length_1680_cov_321.589_ID_197, whole genome shotgun sequence | 845..1648 | Aminoglycoside resistance Alternate name; aph(3'')-Ib | [AF321551](http://www.ncbi.nlm.nih.gov/nuccore/AF321551) |
| aph(3')-IIa | 100 | 795 / 795 | NXIP01000086.1 Escherichia coli strain ST-95:E040 NODE_86_length_2695_cov_148.314_ID_171, whole genome shotgun sequence | 1559..2353 | Aminoglycoside resistance | [V00618](http://www.ncbi.nlm.nih.gov/nuccore/V00618) |
| aph(6)-Id | 100 | 837 / 837 | NXIP01000099.1 Escherichia coli strain ST-95:E040 NODE_99_length_1680_cov_321.589_ID_197, whole genome shotgun sequence | 9..845 | Aminoglycoside resistance Alternate name; aph(6)-Id | [M28829](http://www.ncbi.nlm.nih.gov/nuccore/M28829) |

| **Fosfomycin** | | | | | | |
| --- | --- | --- | --- | --- | --- | --- |
| **Resistance gene** | **Identity** | **Query / Template length** | **Contig** | **Position in contig** | **Predicted phenotype** | **Accession number** |
| fosA3 | 100 | 417 / 417 | NXIP01000091.1 Escherichia coli strain ST-95:E040 NODE_91_length_2094_cov_141.083_ID_181, whole genome shotgun sequence | 1321..1737 | Fosfomycin resistance | [AB522970](http://www.ncbi.nlm.nih.gov/nuccore/AB522970) |

Show Point mutation results

**Chromosomal point mutations - Results**

**Species:*escherichia_coli***

**Known Mutations**

| **gyrB** | | | | |
| --- | --- | --- | --- | --- |
| No known mutations found in gyrB |  |  |  |  |

| **16S_rrsC** | | | | |
| --- | --- | --- | --- | --- |
| No known mutations found in 16S_rrsC |  |  |  |  |

| **parC** | | | | |
| --- | --- | --- | --- | --- |
| No known mutations found in parC |  |  |  |  |

| **gyrA** | | | | |
| --- | --- | --- | --- | --- |
| No known mutations found in gyrA |  |  |  |  |

| **rpoB** | | | | |
| --- | --- | --- | --- | --- |
| No mutations found in rpoB |  |  |  |  |

| **16S_rrsH** | | | | |
| --- | --- | --- | --- | --- |
| No known mutations found in 16S_rrsH |  |  |  |  |

| **pmrB** | | | | |
| --- | --- | --- | --- | --- |
| No known mutations found in pmrB |  |  |  |  |

| **pmrA** | | | | |
| --- | --- | --- | --- | --- |
| No known mutations found in pmrA |  |  |  |  |

| **folP** | | | | |
| --- | --- | --- | --- | --- |
| No known mutations found in folP |  |  |  |  |

| **ampC** | | | | |
| --- | --- | --- | --- | --- |
| No known mutations found in ampC |  |  |  |  |

| **23S** | | | | |
| --- | --- | --- | --- | --- |
| No known mutations found in 23S |  |  |  |  |

| **parE** | | | | |
| --- | --- | --- | --- | --- |
| No known mutations found in parE |  |  |  |  |

| **16S_rrsB** | | | | |
| --- | --- | --- | --- | --- |
| No known mutations found in 16S_rrsB |  |  |  |  |

Top of Form

Bottom of Form

Top of Form

Bottom of Form

**CITATIONS**

For publication of results, please cite:


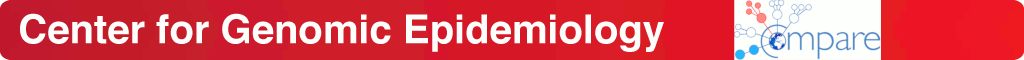


|  | Home | Services | Instructions | Output | Overview of genes | Article abstract |  |
| --- | --- | --- | --- | --- | --- | --- | --- |

**ResFinder-3.2 Server - Results**

**Input Files: *E053.fasta***

Show Acquired antimicrobial resistance results

**Acquired antimicrobial resistance gene - Results**

| **Sulphonamide** | | | | | | |
| --- | --- | --- | --- | --- | --- | --- |
| **Resistance gene** | **Identity** | **Query / Template length** | **Contig** | **Position in contig** | **Predicted phenotype** | **Accession number** |
| sul1 | 100 | 761 / 828 | NXIR01000080.1 Escherichia coli strain ST-73:E053 NODE_80_length_4325_cov_65.4411_ID_159, whole genome shotgun sequence | 3466..4226 | Sulphonamide resistance | [AY522923](http://www.ncbi.nlm.nih.gov/nuccore/AY522923) |
| sul1 | 100 | 761 / 840 | NXIR01000080.1 Escherichia coli strain ST-73:E053 NODE_80_length_4325_cov_65.4411_ID_159, whole genome shotgun sequence | 3466..4226 | Sulphonamide resistance | [X15024](http://www.ncbi.nlm.nih.gov/nuccore/X15024) |
| sul2 | 100 | 816 / 816 | NXIR01000077.1 Escherichia coli strain ST-73:E053 NODE_77_length_4582_cov_62.3674_ID_153, whole genome shotgun sequence | 1808..2623 | Sulphonamide resistance | [HQ840942](http://www.ncbi.nlm.nih.gov/nuccore/HQ840942) |

| **Tetracycline** | | | | | | |
| --- | --- | --- | --- | --- | --- | --- |
| **Resistance gene** | **Identity** | **Query / Template length** | **Contig** | **Position in contig** | **Predicted phenotype** | **Accession number** |
| tet(A) | 100 | 1200 / 1200 | NXIR01000045.1 Escherichia coli strain ST-73:E053 NODE_45_length_11767_cov_106.183_ID_89, whole genome shotgun sequence | 2181..3380 | Tetracycline resistance | [AJ517790](http://www.ncbi.nlm.nih.gov/nuccore/AJ517790) |

| **Aminoglycoside** | | | | | | |
| --- | --- | --- | --- | --- | --- | --- |
| **Resistance gene** | **Identity** | **Query / Template length** | **Contig** | **Position in contig** | **Predicted phenotype** | **Accession number** |
| aac(3)-IIa | 99.77 | 861 / 861 | NXIR01000093.1 Escherichia coli strain ST-73:E053 NODE_93_length_2841_cov_190.367_ID_185, whole genome shotgun sequence | 186..1046 | Aminoglycoside resistance | [X51534](http://www.ncbi.nlm.nih.gov/nuccore/X51534) |
| aac(6')-Ib-cr | 100 | 600 / 600 | NXIR01000103.1 Escherichia coli strain ST-73:E053 NODE_103_length_2384_cov_166.905_ID_205, whole genome shotgun sequence | 1640..2239 | Fluoroquinolone and aminoglycoside resistance | [DQ303918](http://www.ncbi.nlm.nih.gov/nuccore/DQ303918) |
| aph(3'')-Ib | 100 | 804 / 804 | NXIR01000077.1 Escherichia coli strain ST-73:E053 NODE_77_length_4582_cov_62.3674_ID_153, whole genome shotgun sequence | 944..1747 | Aminoglycoside resistance Alternate name; aph(3'')-Ib | [AF321551](http://www.ncbi.nlm.nih.gov/nuccore/AF321551) |
| aph(3')-Ia | 100 | 816 / 816 | NXIR01000137.1 Escherichia coli strain ST-73:E053 NODE_137_length_1281_cov_123.984_ID_273, whole genome shotgun sequence | 230..1045 | Aminoglycoside resistance | [V00359](http://www.ncbi.nlm.nih.gov/nuccore/V00359) |
| aph(6)-Id | 100 | 837 / 837 | NXIR01000077.1 Escherichia coli strain ST-73:E053 NODE_77_length_4582_cov_62.3674_ID_153, whole genome shotgun sequence | 108..944 | Aminoglycoside resistance Alternate name; aph(6)-Id | [M28829](http://www.ncbi.nlm.nih.gov/nuccore/M28829) |

| **Quinolone** | | | | | | |
| --- | --- | --- | --- | --- | --- | --- |
| **Resistance gene** | **Identity** | **Query / Template length** | **Contig** | **Position in contig** | **Predicted phenotype** | **Accession number** |
| aac(6')-Ib-cr | 100 | 600 / 600 | NXIR01000103.1 Escherichia coli strain ST-73:E053 NODE_103_length_2384_cov_166.905_ID_205, whole genome shotgun sequence | 1640..2239 | Fluoroquinolone and aminoglycoside resistance | [DQ303918](http://www.ncbi.nlm.nih.gov/nuccore/DQ303918) |

| **Phenicol** | | | | | | |
| --- | --- | --- | --- | --- | --- | --- |
| **Resistance gene** | **Identity** | **Query / Template length** | **Contig** | **Position in contig** | **Predicted phenotype** | **Accession number** |
| catA1 | 99.85 | 660 / 660 | NXIR01000110.1 Escherichia coli strain ST-73:E053 NODE_110_length_2220_cov_68.7492_ID_219, whole genome shotgun sequence | 1239..1898 | Phenicol resistance | [V00622](http://www.ncbi.nlm.nih.gov/nuccore/V00622) |
| catB3 | 100 | 442 / 633 | NXIR01000103.1 Escherichia coli strain ST-73:E053 NODE_103_length_2384_cov_166.905_ID_205, whole genome shotgun sequence | 100..541 | Phenicol resistance | [AJ009818](http://www.ncbi.nlm.nih.gov/nuccore/AJ009818) |
| catB3 | 100 | 442 / 633 | NXIR01000103.1 Escherichia coli strain ST-73:E053 NODE_103_length_2384_cov_166.905_ID_205, whole genome shotgun sequence | 100..541 | Phenicol resistance | [U13880](http://www.ncbi.nlm.nih.gov/nuccore/U13880) |

| **Trimethoprim** | | | | | | |
| --- | --- | --- | --- | --- | --- | --- |
| **Resistance gene** | **Identity** | **Query / Template length** | **Contig** | **Position in contig** | **Predicted phenotype** | **Accession number** |
| dfrA7 | 100 | 474 / 474 | NXIR01000080.1 Escherichia coli strain ST-73:E053 NODE_80_length_4325_cov_65.4411_ID_159, whole genome shotgun sequence | 2422..2895 | Trimethoprim resistance | [AB161450](http://www.ncbi.nlm.nih.gov/nuccore/AB161450) |

| **Beta-lactam** | | | | | | |
| --- | --- | --- | --- | --- | --- | --- |
| **Resistance gene** | **Identity** | **Query / Template length** | **Contig** | **Position in contig** | **Predicted phenotype** | **Accession number** |
| blaCTX-M-15 | 100 | 876 / 876 | NXIR01000060.1 Escherichia coli strain ST-73:E053 NODE_60_length_6894_cov_156.76_ID_119, whole genome shotgun sequence | 2780..3655 | Beta-lactam resistance Alternate name; UOE-1 | [AY044436](http://www.ncbi.nlm.nih.gov/nuccore/AY044436) |
| blaOXA-1 | 100 | 831 / 831 | NXIR01000103.1 Escherichia coli strain ST-73:E053 NODE_103_length_2384_cov_166.905_ID_205, whole genome shotgun sequence | 679..1509 | Beta-lactam resistance | [HQ170510](http://www.ncbi.nlm.nih.gov/nuccore/HQ170510) |

| **Macrolide** | | | | | | |
| --- | --- | --- | --- | --- | --- | --- |
| **Resistance gene** | **Identity** | **Query / Template length** | **Contig** | **Position in contig** | **Predicted phenotype** | **Accession number** |
| mdf(A) | 98.13 | 1233 / 1233 | NXIR01000004.1 Escherichia coli strain ST-73:E053 NODE_4_length_296822_cov_91.0986_ID_7, whole genome shotgun sequence | 194825..196057 | Warning: gene is missing from Notes file. Please inform curator. | [Y08743](http://www.ncbi.nlm.nih.gov/nuccore/Y08743) |

Show Point mutation results

**Chromosomal point mutations - Results**

**Species:*escherichia_coli***

**Known Mutations**

| **gyrA** | | | | |
| --- | --- | --- | --- | --- |
| No known mutations found in gyrA |  |  |  |  |

| **parE** | | | | |
| --- | --- | --- | --- | --- |
| No known mutations found in parE |  |  |  |  |

| **pmrA** | | | | |
| --- | --- | --- | --- | --- |
| No known mutations found in pmrA |  |  |  |  |

| **16S_rrsC** | | | | |
| --- | --- | --- | --- | --- |
| No known mutations found in 16S_rrsC |  |  |  |  |

| **23S** | | | | |
| --- | --- | --- | --- | --- |
| No known mutations found in 23S |  |  |  |  |

| **folP** | | | | |
| --- | --- | --- | --- | --- |
| No mutations found in folP |  |  |  |  |

| **16S_rrsB** | | | | |
| --- | --- | --- | --- | --- |
| No known mutations found in 16S_rrsB |  |  |  |  |

| **rpoB** | | | | |
| --- | --- | --- | --- | --- |
| No mutations found in rpoB |  |  |  |  |

| **parC** | | | | |
| --- | --- | --- | --- | --- |
| No known mutations found in parC |  |  |  |  |

| **gyrB** | | | | |
| --- | --- | --- | --- | --- |
| No known mutations found in gyrB |  |  |  |  |

| **pmrB** | | | | |
| --- | --- | --- | --- | --- |
| No known mutations found in pmrB |  |  |  |  |

| **ampC** | | | | |
| --- | --- | --- | --- | --- |
| No mutations found in ampC |  |  |  |  |

| **16S_rrsH** | | | | |
| --- | --- | --- | --- | --- |
| No known mutations found in 16S_rrsH |  |  |  |  |

Top of Form

Bottom of Form


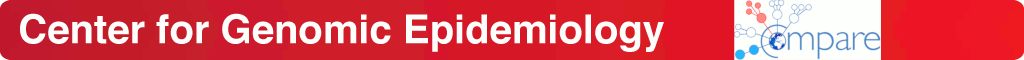


|  | Home | Services | Instructions | Output | Overview of genes | Article abstract |  |
| --- | --- | --- | --- | --- | --- | --- | --- |

**ResFinder-3.2 Server - Results**

**Input Files: *E056.fasta***

Show Acquired antimicrobial resistance results

**Acquired antimicrobial resistance gene - Results**

| **Phenicol** | | | | | | |
| --- | --- | --- | --- | --- | --- | --- |
| **Resistance gene** | **Identity** | **Query / Template length** | **Contig** | **Position in contig** | **Predicted phenotype** | **Accession number** |
| catB3 | 100 | 442 / 633 | NXJD01000102.1 Escherichia coli strain ST-131:E056 NODE_102_length_2296_cov_37.5359, whole genome shotgun sequence | 56..497 | Phenicol resistance | [AJ009818](http://www.ncbi.nlm.nih.gov/nuccore/AJ009818) |
| catB3 | 100 | 442 / 633 | NXJD01000102.1 Escherichia coli strain ST-131:E056 NODE_102_length_2296_cov_37.5359, whole genome shotgun sequence | 56..497 | Phenicol resistance | [U13880](http://www.ncbi.nlm.nih.gov/nuccore/U13880) |

| **Macrolide** | | | | | | |
| --- | --- | --- | --- | --- | --- | --- |
| **Resistance gene** | **Identity** | **Query / Template length** | **Contig** | **Position in contig** | **Predicted phenotype** | **Accession number** |
| mdf(A) | 97.81 | 1233 / 1233 | NXJD01000011.1 Escherichia coli strain ST-131:E056 NODE_11_length_156556_cov_39.1534, whole genome shotgun sequence | 55638..56870 | Warning: gene is missing from Notes file. Please inform curator. | [Y08743](http://www.ncbi.nlm.nih.gov/nuccore/Y08743) |

| **Tetracycline** | | | | | | |
| --- | --- | --- | --- | --- | --- | --- |
| **Resistance gene** | **Identity** | **Query / Template length** | **Contig** | **Position in contig** | **Predicted phenotype** | **Accession number** |
| tet(A) | 100 | 1200 / 1200 | NXJD01000053.1 Escherichia coli strain ST-131:E056 NODE_53_length_10102_cov_31.5745, whole genome shotgun sequence | 7999..9198 | Tetracycline resistance | [AJ517790](http://www.ncbi.nlm.nih.gov/nuccore/AJ517790) |

| **Aminoglycoside** | | | | | | |
| --- | --- | --- | --- | --- | --- | --- |
| **Resistance gene** | **Identity** | **Query / Template length** | **Contig** | **Position in contig** | **Predicted phenotype** | **Accession number** |
| aac(3)-IIa | 99.77 | 861 / 861 | NXJD01000097.1 Escherichia coli strain ST-131:E056 NODE_97_length_2753_cov_40.0626, whole genome shotgun sequence | 142..1002 | Aminoglycoside resistance | [X51534](http://www.ncbi.nlm.nih.gov/nuccore/X51534) |
| aac(6')-Ib-cr | 100 | 600 / 600 | NXJD01000102.1 Escherichia coli strain ST-131:E056 NODE_102_length_2296_cov_37.5359, whole genome shotgun sequence | 1596..2195 | Fluoroquinolone and aminoglycoside resistance | [DQ303918](http://www.ncbi.nlm.nih.gov/nuccore/DQ303918) |

| **Beta-lactam** | | | | | | |
| --- | --- | --- | --- | --- | --- | --- |
| **Resistance gene** | **Identity** | **Query / Template length** | **Contig** | **Position in contig** | **Predicted phenotype** | **Accession number** |
| blaCTX-M-15 | 100 | 876 / 876 | NXJD01000084.1 Escherichia coli strain ST-131:E056 NODE_84_length_4202_cov_36.3012, whole genome shotgun sequence | 1981..2856 | Beta-lactam resistance Alternate name; UOE-1 | [AY044436](http://www.ncbi.nlm.nih.gov/nuccore/AY044436) |
| blaOXA-1 | 100 | 831 / 831 | NXJD01000102.1 Escherichia coli strain ST-131:E056 NODE_102_length_2296_cov_37.5359, whole genome shotgun sequence | 635..1465 | Beta-lactam resistance | [HQ170510](http://www.ncbi.nlm.nih.gov/nuccore/HQ170510) |

| **Quinolone** | | | | | | |
| --- | --- | --- | --- | --- | --- | --- |
| **Resistance gene** | **Identity** | **Query / Template length** | **Contig** | **Position in contig** | **Predicted phenotype** | **Accession number** |
| aac(6')-Ib-cr | 100 | 600 / 600 | NXJD01000102.1 Escherichia coli strain ST-131:E056 NODE_102_length_2296_cov_37.5359, whole genome shotgun sequence | 1596..2195 | Fluoroquinolone and aminoglycoside resistance | [DQ303918](http://www.ncbi.nlm.nih.gov/nuccore/DQ303918) |

**Chromosomal point mutations - Results**

**Species:*escherichia_coli***

**Known Mutations**

| **23S** | | | | |
| --- | --- | --- | --- | --- |
| No known mutations found in 23S |  |  |  |  |

| **16S_rrsC** | | | | |
| --- | --- | --- | --- | --- |
| No known mutations found in 16S_rrsC |  |  |  |  |

| **ampC** | | | | |
| --- | --- | --- | --- | --- |
| No known mutations found in ampC |  |  |  |  |

| **parE** | | | | |
| --- | --- | --- | --- | --- |
| **Mutation** | **Nucleotide change** | **Amino acid change** | **Resistance** | **PMID** |
| parE p.I529L | ATT ➝ CTT | I ➝ L | Nalidixic acid,Ciprofloxacin | [14506034](http://www.ncbi.nlm.nih.gov/pubmed/14506034) |

| **gyrA** | | | | |
| --- | --- | --- | --- | --- |
| **Mutation** | **Nucleotide change** | **Amino acid change** | **Resistance** | **PMID** |
| gyrA p.S83L | TCG ➝ TTG | S ➝ L | Nalidixic acid,Ciprofloxacin | [8891148](http://www.ncbi.nlm.nih.gov/pubmed/8891148) |
| gyrA p.D87N | GAC ➝ AAC | D ➝ N | Nalidixic acid,Ciprofloxacin | [12654733](http://www.ncbi.nlm.nih.gov/pubmed/12654733) |

| **gyrB** | | | | |
| --- | --- | --- | --- | --- |
| No known mutations found in gyrB |  |  |  |  |

| **pmrB** | | | | |
| --- | --- | --- | --- | --- |
| No known mutations found in pmrB |  |  |  |  |

| **folP** | | | | |
| --- | --- | --- | --- | --- |
| No known mutations found in folP |  |  |  |  |

| **16S_rrsB** | | | | |
| --- | --- | --- | --- | --- |
| No known mutations found in 16S_rrsB |  |  |  |  |

| **parC** | | | | |
| --- | --- | --- | --- | --- |
| **Mutation** | **Nucleotide change** | **Amino acid change** | **Resistance** | **PMID** |
| parC p.S80I | AGC ➝ ATT | S ➝ I | Nalidixic acid,Ciprofloxacin | [8851598](http://www.ncbi.nlm.nih.gov/pubmed/8851598) |
| parC p.E84V | GAA ➝ GTA | E ➝ V | Nalidixic acid,Ciprofloxacin | [12654733](http://www.ncbi.nlm.nih.gov/pubmed/12654733) |

| **16S_rrsH** | | | | |
| --- | --- | --- | --- | --- |
| No known mutations found in 16S_rrsH |  |  |  |  |

| **rpoB** | | | | |
| --- | --- | --- | --- | --- |
| No mutations found in rpoB |  |  |  |  |

| **pmrA** | | | | |
| --- | --- | --- | --- | --- |
| No known mutations found in pmrA |  |  |  |  |

Top of Form


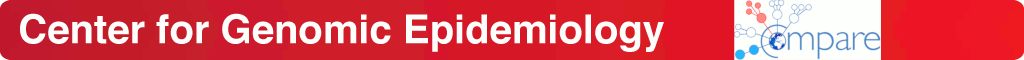


**ResFinder-3.2 Server - Results**

**Input Files: *E058.fasta***

Show Acquired antimicrobial resistance results

| **Macrolide** | | | | | | |
| --- | --- | --- | --- | --- | --- | --- |
| **Resistance gene** | **Identity** | **Query / Template length** | **Contig** | **Position in contig** | **Predicted phenotype** | **Accession number** |
| mdf(A) | 97.81 | 1233 / 1233 | NXLI01000005.1 Escherichia coli strain ST-131:E058 NODE_5_length_284479_cov_58.0191_ID_9, whole genome shotgun sequence | 172769..174001 | Warning: gene is missing from Notes file. Please inform curator. | [Y08743](http://www.ncbi.nlm.nih.gov/nuccore/Y08743) |

| **Colistin** |
| --- |

| **Phenicol** | | | | | | |
| --- | --- | --- | --- | --- | --- | --- |
| **Resistance gene** | **Identity** | **Query / Template length** | **Contig** | **Position in contig** | **Predicted phenotype** | **Accession number** |
| catB3 | 100 | 442 / 633 | NXLI01000066.1 Escherichia coli strain ST-131:E058 NODE_66_length_2384_cov_63.1182_ID_131, whole genome shotgun sequence | 100..541 | Phenicol resistance | [AJ009818](http://www.ncbi.nlm.nih.gov/nuccore/AJ009818) |
| catB3 | 100 | 442 / 633 | NXLI01000066.1 Escherichia coli strain ST-131:E058 NODE_66_length_2384_cov_63.1182_ID_131, whole genome shotgun sequence | 100..541 | Phenicol resistance | [U13880](http://www.ncbi.nlm.nih.gov/nuccore/U13880) |

| **Quinolone** | | | | | | |
| --- | --- | --- | --- | --- | --- | --- |
| **Resistance gene** | **Identity** | **Query / Template length** | **Contig** | **Position in contig** | **Predicted phenotype** | **Accession number** |
| aac(6')-Ib-cr | 100 | 600 / 600 | NXLI01000066.1 Escherichia coli strain ST-131:E058 NODE_66_length_2384_cov_63.1182_ID_131, whole genome shotgun sequence | 1640..2239 | Fluoroquinolone and aminoglycoside resistance | [DQ303918](http://www.ncbi.nlm.nih.gov/nuccore/DQ303918) |

| **Beta-lactam** | | | | | | |
| --- | --- | --- | --- | --- | --- | --- |
| **Resistance gene** | **Identity** | **Query / Template length** | **Contig** | **Position in contig** | **Predicted phenotype** | **Accession number** |
| blaCTX-M-15 | 100 | 876 / 876 | NXLI01000051.1 Escherichia coli strain ST-131:E058 NODE_51_length_4247_cov_68.2392_ID_101, whole genome shotgun sequence | 1391..2266 | Beta-lactam resistance Alternate name; UOE-1 | [AY044436](http://www.ncbi.nlm.nih.gov/nuccore/AY044436) |
| blaOXA-1 | 100 | 831 / 831 | NXLI01000066.1 Escherichia coli strain ST-131:E058 NODE_66_length_2384_cov_63.1182_ID_131, whole genome shotgun sequence | 679..1509 | Beta-lactam resistance | [HQ170510](http://www.ncbi.nlm.nih.gov/nuccore/HQ170510) |

| **Aminoglycoside** | | | | | | |
| --- | --- | --- | --- | --- | --- | --- |
| **Resistance gene** | **Identity** | **Query / Template length** | **Contig** | **Position in contig** | **Predicted phenotype** | **Accession number** |
| aac(3)-IIa | 99.77 | 861 / 861 | NXLI01000061.1 Escherichia coli strain ST-131:E058 NODE_61_length_2798_cov_34.0133_ID_121, whole genome shotgun sequence | 1796..2656 | Aminoglycoside resistance | [X51534](http://www.ncbi.nlm.nih.gov/nuccore/X51534) |
| aac(6')-Ib-cr | 100 | 600 / 600 | NXLI01000066.1 Escherichia coli strain ST-131:E058 NODE_66_length_2384_cov_63.1182_ID_131, whole genome shotgun sequence | 1640..2239 | Fluoroquinolone and aminoglycoside resistance | [DQ303918](http://www.ncbi.nlm.nih.gov/nuccore/DQ303918) |

| **Tetracycline** | | | | | | |
| --- | --- | --- | --- | --- | --- | --- |
| **Resistance gene** | **Identity** | **Query / Template length** | **Contig** | **Position in contig** | **Predicted phenotype** | **Accession number** |
| tet(A) | 100 | 1200 / 1200 | NXLI01000042.1 Escherichia coli strain ST-131:E058 NODE_42_length_9712_cov_37.4924_ID_83, whole genome shotgun sequence | 8043..9242 | Tetracycline resistance | [AJ517790](http://www.ncbi.nlm.nih.gov/nuccore/AJ517790) |

extended output

**Selected %ID threshold:  *90 %***

**Selected minimum length:  *60 %***

Top of Form

Bottom of Form

Top of Form

Bottom of Form

Top of Form

Bottom of Form

Top of Form

Bottom of Form

Show Point mutation results

**Chromosomal point mutations - Results**

**Species:*escherichia_coli***

**Known Mutations**

| **parC** | | | | |
| --- | --- | --- | --- | --- |
| **Mutation** | **Nucleotide change** | **Amino acid change** | **Resistance** | **PMID** |
| parC p.S80I | AGC ➝ ATT | S ➝ I | Nalidixic acid,Ciprofloxacin | [8851598](http://www.ncbi.nlm.nih.gov/pubmed/8851598) |
| parC p.E84V | GAA ➝ GTA | E ➝ V | Nalidixic acid,Ciprofloxacin | [12654733](http://www.ncbi.nlm.nih.gov/pubmed/12654733) |

| **ampC** | | | | |
| --- | --- | --- | --- | --- |
| No known mutations found in ampC |  |  |  |  |

| **rpoB** | | | | |
| --- | --- | --- | --- | --- |
| No mutations found in rpoB |  |  |  |  |

| **parE** | | | | |
| --- | --- | --- | --- | --- |
| **Mutation** | **Nucleotide change** | **Amino acid change** | **Resistance** | **PMID** |
| parE p.I529L | ATT ➝ CTT | I ➝ L | Nalidixic acid,Ciprofloxacin | [14506034](http://www.ncbi.nlm.nih.gov/pubmed/14506034) |

| **23S** | | | | |
| --- | --- | --- | --- | --- |
| No known mutations found in 23S |  |  |  |  |

| **16S_rrsC** | | | | |
| --- | --- | --- | --- | --- |
| No known mutations found in 16S_rrsC |  |  |  |  |

| **16S_rrsH** | | | | |
| --- | --- | --- | --- | --- |
| No known mutations found in 16S_rrsH |  |  |  |  |

| **folP** | | | | |
| --- | --- | --- | --- | --- |
| No known mutations found in folP |  |  |  |  |

| **gyrB** | | | | |
| --- | --- | --- | --- | --- |
| No known mutations found in gyrB |  |  |  |  |

| **pmrB** | | | | |
| --- | --- | --- | --- | --- |
| No known mutations found in pmrB |  |  |  |  |

| **gyrA** | | | | |
| --- | --- | --- | --- | --- |
| **Mutation** | **Nucleotide change** | **Amino acid change** | **Resistance** | **PMID** |
| gyrA p.S83L | TCG ➝ TTG | S ➝ L | Nalidixic acid,Ciprofloxacin | [8891148](http://www.ncbi.nlm.nih.gov/pubmed/8891148) |
| gyrA p.D87N | GAC ➝ AAC | D ➝ N | Nalidixic acid,Ciprofloxacin | [12654733](http://www.ncbi.nlm.nih.gov/pubmed/12654733) |

| **16S_rrsB** | | | | |
| --- | --- | --- | --- | --- |
| No known mutations found in 16S_rrsB |  |  |  |  |

| **pmrA** | | | | |
| --- | --- | --- | --- | --- |
| No known mutations found in pmrA |  |  |  |  |

Top of Form

Bottom of Form

Top of Form

Bottom of Form

**CITATIONS**

For publication of results, please cite:


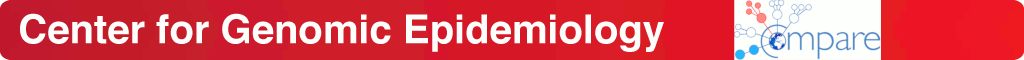


|  | Home | Services | Instructions | Output | Overview of genes | Article abstract |  |
| --- | --- | --- | --- | --- | --- | --- | --- |

**ResFinder-3.2 Server - Results**

**Input Files: *E057.fasta***

Show Acquired antimicrobial resistance results

**Acquired antimicrobial resistance gene - Results**

| **Tetracycline** | | | | | | |
| --- | --- | --- | --- | --- | --- | --- |
| **Resistance gene** | **Identity** | **Query / Template length** | **Contig** | **Position in contig** | **Predicted phenotype** | **Accession number** |
| tet(A) | 100 | 1200 / 1200 | NXIS01000072.1 Escherichia coli strain ST-665:E057 NODE_72_length_4135_cov_69.8994_ID_143, whole genome shotgun sequence | 1784..2983 | Tetracycline resistance | [AJ517790](http://www.ncbi.nlm.nih.gov/nuccore/AJ517790) |

| **Beta-lactam** | | | | | | |
| --- | --- | --- | --- | --- | --- | --- |
| **Resistance gene** | **Identity** | **Query / Template length** | **Contig** | **Position in contig** | **Predicted phenotype** | **Accession number** |
| blaCMY-2 | 100 | 1146 / 1146 | NXIS01000018.1 Escherichia coli strain ST-665:E057 NODE_18_length_94138_cov_167.593_ID_35, whole genome shotgun sequence | 79152..80297 | Beta-lactam resistance | [X91840](http://www.ncbi.nlm.nih.gov/nuccore/X91840) |

| **Macrolide** | | | | | | |
| --- | --- | --- | --- | --- | --- | --- |
| **Resistance gene** | **Identity** | **Query / Template length** | **Contig** | **Position in contig** | **Predicted phenotype** | **Accession number** |
| mdf(A) | 99.59 | 1233 / 1233 | NXIS01000014.1 Escherichia coli strain ST-665:E057 NODE_14_length_109477_cov_121.047_ID_27, whole genome shotgun sequence | 46470..47702 | Warning: gene is missing from Notes file. Please inform curator. | [Y08743](http://www.ncbi.nlm.nih.gov/nuccore/Y08743) |

Show Point mutation results

**Chromosomal point mutations - Results**

**Species:*escherichia_coli***

**Known Mutations**

| **pmrA** | | | | |
| --- | --- | --- | --- | --- |
| No mutations found in pmrA |  |  |  |  |

| **rpoB** | | | | |
| --- | --- | --- | --- | --- |
| No mutations found in rpoB |  |  |  |  |

| **23S** | | | | |
| --- | --- | --- | --- | --- |
| No known mutations found in 23S |  |  |  |  |

| **ampC** | | | | |
| --- | --- | --- | --- | --- |
| No mutations found in ampC |  |  |  |  |

| **16S_rrsH** | | | | |
| --- | --- | --- | --- | --- |
| No known mutations found in 16S_rrsH |  |  |  |  |

| **parC** | | | | |
| --- | --- | --- | --- | --- |
| No known mutations found in parC |  |  |  |  |

| **16S_rrsC** | | | | |
| --- | --- | --- | --- | --- |
| No known mutations found in 16S_rrsC |  |  |  |  |

| **pmrB** | | | | |
| --- | --- | --- | --- | --- |
| No mutations found in pmrB |  |  |  |  |

| **gyrA** | | | | |
| --- | --- | --- | --- | --- |
| No mutations found in gyrA |  |  |  |  |

| **parE** | | | | |
| --- | --- | --- | --- | --- |
| No mutations found in parE |  |  |  |  |

| **16S_rrsB** | | | | |
| --- | --- | --- | --- | --- |
| No known mutations found in 16S_rrsB |  |  |  |  |

| **folP** | | | | |
| --- | --- | --- | --- | --- |
| No known mutations found in folP |  |  |  |  |

| **gyrB** | | | | |
| --- | --- | --- | --- | --- |
| No mutations found in gyrB |  |  |  |  |

Top of Form

Bottom of Form

Top of Form

Bottom of Form

**CITATIONS**

For publication of results, please cite:


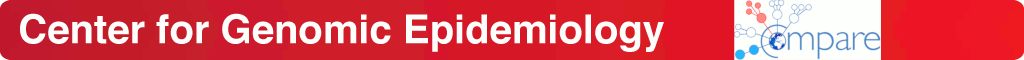


|  | Home | Services | Instructions | Output | Overview of genes | Article abstract |  |
| --- | --- | --- | --- | --- | --- | --- | --- |

**ResFinder-3.2 Server - Results**

**Input Files: *E060.fasta***

Show Acquired antimicrobial resistance results

**Acquired antimicrobial resistance gene - Results**

| **Fosfomycin** | | | | | | |
| --- | --- | --- | --- | --- | --- | --- |
| **Resistance gene** | **Identity** | **Query / Template length** | **Contig** | **Position in contig** | **Predicted phenotype** | **Accession number** |
| No hit found | | | | | | |

| **Macrolide** | | | | | | |
| --- | --- | --- | --- | --- | --- | --- |
| **Resistance gene** | **Identity** | **Query / Template length** | **Contig** | **Position in contig** | **Predicted phenotype** | **Accession number** |
| mdf(A) | 97.81 | 1233 / 1233 | NXLJ01000005.1 Escherichia coli strain ST-131:E060 NODE_5_length_252029_cov_54.9786_ID_9, whole genome shotgun sequence | 186274..187506 | Warning: gene is missing from Notes file. Please inform curator. | [Y08743](http://www.ncbi.nlm.nih.gov/nuccore/Y08743) |
| mph(A) | 100 | 906 / 906 | NXLJ01000043.1 Escherichia coli strain ST-131:E060 NODE_43_length_11279_cov_42.2442_ID_85, whole genome shotgun sequence | 10206..11111 | Macrolide resistance | [D16251](http://www.ncbi.nlm.nih.gov/nuccore/D16251) |

| **Quinolone** | | | | | | |
| --- | --- | --- | --- | --- | --- | --- |
| **Resistance gene** | **Identity** | **Query / Template length** | **Contig** | **Position in contig** | **Predicted phenotype** | **Accession number** |
| aac(6')-Ib-cr | 100 | 600 / 600 | NXLJ01000070.1 Escherichia coli strain ST-131:E060 NODE_70_length_2384_cov_47.1151_ID_139, whole genome shotgun sequence | 1640..2239 | Fluoroquinolone and aminoglycoside resistance | [DQ303918](http://www.ncbi.nlm.nih.gov/nuccore/DQ303918) |

| **Trimethoprim** | | | | | | |
| --- | --- | --- | --- | --- | --- | --- |
| **Resistance gene** | **Identity** | **Query / Template length** | **Contig** | **Position in contig** | **Predicted phenotype** | **Accession number** |
| dfrA17 | 100 | 474 / 474 | NXLJ01000043.1 Escherichia coli strain ST-131:E060 NODE_43_length_11279_cov_42.2442_ID_85, whole genome shotgun sequence | 2117..2590 | Trimethoprim resistance | [FJ460238](http://www.ncbi.nlm.nih.gov/nuccore/FJ460238) |

| **Sulphonamide** | | | | | | |
| --- | --- | --- | --- | --- | --- | --- |
| **Resistance gene** | **Identity** | **Query / Template length** | **Contig** | **Position in contig** | **Predicted phenotype** | **Accession number** |
| sul1 | 100 | 840 / 840 | NXLJ01000043.1 Escherichia coli strain ST-131:E060 NODE_43_length_11279_cov_42.2442_ID_85, whole genome shotgun sequence | 4056..4895 | Sulphonamide resistance | [U12338](http://www.ncbi.nlm.nih.gov/nuccore/U12338) |
| sul2 | 100 | 816 / 816 | NXLJ01000052.1 Escherichia coli strain ST-131:E060 NODE_52_length_6299_cov_188.108_ID_103, whole genome shotgun sequence | 4049..4864 | Sulphonamide resistance | [AY034138](http://www.ncbi.nlm.nih.gov/nuccore/AY034138) |

| **Aminoglycoside** | | | | | | |
| --- | --- | --- | --- | --- | --- | --- |
| **Resistance gene** | **Identity** | **Query / Template length** | **Contig** | **Position in contig** | **Predicted phenotype** | **Accession number** |
| aac(3)-IIa | 99.77 | 861 / 861 | NXLJ01000066.1 Escherichia coli strain ST-131:E060 NODE_66_length_2798_cov_32.7429_ID_131, whole genome shotgun sequence | 1796..2656 | Aminoglycoside resistance | [X51534](http://www.ncbi.nlm.nih.gov/nuccore/X51534) |
| aac(6')-Ib-cr | 100 | 600 / 600 | NXLJ01000070.1 Escherichia coli strain ST-131:E060 NODE_70_length_2384_cov_47.1151_ID_139, whole genome shotgun sequence | 1640..2239 | Fluoroquinolone and aminoglycoside resistance | [DQ303918](http://www.ncbi.nlm.nih.gov/nuccore/DQ303918) |
| aadA5 | 100 | 789 / 789 | NXLJ01000043.1 Escherichia coli strain ST-131:E060 NODE_43_length_11279_cov_42.2442_ID_85, whole genome shotgun sequence | 2721..3509 | Aminoglycoside resistance | [AF137361](http://www.ncbi.nlm.nih.gov/nuccore/AF137361) |
| aph(3'')-Ib | 100 | 804 / 804 | NXLJ01000052.1 Escherichia coli strain ST-131:E060 NODE_52_length_6299_cov_188.108_ID_103, whole genome shotgun sequence | 4925..5728 | Aminoglycoside resistance Alternate name; aph(3'')-Ib | [AF321551](http://www.ncbi.nlm.nih.gov/nuccore/AF321551) |
| aph(6)-Id | 100 | 572 / 837 | NXLJ01000052.1 Escherichia coli strain ST-131:E060 NODE_52_length_6299_cov_188.108_ID_103, whole genome shotgun sequence | 5728..6299 | Aminoglycoside resistance Alternate name; aph(6)-Id | [18676889](http://www.ncbi.nlm.nih.gov/nuccore/18676889) |
| aph(6)-Id | 100 | 572 / 837 | NXLJ01000052.1 Escherichia coli strain ST-131:E060 NODE_52_length_6299_cov_188.108_ID_103, whole genome shotgun sequence | 5728..6299 | Aminoglycoside resistance Alternate name; aph(6)-Id | [AF024602](http://www.ncbi.nlm.nih.gov/nuccore/AF024602) |
| aph(6)-Id | 100 | 572 / 837 | NXLJ01000052.1 Escherichia coli strain ST-131:E060 NODE_52_length_6299_cov_188.108_ID_103, whole genome shotgun sequence | 5728..6299 | Aminoglycoside resistance Alternate name; aph(6)-Id | [M28829](http://www.ncbi.nlm.nih.gov/nuccore/M28829) |

| **Phenicol** | | | | | | |
| --- | --- | --- | --- | --- | --- | --- |
| **Resistance gene** | **Identity** | **Query / Template length** | **Contig** | **Position in contig** | **Predicted phenotype** | **Accession number** |
| catB3 | 100 | 442 / 633 | NXLJ01000070.1 Escherichia coli strain ST-131:E060 NODE_70_length_2384_cov_47.1151_ID_139, whole genome shotgun sequence | 100..541 | Phenicol resistance | [AJ009818](http://www.ncbi.nlm.nih.gov/nuccore/AJ009818) |
| catB3 | 100 | 442 / 633 | NXLJ01000070.1 Escherichia coli strain ST-131:E060 NODE_70_length_2384_cov_47.1151_ID_139, whole genome shotgun sequence | 100..541 | Phenicol resistance | [U13880](http://www.ncbi.nlm.nih.gov/nuccore/U13880) |

| **Beta-lactam** | | | | | | |
| --- | --- | --- | --- | --- | --- | --- |
| **Resistance gene** | **Identity** | **Query / Template length** | **Contig** | **Position in contig** | **Predicted phenotype** | **Accession number** |
| blaCTX-M-15 | 100 | 876 / 876 | NXLJ01000039.1 Escherichia coli strain ST-131:E060 NODE_39_length_14861_cov_59.0868_ID_77, whole genome shotgun sequence | 11250..12125 | Beta-lactam resistance Alternate name; UOE-1 | [AY044436](http://www.ncbi.nlm.nih.gov/nuccore/AY044436) |
| blaOXA-1 | 100 | 831 / 831 | NXLJ01000070.1 Escherichia coli strain ST-131:E060 NODE_70_length_2384_cov_47.1151_ID_139, whole genome shotgun sequence | 679..1509 | Beta-lactam resistance | [HQ170510](http://www.ncbi.nlm.nih.gov/nuccore/HQ170510) |
| blaTEM-1B | 100 | 861 / 861 | NXLJ01000026.1 Escherichia coli strain ST-131:E060 NODE_26_length_42878_cov_83.7678_ID_51, whole genome shotgun sequence | 40380..41240 | Beta-lactam resistance Alternate name; RblaTEM-1 | [AY458016](http://www.ncbi.nlm.nih.gov/nuccore/AY458016) |

| **Tetracycline** | | | | | | |
| --- | --- | --- | --- | --- | --- | --- |
| **Resistance gene** | **Identity** | **Query / Template length** | **Contig** | **Position in contig** | **Predicted phenotype** | **Accession number** |
| tet(A) | 100 | 1200 / 1200 | NXLJ01000014.1 Escherichia coli strain ST-131:E060 NODE_14_length_148691_cov_49.7764_ID_27, whole genome shotgun sequence | 146782..147981 | Tetracycline resistance | [AJ517790](http://www.ncbi.nlm.nih.gov/nuccore/AJ517790) |

Show Point mutation results

**Chromosomal point mutations - Results**

**Species:*escherichia_coli***

**Known Mutations**

| **rpoB** | | | | |
| --- | --- | --- | --- | --- |
| No mutations found in rpoB |  |  |  |  |

| **pmrB** | | | | |
| --- | --- | --- | --- | --- |
| No known mutations found in pmrB |  |  |  |  |

| **pmrA** | | | | |
| --- | --- | --- | --- | --- |
| No known mutations found in pmrA |  |  |  |  |

| **gyrB** | | | | |
| --- | --- | --- | --- | --- |
| No known mutations found in gyrB |  |  |  |  |

| **23S** | | | | |
| --- | --- | --- | --- | --- |
| No known mutations found in 23S |  |  |  |  |

| **folP** | | | | |
| --- | --- | --- | --- | --- |
| No known mutations found in folP |  |  |  |  |

| **parE** | | | | |
| --- | --- | --- | --- | --- |
| **Mutation** | **Nucleotide change** | **Amino acid change** | **Resistance** | **PMID** |
| parE p.I529L | ATT ➝ CTT | I ➝ L | Nalidixic acid,Ciprofloxacin | [14506034](http://www.ncbi.nlm.nih.gov/pubmed/14506034) |

| **16S_rrsC** | | | | |
| --- | --- | --- | --- | --- |
| No known mutations found in 16S_rrsC |  |  |  |  |

| **gyrA** | | | | |
| --- | --- | --- | --- | --- |
| **Mutation** | **Nucleotide change** | **Amino acid change** | **Resistance** | **PMID** |
| gyrA p.S83L | TCG ➝ TTG | S ➝ L | Nalidixic acid,Ciprofloxacin | [8891148](http://www.ncbi.nlm.nih.gov/pubmed/8891148) |
| gyrA p.D87N | GAC ➝ AAC | D ➝ N | Nalidixic acid,Ciprofloxacin | [12654733](http://www.ncbi.nlm.nih.gov/pubmed/12654733) |

| **16S_rrsB** | | | | |
| --- | --- | --- | --- | --- |
| No known mutations found in 16S_rrsB |  |  |  |  |

| **16S_rrsH** | | | | |
| --- | --- | --- | --- | --- |
| No known mutations found in 16S_rrsH |  |  |  |  |

| **parC** | | | | |
| --- | --- | --- | --- | --- |
| **Mutation** | **Nucleotide change** | **Amino acid change** | **Resistance** | **PMID** |
| parC p.S80I | AGC ➝ ATT | S ➝ I | Nalidixic acid,Ciprofloxacin | [8851598](http://www.ncbi.nlm.nih.gov/pubmed/8851598) |
| parC p.E84V | GAA ➝ GTA | E ➝ V | Nalidixic acid,Ciprofloxacin | [12654733](http://www.ncbi.nlm.nih.gov/pubmed/12654733) |

| **ampC** | | | | |
| --- | --- | --- | --- | --- |
| No known mutations found in ampC |  |  |  |  |

Top of Form

Bottom of Form

Top of Form

Bottom of Form


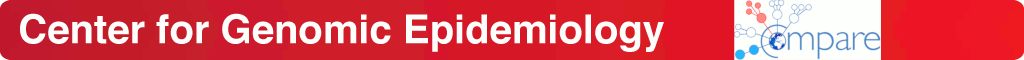


|  | Home | Services | Instructions | Output | Overview of genes | Article abstract |  |
| --- | --- | --- | --- | --- | --- | --- | --- |

**ResFinder-3.2 Server - Results**

**Input Files: *E062.fasta***

Show Acquired antimicrobial resistance results

**Acquired antimicrobial resistance gene - Results**

| **Quinolone** | | | | | | |
| --- | --- | --- | --- | --- | --- | --- |
| **Resistance gene** | **Identity** | **Query / Template length** | **Contig** | **Position in contig** | **Predicted phenotype** | **Accession number** |
| aac(6')-Ib-cr | 100 | 600 / 600 | NXJE01000083.1 Escherichia coli strain ST-131:E062 NODE_83_length_2296_cov_34.0013, whole genome shotgun sequence | 1596..2195 | Fluoroquinolone and aminoglycoside resistance | [DQ303918](http://www.ncbi.nlm.nih.gov/nuccore/DQ303918) |

| **Phenicol** | | | | | | |
| --- | --- | --- | --- | --- | --- | --- |
| **Resistance gene** | **Identity** | **Query / Template length** | **Contig** | **Position in contig** | **Predicted phenotype** | **Accession number** |
| catB3 | 100 | 442 / 633 | NXJE01000083.1 Escherichia coli strain ST-131:E062 NODE_83_length_2296_cov_34.0013, whole genome shotgun sequence | 56..497 | Phenicol resistance | [AJ009818](http://www.ncbi.nlm.nih.gov/nuccore/AJ009818) |
| catB3 | 100 | 442 / 633 | NXJE01000083.1 Escherichia coli strain ST-131:E062 NODE_83_length_2296_cov_34.0013, whole genome shotgun sequence | 56..497 | Phenicol resistance | [U13880](http://www.ncbi.nlm.nih.gov/nuccore/U13880) |

| **Aminoglycoside** | | | | | | |
| --- | --- | --- | --- | --- | --- | --- |
| **Resistance gene** | **Identity** | **Query / Template length** | **Contig** | **Position in contig** | **Predicted phenotype** | **Accession number** |
| aac(6')-Ib-cr | 100 | 600 / 600 | NXJE01000083.1 Escherichia coli strain ST-131:E062 NODE_83_length_2296_cov_34.0013, whole genome shotgun sequence | 1596..2195 | Fluoroquinolone and aminoglycoside resistance | [DQ303918](http://www.ncbi.nlm.nih.gov/nuccore/DQ303918) |

| **Tetracycline** | | | | | | |
| --- | --- | --- | --- | --- | --- | --- |
| **Resistance gene** | **Identity** | **Query / Template length** | **Contig** | **Position in contig** | **Predicted phenotype** | **Accession number** |
| tet(A) | 100 | 1200 / 1200 | NXJE01000052.1 Escherichia coli strain ST-131:E062 NODE_52_length_10140_cov_30.8601, whole genome shotgun sequence | 943..2142 | Tetracycline resistance | [AJ517790](http://www.ncbi.nlm.nih.gov/nuccore/AJ517790) |

| **Macrolide** | | | | | | |
| --- | --- | --- | --- | --- | --- | --- |
| **Resistance gene** | **Identity** | **Query / Template length** | **Contig** | **Position in contig** | **Predicted phenotype** | **Accession number** |
| mdf(A) | 97.81 | 1233 / 1233 | NXJE01000010.1 Escherichia coli strain ST-131:E062 NODE_10_length_165057_cov_34.0438, whole genome shotgun sequence | 64141..65373 | Warning: gene is missing from Notes file. Please inform curator. | [Y08743](http://www.ncbi.nlm.nih.gov/nuccore/Y08743) |

| **Beta-lactam** | | | | | | |
| --- | --- | --- | --- | --- | --- | --- |
| **Resistance gene** | **Identity** | **Query / Template length** | **Contig** | **Position in contig** | **Predicted phenotype** | **Accession number** |
| blaCTX-M-15 | 100 | 876 / 876 | NXJE01000070.1 Escherichia coli strain ST-131:E062 NODE_70_length_4202_cov_38.387, whole genome shotgun sequence | 1981..2856 | Beta-lactam resistance Alternate name; UOE-1 | [AY044436](http://www.ncbi.nlm.nih.gov/nuccore/AY044436) |
| blaOXA-1 | 100 | 831 / 831 | NXJE01000083.1 Escherichia coli strain ST-131:E062 NODE_83_length_2296_cov_34.0013, whole genome shotgun sequence | 635..1465 | Beta-lactam resistance | [HQ170510](http://www.ncbi.nlm.nih.gov/nuccore/HQ170510) |

| **Colistin** |
| --- |

Show Point mutation results

**Chromosomal point mutations - Results**

**Species:*escherichia_coli***

**Known Mutations**

| **parE** | | | | |
| --- | --- | --- | --- | --- |
| **Mutation** | **Nucleotide change** | **Amino acid change** | **Resistance** | **PMID** |
| parE p.I529L | ATT ➝ CTT | I ➝ L | Nalidixic acid,Ciprofloxacin | [14506034](http://www.ncbi.nlm.nih.gov/pubmed/14506034) |

| **16S_rrsC** | | | | |
| --- | --- | --- | --- | --- |
| No known mutations found in 16S_rrsC |  |  |  |  |

| **16S_rrsB** | | | | |
| --- | --- | --- | --- | --- |
| No known mutations found in 16S_rrsB |  |  |  |  |

| **folP** | | | | |
| --- | --- | --- | --- | --- |
| No known mutations found in folP |  |  |  |  |

| **pmrA** | | | | |
| --- | --- | --- | --- | --- |
| No known mutations found in pmrA |  |  |  |  |

| **ampC** | | | | |
| --- | --- | --- | --- | --- |
| No known mutations found in ampC |  |  |  |  |

| **parC** | | | | |
| --- | --- | --- | --- | --- |
| **Mutation** | **Nucleotide change** | **Amino acid change** | **Resistance** | **PMID** |
| parC p.S80I | AGC ➝ ATT | S ➝ I | Nalidixic acid,Ciprofloxacin | [8851598](http://www.ncbi.nlm.nih.gov/pubmed/8851598) |
| parC p.E84V | GAA ➝ GTA | E ➝ V | Nalidixic acid,Ciprofloxacin | [12654733](http://www.ncbi.nlm.nih.gov/pubmed/12654733) |

| **pmrB** | | | | |
| --- | --- | --- | --- | --- |
| No known mutations found in pmrB |  |  |  |  |

| **gyrA** | | | | |
| --- | --- | --- | --- | --- |
| **Mutation** | **Nucleotide change** | **Amino acid change** | **Resistance** | **PMID** |
| gyrA p.S83L | TCG ➝ TTG | S ➝ L | Nalidixic acid,Ciprofloxacin | [8891148](http://www.ncbi.nlm.nih.gov/pubmed/8891148) |
| gyrA p.D87N | GAC ➝ AAC | D ➝ N | Nalidixic acid,Ciprofloxacin | [12654733](http://www.ncbi.nlm.nih.gov/pubmed/12654733) |

| **23S** | | | | |
| --- | --- | --- | --- | --- |
| No known mutations found in 23S |  |  |  |  |

| **gyrB** | | | | |
| --- | --- | --- | --- | --- |
| No known mutations found in gyrB |  |  |  |  |

| **rpoB** | | | | |
| --- | --- | --- | --- | --- |
| No mutations found in rpoB |  |  |  |  |

| **16S_rrsH** | | | | |
| --- | --- | --- | --- | --- |
| No known mutations found in 16S_rrsH |  |  |  |  |

Top of Form

Bottom of Form

Top of Form

Bottom of Form

**CITATIONS**


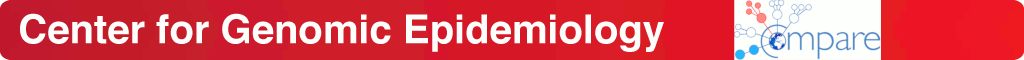


|  | Home | Services | Instructions | Output | Overview of genes | Article abstract |  |
| --- | --- | --- | --- | --- | --- | --- | --- |

**ResFinder-3.2 Server - Results**

**Input Files: *E062.fasta***

Show Acquired antimicrobial resistance results

**Acquired antimicrobial resistance gene - Results**

| **Fusidicacid** | | | | | | |
| --- | --- | --- | --- | --- | --- | --- |
| **Resistance gene** | **Identity** | **Query / Template length** | **Contig** | **Position in contig** | **Predicted phenotype** | **Accession number** |
| No hit found | | | | | | |

| **Trimethoprim** | | | | | | |
| --- | --- | --- | --- | --- | --- | --- |
| **Resistance gene** | **Identity** | **Query / Template length** | **Contig** | **Position in contig** | **Predicted phenotype** | **Accession number** |
| No hit found | | | | | | |

| **Nitroimidazole** | | | | | | |
| --- | --- | --- | --- | --- | --- | --- |
| **Resistance gene** | **Identity** | **Query / Template length** | **Contig** | **Position in contig** | **Predicted phenotype** | **Accession number** |
| No hit found | | | | | | |

| **Quinolone** | | | | | | |
| --- | --- | --- | --- | --- | --- | --- |
| **Resistance gene** | **Identity** | **Query / Template length** | **Contig** | **Position in contig** | **Predicted phenotype** | **Accession number** |
| aac(6')-Ib-cr | 100 | 600 / 600 | NXJE01000083.1 Escherichia coli strain ST-131:E062 NODE_83_length_2296_cov_34.0013, whole genome shotgun sequence | 1596..2195 | Fluoroquinolone and aminoglycoside resistance | [DQ303918](http://www.ncbi.nlm.nih.gov/nuccore/DQ303918) |

| **Aminoglycoside** | | | | | | |
| --- | --- | --- | --- | --- | --- | --- |
| **Resistance gene** | **Identity** | **Query / Template length** | **Contig** | **Position in contig** | **Predicted phenotype** | **Accession number** |
| aac(6')-Ib-cr | 100 | 600 / 600 | NXJE01000083.1 Escherichia coli strain ST-131:E062 NODE_83_length_2296_cov_34.0013, whole genome shotgun sequence | 1596..2195 | Fluoroquinolone and aminoglycoside resistance | [DQ303918](http://www.ncbi.nlm.nih.gov/nuccore/DQ303918) |

| **Oxazolidinone** | | | | | | |
| --- | --- | --- | --- | --- | --- | --- |
| **Resistance gene** | **Identity** | **Query / Template length** | **Contig** | **Position in contig** | **Predicted phenotype** | **Accession number** |
| No hit found | | | | | | |

| **Phenicol** | | | | | | |
| --- | --- | --- | --- | --- | --- | --- |
| **Resistance gene** | **Identity** | **Query / Template length** | **Contig** | **Position in contig** | **Predicted phenotype** | **Accession number** |
| catB3 | 100 | 442 / 633 | NXJE01000083.1 Escherichia coli strain ST-131:E062 NODE_83_length_2296_cov_34.0013, whole genome shotgun sequence | 56..497 | Phenicol resistance | [AJ009818](http://www.ncbi.nlm.nih.gov/nuccore/AJ009818) |
| catB3 | 100 | 442 / 633 | NXJE01000083.1 Escherichia coli strain ST-131:E062 NODE_83_length_2296_cov_34.0013, whole genome shotgun sequence | 56..497 | Phenicol resistance | [U13880](http://www.ncbi.nlm.nih.gov/nuccore/U13880) |

| **Glycopeptide** | | | | | | |
| --- | --- | --- | --- | --- | --- | --- |
| **Resistance gene** | **Identity** | **Query / Template length** | **Contig** | **Position in contig** | **Predicted phenotype** | **Accession number** |
| No hit found | | | | | | |

| **Tetracycline** | | | | | | |
| --- | --- | --- | --- | --- | --- | --- |
| **Resistance gene** | **Identity** | **Query / Template length** | **Contig** | **Position in contig** | **Predicted phenotype** | **Accession number** |
| tet(A) | 100 | 1200 / 1200 | NXJE01000052.1 Escherichia coli strain ST-131:E062 NODE_52_length_10140_cov_30.8601, whole genome shotgun sequence | 943..2142 | Tetracycline resistance | [AJ517790](http://www.ncbi.nlm.nih.gov/nuccore/AJ517790) |

| **Macrolide** | | | | | | |
| --- | --- | --- | --- | --- | --- | --- |
| **Resistance gene** | **Identity** | **Query / Template length** | **Contig** | **Position in contig** | **Predicted phenotype** | **Accession number** |
| mdf(A) | 97.81 | 1233 / 1233 | NXJE01000010.1 Escherichia coli strain ST-131:E062 NODE_10_length_165057_cov_34.0438, whole genome shotgun sequence | 64141..65373 | Warning: gene is missing from Notes file. Please inform curator. | [Y08743](http://www.ncbi.nlm.nih.gov/nuccore/Y08743) |

| **Colistin** | | | | | | |
| --- | --- | --- | --- | --- | --- | --- |
| **Resistance gene** | **Identity** | **Query / Template length** | **Contig** | **Position in contig** | **Predicted phenotype** | **Accession number** |
| No hit found | | | | | | |

| **Beta-lactam** | | | | | | |
| --- | --- | --- | --- | --- | --- | --- |
| **Resistance gene** | **Identity** | **Query / Template length** | **Contig** | **Position in contig** | **Predicted phenotype** | **Accession number** |
| blaCTX-M-15 | 100 | 876 / 876 | NXJE01000070.1 Escherichia coli strain ST-131:E062 NODE_70_length_4202_cov_38.387, whole genome shotgun sequence | 1981..2856 | Beta-lactam resistance Alternate name; UOE-1 | [AY044436](http://www.ncbi.nlm.nih.gov/nuccore/AY044436) |
| blaOXA-1 | 100 | 831 / 831 | NXJE01000083.1 Escherichia coli strain ST-131:E062 NODE_83_length_2296_cov_34.0013, whole genome shotgun sequence | 635..1465 | Beta-lactam resistance | [HQ170510](http://www.ncbi.nlm.nih.gov/nuccore/HQ170510) |

| **Fosfomycin** | | | | | | |
| --- | --- | --- | --- | --- | --- | --- |
| **Resistance gene** | **Identity** | **Query / Template length** | **Contig** | **Position in contig** | **Predicted phenotype** | **Accession number** |
| No hit found | | | | | | |

| **Sulphonamide** | | | | | | |
| --- | --- | --- | --- | --- | --- | --- |
| **Resistance gene** | **Identity** | **Query / Template length** | **Contig** | **Position in contig** | **Predicted phenotype** | **Accession number** |
| No hit found | | | | | | |

| **Rifampicin** | | | | | | |
| --- | --- | --- | --- | --- | --- | --- |
| **Resistance gene** | **Identity** | **Query / Template length** | **Contig** | **Position in contig** | **Predicted phenotype** | **Accession number** |
| No hit found | | | | | | |

extended output

**Selected %ID threshold:  *90 %***

**Selected minimum length:  *60 %***

Top of Form

Bottom of Form

Top of Form

Bottom of Form

Top of Form

Bottom of Form

Top of Form

Bottom of Form

Show Point mutation results

**Chromosomal point mutations - Results**

**Species:*escherichia_coli***

**Known Mutations**

| **pmrB** | | | | |
| --- | --- | --- | --- | --- |
| No known mutations found in pmrB |  |  |  |  |

| **16S_rrsB** | | | | |
| --- | --- | --- | --- | --- |
| No known mutations found in 16S_rrsB |  |  |  |  |

| **gyrA** | | | | |
| --- | --- | --- | --- | --- |
| **Mutation** | **Nucleotide change** | **Amino acid change** | **Resistance** | **PMID** |
| gyrA p.S83L | TCG ➝ TTG | S ➝ L | Nalidixic acid,Ciprofloxacin | [8891148](http://www.ncbi.nlm.nih.gov/pubmed/8891148) |
| gyrA p.D87N | GAC ➝ AAC | D ➝ N | Nalidixic acid,Ciprofloxacin | [12654733](http://www.ncbi.nlm.nih.gov/pubmed/12654733) |

| **16S_rrsH** | | | | |
| --- | --- | --- | --- | --- |
| No known mutations found in 16S_rrsH |  |  |  |  |

| **folP** | | | | |
| --- | --- | --- | --- | --- |
| No known mutations found in folP |  |  |  |  |

| **16S_rrsC** | | | | |
| --- | --- | --- | --- | --- |
| No known mutations found in 16S_rrsC |  |  |  |  |

| **pmrA** | | | | |
| --- | --- | --- | --- | --- |
| No known mutations found in pmrA |  |  |  |  |

| **parE** | | | | |
| --- | --- | --- | --- | --- |
| **Mutation** | **Nucleotide change** | **Amino acid change** | **Resistance** | **PMID** |
| parE p.I529L | ATT ➝ CTT | I ➝ L | Nalidixic acid,Ciprofloxacin | [14506034](http://www.ncbi.nlm.nih.gov/pubmed/14506034) |

| **parC** | | | | |
| --- | --- | --- | --- | --- |
| **Mutation** | **Nucleotide change** | **Amino acid change** | **Resistance** | **PMID** |
| parC p.S80I | AGC ➝ ATT | S ➝ I | Nalidixic acid,Ciprofloxacin | [8851598](http://www.ncbi.nlm.nih.gov/pubmed/8851598) |
| parC p.E84V | GAA ➝ GTA | E ➝ V | Nalidixic acid,Ciprofloxacin | [12654733](http://www.ncbi.nlm.nih.gov/pubmed/12654733) |

| **23S** | | | | |
| --- | --- | --- | --- | --- |
| No known mutations found in 23S |  |  |  |  |

| **gyrB** | | | | |
| --- | --- | --- | --- | --- |
| No known mutations found in gyrB |  |  |  |  |

| **ampC** | | | | |
| --- | --- | --- | --- | --- |
| No known mutations found in ampC |  |  |  |  |

| **rpoB** | | | | |
| --- | --- | --- | --- | --- |
| No mutations found in rpoB |  |  |  |  |

Top of Form

Bottom of Form

Top of Form

Bottom of Form

**CITATIONS**

For publication of results, please cite:


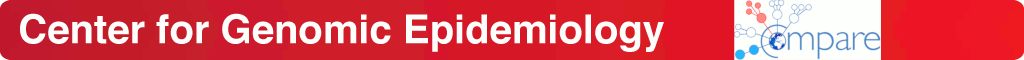


|  | Home | Services | Instructions | Output | Overview of genes | Article abstract |  |
| --- | --- | --- | --- | --- | --- | --- | --- |

**ResFinder-3.2 Server - Results**

**Input Files: *K011.fasta***

Show Acquired antimicrobial resistance results

**Acquired antimicrobial resistance gene - Results**

| **Rifampicin** | | | | | | |
| --- | --- | --- | --- | --- | --- | --- |
| **Resistance gene** | **Identity** | **Query / Template length** | **Contig** | **Position in contig** | **Predicted phenotype** | **Accession number** |
| ARR-2 | 100 | 453 / 453 | NXKS01000052.1 Escherichia coli strain ST-410:K011 NODE_52_length_16301_cov_51.8803_ID_103, whole genome shotgun sequence | 1068..1520 | Rifampicin resistance | [HQ141279](http://www.ncbi.nlm.nih.gov/nuccore/HQ141279) |

| **Quinolone** | | | | | | |
| --- | --- | --- | --- | --- | --- | --- |
| **Resistance gene** | **Identity** | **Query / Template length** | **Contig** | **Position in contig** | **Predicted phenotype** | **Accession number** |
| aac(6')-Ib-cr | 100 | 600 / 600 | NXKS01000086.1 Escherichia coli strain ST-410:K011 NODE_86_length_2381_cov_148.955_ID_171, whole genome shotgun sequence | 1640..2239 | Fluoroquinolone and aminoglycoside resistance | [DQ303918](http://www.ncbi.nlm.nih.gov/nuccore/DQ303918) |

| **Phenicol** | | | | | | |
| --- | --- | --- | --- | --- | --- | --- |
| **Resistance gene** | **Identity** | **Query / Template length** | **Contig** | **Position in contig** | **Predicted phenotype** | **Accession number** |
| catB3 | 100 | 442 / 633 | NXKS01000086.1 Escherichia coli strain ST-410:K011 NODE_86_length_2381_cov_148.955_ID_171, whole genome shotgun sequence | 100..541 | Phenicol resistance | [AJ009818](http://www.ncbi.nlm.nih.gov/nuccore/AJ009818) |
| catB3 | 100 | 442 / 633 | NXKS01000086.1 Escherichia coli strain ST-410:K011 NODE_86_length_2381_cov_148.955_ID_171, whole genome shotgun sequence | 100..541 | Phenicol resistance | [U13880](http://www.ncbi.nlm.nih.gov/nuccore/U13880) |
| cmlA1 | 99.68 | 1260 / 1260 | NXKS01000052.1 Escherichia coli strain ST-410:K011 NODE_52_length_16301_cov_51.8803_ID_103, whole genome shotgun sequence | 1841..3100 | Phenicol resistance | [M64556](http://www.ncbi.nlm.nih.gov/nuccore/M64556) |
| floR | 98.35 | 1214 / 1215 | NXKS01000071.1 Escherichia coli strain ST-410:K011 NODE_71_length_6114_cov_21.4545_ID_141, whole genome shotgun sequence | 4475..5688 | Phenicol resistance | [AF118107](http://www.ncbi.nlm.nih.gov/nuccore/AF118107) |

| **Trimethoprim** | | | | | | |
| --- | --- | --- | --- | --- | --- | --- |
| **Resistance gene** | **Identity** | **Query / Template length** | **Contig** | **Position in contig** | **Predicted phenotype** | **Accession number** |
| dfrA23 | 100 | 561 / 561 | NXKS01000052.1 Escherichia coli strain ST-410:K011 NODE_52_length_16301_cov_51.8803_ID_103, whole genome shotgun sequence | 9415..9975 | Trimethoprim resistance | [AJ746361](http://www.ncbi.nlm.nih.gov/nuccore/AJ746361) |

| **Beta-lactam** | | | | | | |
| --- | --- | --- | --- | --- | --- | --- |
| **Resistance gene** | **Identity** | **Query / Template length** | **Contig** | **Position in contig** | **Predicted phenotype** | **Accession number** |
| blaCTX-M-15 | 100 | 876 / 876 | NXKS01000078.1 Escherichia coli strain ST-410:K011 NODE_78_length_4254_cov_145.597_ID_155, whole genome shotgun sequence | 644..1519 | Beta-lactam resistance Alternate name; UOE-1 | [AY044436](http://www.ncbi.nlm.nih.gov/nuccore/AY044436) |
| blaOXA-1 | 100 | 831 / 831 | NXKS01000086.1 Escherichia coli strain ST-410:K011 NODE_86_length_2381_cov_148.955_ID_171, whole genome shotgun sequence | 679..1509 | Beta-lactam resistance | [HQ170510](http://www.ncbi.nlm.nih.gov/nuccore/HQ170510) |
| blaOXA-10 | 100 | 801 / 801 | NXKS01000052.1 Escherichia coli strain ST-410:K011 NODE_52_length_16301_cov_51.8803_ID_103, whole genome shotgun sequence | 3365..4165 | Beta-lactam resistance Alternate name; PSE-2 | [J03427](http://www.ncbi.nlm.nih.gov/nuccore/J03427) |

| **Macrolide** | | | | | | |
| --- | --- | --- | --- | --- | --- | --- |
| **Resistance gene** | **Identity** | **Query / Template length** | **Contig** | **Position in contig** | **Predicted phenotype** | **Accession number** |
| mdf(A) | 98.78 | 1233 / 1233 | NXKS01000010.1 Escherichia coli strain ST-410:K011 NODE_10_length_140324_cov_46.8878_ID_19, whole genome shotgun sequence | 63075..64307 | Warning: gene is missing from Notes file. Please inform curator. | [Y08743](http://www.ncbi.nlm.nih.gov/nuccore/Y08743) |
| mph(A) | 100 | 906 / 906 | NXKS01000076.1 Escherichia coli strain ST-410:K011 NODE_76_length_4337_cov_42.5559_ID_151, whole genome shotgun sequence | 3264..4169 | Macrolide resistance | [D16251](http://www.ncbi.nlm.nih.gov/nuccore/D16251) |

| **Sulphonamide** | | | | | | |
| --- | --- | --- | --- | --- | --- | --- |
| **Resistance gene** | **Identity** | **Query / Template length** | **Contig** | **Position in contig** | **Predicted phenotype** | **Accession number** |
| sul1 | 100 | 840 / 840 | NXKS01000052.1 Escherichia coli strain ST-410:K011 NODE_52_length_16301_cov_51.8803_ID_103, whole genome shotgun sequence | 5438..6277 | Sulphonamide resistance | [U12338](http://www.ncbi.nlm.nih.gov/nuccore/U12338) |
| sul2 | 100 | 816 / 816 | NXKS01000019.1 Escherichia coli strain ST-410:K011 NODE_19_length_88471_cov_71.4106_ID_37, whole genome shotgun sequence | 2070..2885 | Sulphonamide resistance | [AY034138](http://www.ncbi.nlm.nih.gov/nuccore/AY034138) |

| **Aminoglycoside** | | | | | | |
| --- | --- | --- | --- | --- | --- | --- |
| **Resistance gene** | **Identity** | **Query / Template length** | **Contig** | **Position in contig** | **Predicted phenotype** | **Accession number** |
| aac(3)-IIa | 99.77 | 861 / 861 | NXKS01000084.1 Escherichia coli strain ST-410:K011 NODE_84_length_2798_cov_154.955_ID_167, whole genome shotgun sequence | 1796..2656 | Aminoglycoside resistance | [X51534](http://www.ncbi.nlm.nih.gov/nuccore/X51534) |
| aac(6')-Ib-cr | 100 | 600 / 600 | NXKS01000086.1 Escherichia coli strain ST-410:K011 NODE_86_length_2381_cov_148.955_ID_171, whole genome shotgun sequence | 1640..2239 | Fluoroquinolone and aminoglycoside resistance | [DQ303918](http://www.ncbi.nlm.nih.gov/nuccore/DQ303918) |
| aadA1 | 99.75 | 792 / 792 | NXKS01000052.1 Escherichia coli strain ST-410:K011 NODE_52_length_16301_cov_51.8803_ID_103, whole genome shotgun sequence | 4182..4973 | Aminoglycoside resistance | [JQ414041](http://www.ncbi.nlm.nih.gov/nuccore/JQ414041) |
| aadA1 | 99.75 | 792 / 792 | NXKS01000052.1 Escherichia coli strain ST-410:K011 NODE_52_length_16301_cov_51.8803_ID_103, whole genome shotgun sequence | 4182..4973 | Aminoglycoside resistance | [JX185132](http://www.ncbi.nlm.nih.gov/nuccore/JX185132) |
| aph(3'')-Ib | 100 | 804 / 804 | NXKS01000019.1 Escherichia coli strain ST-410:K011 NODE_19_length_88471_cov_71.4106_ID_37, whole genome shotgun sequence | 1206..2009 | Aminoglycoside resistance Alternate name; aph(3'')-Ib | [AF321551](http://www.ncbi.nlm.nih.gov/nuccore/AF321551) |
| aph(6)-Id | 100 | 837 / 837 | NXKS01000019.1 Escherichia coli strain ST-410:K011 NODE_19_length_88471_cov_71.4106_ID_37, whole genome shotgun sequence | 370..1206 | Aminoglycoside resistance Alternate name; aph(6)-Id | [M28829](http://www.ncbi.nlm.nih.gov/nuccore/M28829) |

| **Tetracycline** | | | | | | |
| --- | --- | --- | --- | --- | --- | --- |
| **Resistance gene** | **Identity** | **Query / Template length** | **Contig** | **Position in contig** | **Predicted phenotype** | **Accession number** |
| tet(A) | 100 | 1200 / 1200 | NXKS01000080.1 Escherichia coli strain ST-410:K011 NODE_80_length_3950_cov_38.9055_ID_159, whole genome shotgun sequence | 1784..2983 | Tetracycline resistance | [AJ517790](http://www.ncbi.nlm.nih.gov/nuccore/AJ517790) |

extended output

Show Point mutation results

**Chromosomal point mutations - Results**

**Species:*escherichia_coli***

**Known Mutations**

| **gyrA** | | | | |
| --- | --- | --- | --- | --- |
| **Mutation** | **Nucleotide change** | **Amino acid change** | **Resistance** | **PMID** |
| gyrA p.S83L | TCG ➝ TTG | S ➝ L | Nalidixic acid,Ciprofloxacin | [8891148](http://www.ncbi.nlm.nih.gov/pubmed/8891148) |
| gyrA p.D87N | GAC ➝ AAC | D ➝ N | Nalidixic acid,Ciprofloxacin | [12654733](http://www.ncbi.nlm.nih.gov/pubmed/12654733) |

| **parC** | | | | |
| --- | --- | --- | --- | --- |
| **Mutation** | **Nucleotide change** | **Amino acid change** | **Resistance** | **PMID** |
| parC p.S80I | AGC ➝ ATC | S ➝ I | Nalidixic acid,Ciprofloxacin | [8851598](http://www.ncbi.nlm.nih.gov/pubmed/8851598) |

| **16S_rrsB** | | | | |
| --- | --- | --- | --- | --- |
| No known mutations found in 16S_rrsB |  |  |  |  |

| **pmrB** | | | | |
| --- | --- | --- | --- | --- |
| No known mutations found in pmrB |  |  |  |  |

| **23S** | | | | |
| --- | --- | --- | --- | --- |
| No known mutations found in 23S |  |  |  |  |

| **rpoB** | | | | |
| --- | --- | --- | --- | --- |
| No known mutations found in rpoB |  |  |  |  |

| **ampC** | | | | |
| --- | --- | --- | --- | --- |
| No mutations found in ampC |  |  |  |  |

| **folP** | | | | |
| --- | --- | --- | --- | --- |
| No mutations found in folP |  |  |  |  |

| **16S_rrsC** | | | | |
| --- | --- | --- | --- | --- |
| No known mutations found in 16S_rrsC |  |  |  |  |

| **pmrA** | | | | |
| --- | --- | --- | --- | --- |
| No known mutations found in pmrA |  |  |  |  |

| **parE** | | | | |
| --- | --- | --- | --- | --- |
| **Mutation** | **Nucleotide change** | **Amino acid change** | **Resistance** | **PMID** |
| parE p.S458A | TCG ➝ GCG | S ➝ A | Nalidixic acid,Ciprofloxacin | [28598203](http://www.ncbi.nlm.nih.gov/pubmed/28598203) |

| **gyrB** | | | | |
| --- | --- | --- | --- | --- |
| No mutations found in gyrB |  |  |  |  |

| **16S_rrsH** | | | | |
| --- | --- | --- | --- | --- |
| No known mutations found in 16S_rrsH |  |  |  |  |

Top of Form

Bottom of Form

Top of Form

Bottom of Form

**CITATIONS**

For publication of results, please cite:


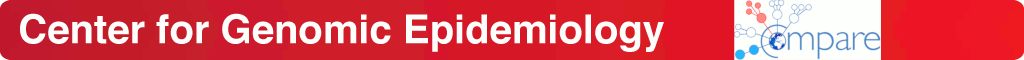


|  | Home | Services | Instructions | Output | Overview of genes | Article abstract |  |
| --- | --- | --- | --- | --- | --- | --- | --- |

**ResFinder-3.2 Server - Results**

**Input Files: *E063.fasta***

Show Acquired antimicrobial resistance results

**Acquired antimicrobial resistance gene - Results**

| **Sulphonamide** | | | | | | |
| --- | --- | --- | --- | --- | --- | --- |
| **Resistance gene** | **Identity** | **Query / Template length** | **Contig** | **Position in contig** | **Predicted phenotype** | **Accession number** |
| sul2 | 100 | 816 / 816 | NXIT01000025.1 Escherichia coli strain ST-131:E063 NODE_25_length_87986_cov_109.647_ID_49, whole genome shotgun sequence | 12934..13749 | Sulphonamide resistance | [AY034138](http://www.ncbi.nlm.nih.gov/nuccore/AY034138) |

| **Aminoglycoside** | | | | | | |
| --- | --- | --- | --- | --- | --- | --- |
| **Resistance gene** | **Identity** | **Query / Template length** | **Contig** | **Position in contig** | **Predicted phenotype** | **Accession number** |
| aph(3'')-Ib | 100 | 529 / 804 | NXIT01000025.1 Escherichia coli strain ST-131:E063 NODE_25_length_87986_cov_109.647_ID_49, whole genome shotgun sequence | 12345..12873 | Aminoglycoside resistance Alternate name; aph(3'')-Ib | [AF321551](http://www.ncbi.nlm.nih.gov/nuccore/AF321551) |
| aph(6)-Id | 100 | 837 / 837 | NXIT01000025.1 Escherichia coli strain ST-131:E063 NODE_25_length_87986_cov_109.647_ID_49, whole genome shotgun sequence | 10666..11502 | Aminoglycoside resistance Alternate name; aph(6)-Id | [M28829](http://www.ncbi.nlm.nih.gov/nuccore/M28829) |

| **Macrolide** | | | | | | |
| --- | --- | --- | --- | --- | --- | --- |
| **Resistance gene** | **Identity** | **Query / Template length** | **Contig** | **Position in contig** | **Predicted phenotype** | **Accession number** |
| mdf(A) | 97.73 | 1233 / 1233 | NXIT01000013.1 Escherichia coli strain ST-131:E063 NODE_13_length_151395_cov_99.5836_ID_25, whole genome shotgun sequence | 99762..100994 | Warning: gene is missing from Notes file. Please inform curator. | [Y08743](http://www.ncbi.nlm.nih.gov/nuccore/Y08743) |

| **Beta-lactam** | | | | | | |
| --- | --- | --- | --- | --- | --- | --- |
| **Resistance gene** | **Identity** | **Query / Template length** | **Contig** | **Position in contig** | **Predicted phenotype** | **Accession number** |
| blaCTX-M-15 | 100 | 876 / 876 | NXIT01000025.1 Escherichia coli strain ST-131:E063 NODE_25_length_87986_cov_109.647_ID_49, whole genome shotgun sequence | 25943..26818 | Beta-lactam resistance Alternate name; UOE-1 | [AY044436](http://www.ncbi.nlm.nih.gov/nuccore/AY044436) |
| blaTEM-1B | 100 | 861 / 861 | NXIT01000025.1 Escherichia coli strain ST-131:E063 NODE_25_length_87986_cov_109.647_ID_49, whole genome shotgun sequence | 22261..23121 | Beta-lactam resistance Alternate name; RblaTEM-1 | [AY458016](http://www.ncbi.nlm.nih.gov/nuccore/AY458016) |

| **Trimethoprim** | | | | | | |
| --- | --- | --- | --- | --- | --- | --- |
| **Resistance gene** | **Identity** | **Query / Template length** | **Contig** | **Position in contig** | **Predicted phenotype** | **Accession number** |
| dfrA14 | 100 | 474 / 474 | NXIT01000025.1 Escherichia coli strain ST-131:E063 NODE_25_length_87986_cov_109.647_ID_49, whole genome shotgun sequence | 11852..12325 | Trimethoprim resistance | [AF393510](http://www.ncbi.nlm.nih.gov/nuccore/AF393510) |

| **Tetracycline** | | | | | | |
| --- | --- | --- | --- | --- | --- | --- |
| **Resistance gene** | **Identity** | **Query / Template length** | **Contig** | **Position in contig** | **Predicted phenotype** | **Accession number** |
| tet(A) | 100 | 1200 / 1200 | NXIT01000072.1 Escherichia coli strain ST-131:E063 NODE_72_length_5248_cov_66.1651_ID_143, whole genome shotgun sequence | 1784..2983 | Tetracycline resistance | [AJ517790](http://www.ncbi.nlm.nih.gov/nuccore/AJ517790) |

Show Point mutation results

**Chromosomal point mutations - Results**

**Species:*escherichia_coli***

**Known Mutations**

| **pmrA** | | | | |
| --- | --- | --- | --- | --- |
| No known mutations found in pmrA |  |  |  |  |

| **16S_rrsH** | | | | |
| --- | --- | --- | --- | --- |
| No known mutations found in 16S_rrsH |  |  |  |  |

| **folP** | | | | |
| --- | --- | --- | --- | --- |
| No known mutations found in folP |  |  |  |  |

| **parE** | | | | |
| --- | --- | --- | --- | --- |
| **Mutation** | **Nucleotide change** | **Amino acid change** | **Resistance** | **PMID** |
| parE p.I529L | ATT ➝ CTT | I ➝ L | Nalidixic acid,Ciprofloxacin | [14506034](http://www.ncbi.nlm.nih.gov/pubmed/14506034) |

| **pmrB** | | | | |
| --- | --- | --- | --- | --- |
| No known mutations found in pmrB |  |  |  |  |

| **16S_rrsB** | | | | |
| --- | --- | --- | --- | --- |
| No known mutations found in 16S_rrsB |  |  |  |  |

| **rpoB** | | | | |
| --- | --- | --- | --- | --- |
| No known mutations found in rpoB |  |  |  |  |

| **ampC** | | | | |
| --- | --- | --- | --- | --- |
| No known mutations found in ampC |  |  |  |  |

| **23S** | | | | |
| --- | --- | --- | --- | --- |
| No known mutations found in 23S |  |  |  |  |

| **parC** | | | | |
| --- | --- | --- | --- | --- |
| **Mutation** | **Nucleotide change** | **Amino acid change** | **Resistance** | **PMID** |
| parC p.S80I | AGC ➝ ATT | S ➝ I | Nalidixic acid,Ciprofloxacin | [8851598](http://www.ncbi.nlm.nih.gov/pubmed/8851598) |
| parC p.E84V | GAA ➝ GTA | E ➝ V | Nalidixic acid,Ciprofloxacin | [12654733](http://www.ncbi.nlm.nih.gov/pubmed/12654733) |

| **16S_rrsC** | | | | |
| --- | --- | --- | --- | --- |
| No known mutations found in 16S_rrsC |  |  |  |  |

| **gyrB** | | | | |
| --- | --- | --- | --- | --- |
| No known mutations found in gyrB |  |  |  |  |

| **gyrA** | | | | |
| --- | --- | --- | --- | --- |
| **Mutation** | **Nucleotide change** | **Amino acid change** | **Resistance** | **PMID** |
| gyrA p.S83L | TCG ➝ TTG | S ➝ L | Nalidixic acid,Ciprofloxacin | [8891148](http://www.ncbi.nlm.nih.gov/pubmed/8891148) |
| gyrA p.D87N | GAC ➝ AAC | D ➝ N | Nalidixic acid,Ciprofloxacin | [12654733](http://www.ncbi.nlm.nih.gov/pubmed/12654733) |

Top of Form

Bottom of Form

Top of Form

Bottom of Form

**CITATIONS**

For publication of results, please cite:


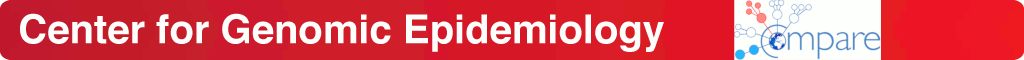


|  | Home | Services | Instructions | Output | Overview of genes | Article abstract |  |
| --- | --- | --- | --- | --- | --- | --- | --- |

**ResFinder-3.2 Server - Results**

**Input Files: *K091.fasta***

Show Acquired antimicrobial resistance results

| No hit found |
| --- |

| **Sulphonamide** | | | | | | |
| --- | --- | --- | --- | --- | --- | --- |
| **Resistance gene** | **Identity** | **Query / Template length** | **Contig** | **Position in contig** | **Predicted phenotype** | **Accession number** |
| sul1 | 100 | 840 / 840 | NXKQ01000037.1 Escherichia coli strain K091 NODE_37_length_11029_cov_64.2095, whole genome shotgun sequence | 3643..4482 | Sulphonamide resistance | [U12338](http://www.ncbi.nlm.nih.gov/nuccore/U12338) |

| **Macrolide** | | | | | | |
| --- | --- | --- | --- | --- | --- | --- |
| **Resistance gene** | **Identity** | **Query / Template length** | **Contig** | **Position in contig** | **Predicted phenotype** | **Accession number** |
| mdf(A) | 97.97 | 1233 / 1233 | NXKQ01000001.1 Escherichia coli strain K091 NODE_1_length_967759_cov_49.018, whole genome shotgun sequence | 344618..345850 | Warning: gene is missing from Notes file. Please inform curator. | [Y08743](http://www.ncbi.nlm.nih.gov/nuccore/Y08743) |

| **Tetracycline** | | | | | | |
| --- | --- | --- | --- | --- | --- | --- |
| **Resistance gene** | **Identity** | **Query / Template length** | **Contig** | **Position in contig** | **Predicted phenotype** | **Accession number** |
| tet(B) | 100 | 1206 / 1206 | NXKQ01000054.1 Escherichia coli strain K091 NODE_54_length_2847_cov_75.404, whole genome shotgun sequence | 1264..2469 | Tetracycline resistance | [AF326777](http://www.ncbi.nlm.nih.gov/nuccore/AF326777) |

| **Aminoglycoside** | | | | | | |
| --- | --- | --- | --- | --- | --- | --- |
| **Resistance gene** | **Identity** | **Query / Template length** | **Contig** | **Position in contig** | **Predicted phenotype** | **Accession number** |
| aadA1 | 100 | 792 / 792 | NXKQ01000037.1 Escherichia coli strain K091 NODE_37_length_11029_cov_64.2095, whole genome shotgun sequence | 2347..3138 | Aminoglycoside resistance | [JX185132](http://www.ncbi.nlm.nih.gov/nuccore/JX185132) |

| **Beta-lactam** | | | | | | |
| --- | --- | --- | --- | --- | --- | --- |
| **Resistance gene** | **Identity** | **Query / Template length** | **Contig** | **Position in contig** | **Predicted phenotype** | **Accession number** |
| blaCTX-M-15 | 100 | 876 / 876 | NXKQ01000041.1 Escherichia coli strain K091 NODE_41_length_8179_cov_80.6043, whole genome shotgun sequence | 3863..4738 | Beta-lactam resistance Alternate name; UOE-1 | [AY044436](http://www.ncbi.nlm.nih.gov/nuccore/AY044436) |
| blaTEM-1B | 100 | 861 / 861 | NXKQ01000041.1 Escherichia coli strain K091 NODE_41_length_8179_cov_80.6043, whole genome shotgun sequence | 181..1041 | Beta-lactam resistance Alternate name; RblaTEM-1 | [AY458016](http://www.ncbi.nlm.nih.gov/nuccore/AY458016) |

| **Trimethoprim** | | | | | | |
| --- | --- | --- | --- | --- | --- | --- |
| **Resistance gene** | **Identity** | **Query / Template length** | **Contig** | **Position in contig** | **Predicted phenotype** | **Accession number** |
| dfrA1 | 99.79 | 474 / 474 | NXKQ01000037.1 Escherichia coli strain K091 NODE_37_length_11029_cov_64.2095, whole genome shotgun sequence | 1781..2254 | Trimethoprim resistance | [AF203818](http://www.ncbi.nlm.nih.gov/nuccore/AF203818) |
| dfrA1 | 99.79 | 474 / 474 | NXKQ01000037.1 Escherichia coli strain K091 NODE_37_length_11029_cov_64.2095, whole genome shotgun sequence | 1781..2254 | Trimethoprim resistance | [AJ238350](http://www.ncbi.nlm.nih.gov/nuccore/AJ238350) |
| dfrA1 | 99.79 | 474 / 474 | NXKQ01000037.1 Escherichia coli strain K091 NODE_37_length_11029_cov_64.2095, whole genome shotgun sequence | 1781..2254 | Trimethoprim resistance | [X00926](http://www.ncbi.nlm.nih.gov/nuccore/X00926) |

Show Point mutation results

**Chromosomal point mutations - Results**

**Species:*escherichia_coli***

**Known Mutations**

| **gyrA** | | | | |
| --- | --- | --- | --- | --- |
| No known mutations found in gyrA |  |  |  |  |

| **gyrB** | | | | |
| --- | --- | --- | --- | --- |
| No known mutations found in gyrB |  |  |  |  |

| **16S_rrsC** | | | | |
| --- | --- | --- | --- | --- |
| No known mutations found in 16S_rrsC |  |  |  |  |

| **16S_rrsH** | | | | |
| --- | --- | --- | --- | --- |
| No known mutations found in 16S_rrsH |  |  |  |  |

| **pmrA** | | | | |
| --- | --- | --- | --- | --- |
| No known mutations found in pmrA |  |  |  |  |

| **folP** | | | | |
| --- | --- | --- | --- | --- |
| No known mutations found in folP |  |  |  |  |

| **parC** | | | | |
| --- | --- | --- | --- | --- |
| No known mutations found in parC |  |  |  |  |

| **parE** | | | | |
| --- | --- | --- | --- | --- |
| No known mutations found in parE |  |  |  |  |

| **ampC** | | | | |
| --- | --- | --- | --- | --- |
| No mutations found in ampC |  |  |  |  |

| **23S** | | | | |
| --- | --- | --- | --- | --- |
| No known mutations found in 23S |  |  |  |  |

| **pmrB** | | | | |
| --- | --- | --- | --- | --- |
| No known mutations found in pmrB |  |  |  |  |

| **rpoB** | | | | |
| --- | --- | --- | --- | --- |
| No mutations found in rpoB |  |  |  |  |

| **16S_rrsB** | | | | |
| --- | --- | --- | --- | --- |
| No known mutations found in 16S_rrsB |  |  |  |  |

Top of Form


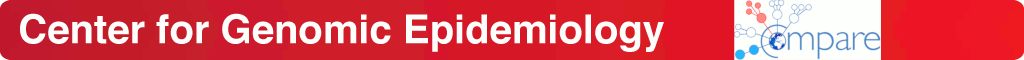


|  | Home | Services | Instructions | Output | Overview of genes | Article abstract |  |
| --- | --- | --- | --- | --- | --- | --- | --- |

**ResFinder-3.2 Server - Results**

**Input Files: *K075.fasta***

Show Acquired antimicrobial resistance results

**Acquired antimicrobial resistance gene - Results**

| **Macrolide** | | | | | | |
| --- | --- | --- | --- | --- | --- | --- |
| **Resistance gene** | **Identity** | **Query / Template length** | **Contig** | **Position in contig** | **Predicted phenotype** | **Accession number** |
| mdf(A) | 98.3 | 1233 / 1233 | NXKJ01000001.1 Escherichia coli strain ST-648:K075 NODE_1_length_423633_cov_194.917, whole genome shotgun sequence | 107394..108626 | Warning: gene is missing from Notes file. Please inform curator. | [Y08743](http://www.ncbi.nlm.nih.gov/nuccore/Y08743) |

| **Beta-lactam** | | | | | | |
| --- | --- | --- | --- | --- | --- | --- |
| **Resistance gene** | **Identity** | **Query / Template length** | **Contig** | **Position in contig** | **Predicted phenotype** | **Accession number** |
| blaCTX-M-14 | 100 | 876 / 876 | NXKJ01000143.1 Escherichia coli strain ST-648:K075 NODE_143_length_1484_cov_445.971, whole genome shotgun sequence | 106..981 | Beta-lactam resistance Amino acid sequences of CTX-M-14 and CTX-M-18 are identical | [AF252622](http://www.ncbi.nlm.nih.gov/nuccore/AF252622) |

| **Sulphonamide** | | | | | | |
| --- | --- | --- | --- | --- | --- | --- |
| **Resistance gene** | **Identity** | **Query / Template length** | **Contig** | **Position in contig** | **Predicted phenotype** | **Accession number** |
| sul3 | 100 | 792 / 792 | NXKJ01000111.1 Escherichia coli strain ST-648:K075 NODE_111_length_2847_cov_292.201, whole genome shotgun sequence | 1927..2718 | Sulphonamide resistance | [AJ459418](http://www.ncbi.nlm.nih.gov/nuccore/AJ459418) |

| **Phenicol** | | | | | | |
| --- | --- | --- | --- | --- | --- | --- |
| **Resistance gene** | **Identity** | **Query / Template length** | **Contig** | **Position in contig** | **Predicted phenotype** | **Accession number** |
| cmlA1 | 99.92 | 1260 / 1260 | NXKJ01000078.1 Escherichia coli strain ST-648:K075 NODE_78_length_7787_cov_385.817, whole genome shotgun sequence | 3779..5038 | Phenicol resistance | [M64556](http://www.ncbi.nlm.nih.gov/nuccore/M64556) |

| **Tetracycline** | | | | | | |
| --- | --- | --- | --- | --- | --- | --- |
| **Resistance gene** | **Identity** | **Query / Template length** | **Contig** | **Position in contig** | **Predicted phenotype** | **Accession number** |
| tet(A) | 100 | 1200 / 1200 | NXKJ01000096.1 Escherichia coli strain ST-648:K075 NODE_96_length_3812_cov_207.436, whole genome shotgun sequence | 1504..2703 | Tetracycline resistance | [AJ517790](http://www.ncbi.nlm.nih.gov/nuccore/AJ517790) |

| **Fosfomycin** | | | | | | |
| --- | --- | --- | --- | --- | --- | --- |
| **Resistance gene** | **Identity** | **Query / Template length** | **Contig** | **Position in contig** | **Predicted phenotype** | **Accession number** |
| fosA3 | 100 | 417 / 417 | NXKJ01000122.1 Escherichia coli strain ST-648:K075 NODE_122_length_2202_cov_421.231, whole genome shotgun sequence | 311..727 | Fosfomycin resistance | [AB522970](http://www.ncbi.nlm.nih.gov/nuccore/AB522970) |

| **Aminoglycoside** | | | | | | |
| --- | --- | --- | --- | --- | --- | --- |
| **Resistance gene** | **Identity** | **Query / Template length** | **Contig** | **Position in contig** | **Predicted phenotype** | **Accession number** |
| aadA1 | 100 | 792 / 792 | NXKJ01000078.1 Escherichia coli strain ST-648:K075 NODE_78_length_7787_cov_385.817, whole genome shotgun sequence | 5131..5922 | Aminoglycoside resistance | [JQ414041](http://www.ncbi.nlm.nih.gov/nuccore/JQ414041) |
| aadA2b | 99.87 | 780 / 780 | NXKJ01000078.1 Escherichia coli strain ST-648:K075 NODE_78_length_7787_cov_385.817, whole genome shotgun sequence | 2738..3517 | Warning: gene is missing from Notes file. Please inform curator. | [D43625](http://www.ncbi.nlm.nih.gov/nuccore/D43625) |
| aph(3'')-Ib | 100 | 803 / 804 | NXKJ01000083.1 Escherichia coli strain ST-648:K075 NODE_83_length_7307_cov_314.041, whole genome shotgun sequence | 1143..1945 | Aminoglycoside resistance Alternate name; aph(3'')-Ib | [AF024602](http://www.ncbi.nlm.nih.gov/nuccore/AF024602) |
| aph(3'')-Ib | 99.88 | 804 / 804 | NXKJ01000083.1 Escherichia coli strain ST-648:K075 NODE_83_length_7307_cov_314.041, whole genome shotgun sequence | 1142..1945 | Aminoglycoside resistance Alternate name; aph(3'')-Ib | [AF313472](http://www.ncbi.nlm.nih.gov/nuccore/AF313472) |
| aph(3'')-Ib | 99.88 | 804 / 804 | NXKJ01000083.1 Escherichia coli strain ST-648:K075 NODE_83_length_7307_cov_314.041, whole genome shotgun sequence | 1142..1945 | Aminoglycoside resistance Alternate name; aph(3'')-Ib | [AF321550](http://www.ncbi.nlm.nih.gov/nuccore/AF321550) |
| aph(3'')-Ib | 99.88 | 804 / 804 | NXKJ01000083.1 Escherichia coli strain ST-648:K075 NODE_83_length_7307_cov_314.041, whole genome shotgun sequence | 1142..1945 | Aminoglycoside resistance Alternate name; aph(3'')-Ib | [AF321551](http://www.ncbi.nlm.nih.gov/nuccore/AF321551) |
| aph(3')-IIa | 100 | 795 / 795 | NXKJ01000094.1 Escherichia coli strain ST-648:K075 NODE_94_length_4203_cov_289.913, whole genome shotgun sequence | 298..1092 | Aminoglycoside resistance | [V00618](http://www.ncbi.nlm.nih.gov/nuccore/V00618) |
| aph(6)-Id | 100 | 837 / 837 | NXKJ01000083.1 Escherichia coli strain ST-648:K075 NODE_83_length_7307_cov_314.041, whole genome shotgun sequence | 1945..2781 | Aminoglycoside resistance Alternate name; aph(6)-Id | [M28829](http://www.ncbi.nlm.nih.gov/nuccore/M28829) |

Show Point mutation results

**Chromosomal point mutations - Results**

**Species:*escherichia_coli***

**Known Mutations**

| **16S_rrsC** | | | | |
| --- | --- | --- | --- | --- |
| No known mutations found in 16S_rrsC |  |  |  |  |

| **rpoB** | | | | |
| --- | --- | --- | --- | --- |
| No mutations found in rpoB |  |  |  |  |

| **folP** | | | | |
| --- | --- | --- | --- | --- |
| No mutations found in folP |  |  |  |  |

| **ampC** | | | | |
| --- | --- | --- | --- | --- |
| No known mutations found in ampC |  |  |  |  |

| **parE** | | | | |
| --- | --- | --- | --- | --- |
| No known mutations found in parE |  |  |  |  |

| **16S_rrsB** | | | | |
| --- | --- | --- | --- | --- |
| No known mutations found in 16S_rrsB |  |  |  |  |

| **pmrB** | | | | |
| --- | --- | --- | --- | --- |
| No known mutations found in pmrB |  |  |  |  |

| **parC** | | | | |
| --- | --- | --- | --- | --- |
| No known mutations found in parC |  |  |  |  |

| **16S_rrsH** | | | | |
| --- | --- | --- | --- | --- |
| No known mutations found in 16S_rrsH |  |  |  |  |

| **gyrB** | | | | |
| --- | --- | --- | --- | --- |
| No known mutations found in gyrB |  |  |  |  |

| **gyrA** | | | | |
| --- | --- | --- | --- | --- |
| **Mutation** | **Nucleotide change** | **Amino acid change** | **Resistance** | **PMID** |
| gyrA p.S83L | TCG ➝ TTG | S ➝ L | Nalidixic acid,Ciprofloxacin | [8891148](http://www.ncbi.nlm.nih.gov/pubmed/8891148) |

| **pmrA** | | | | |
| --- | --- | --- | --- | --- |
| No mutations found in pmrA |  |  |  |  |

| **23S** | | | | |
| --- | --- | --- | --- | --- |
| No known mutations found in 23S |  |  |  |  |

Top of Form

Bottom of Form

Top of Form

Contact: Vibeke Dybdahl Hammer, Telephone: +45 3588 6420, E-mail: vdha@food.dtu.dk

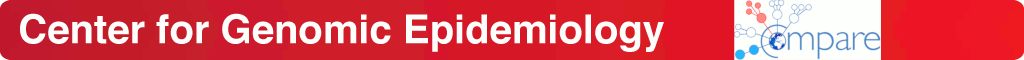


**ResFinder-3.2 Server - Results**

**Input Files: *E013.fasta***

Show Acquired antimicrobial resistance results

**Acquired antimicrobial resistance gene - Results**

| **Trimethoprim** |
| --- |

| **Macrolide** | | | | | | |
| --- | --- | --- | --- | --- | --- | --- |
| **Resistance gene** | **Identity** | **Query / Template length** | **Contig** | **Position in contig** | **Predicted phenotype** | **Accession number** |
| mdf(A) | 97.81 | 1233 / 1233 | NXIN01000013.1 Escherichia coli strain ST-131:E013 NODE_13_length_151384_cov_116.767_ID_25, whole genome shotgun sequence | 50388..51620 | Warning: gene is missing from Notes file. Please inform curator. | [Y08743](http://www.ncbi.nlm.nih.gov/nuccore/Y08743) |

| **Aminoglycoside** | | | | | | |
| --- | --- | --- | --- | --- | --- | --- |
| **Resistance gene** | **Identity** | **Query / Template length** | **Contig** | **Position in contig** | **Predicted phenotype** | **Accession number** |
| aph(3'')-Ib | 100 | 804 / 804 | NXIN01000060.1 Escherichia coli strain ST-131:E013 NODE_60_length_5812_cov_101.321_ID_119, whole genome shotgun sequence | 3553..4356 | Aminoglycoside resistance Alternate name; aph(3'')-Ib | [AF321551](http://www.ncbi.nlm.nih.gov/nuccore/AF321551) |
| aph(6)-Id | 100 | 831 / 831 | NXIN01000060.1 Escherichia coli strain ST-131:E013 NODE_60_length_5812_cov_101.321_ID_119, whole genome shotgun sequence | 2717..3547 | Aminoglycoside resistance Alternate name; aph(6)-Id | [CP000971](http://www.ncbi.nlm.nih.gov/nuccore/CP000971) |

| **Tetracycline** | | | | | | |
| --- | --- | --- | --- | --- | --- | --- |
| **Resistance gene** | **Identity** | **Query / Template length** | **Contig** | **Position in contig** | **Predicted phenotype** | **Accession number** |
| tet(A) | 100 | 1200 / 1200 | NXIN01000060.1 Escherichia coli strain ST-131:E013 NODE_60_length_5812_cov_101.321_ID_119, whole genome shotgun sequence | 464..1663 | Tetracycline resistance | [AJ517790](http://www.ncbi.nlm.nih.gov/nuccore/AJ517790) |

| **Sulphonamide** | | | | | | |
| --- | --- | --- | --- | --- | --- | --- |
| **Resistance gene** | **Identity** | **Query / Template length** | **Contig** | **Position in contig** | **Predicted phenotype** | **Accession number** |
| sul2 | 100 | 816 / 816 | NXIN01000060.1 Escherichia coli strain ST-131:E013 NODE_60_length_5812_cov_101.321_ID_119, whole genome shotgun sequence | 4417..5232 | Sulphonamide resistance | [AY034138](http://www.ncbi.nlm.nih.gov/nuccore/AY034138) |

| **Beta-lactam** | | | | | | |
| --- | --- | --- | --- | --- | --- | --- |
| **Resistance gene** | **Identity** | **Query / Template length** | **Contig** | **Position in contig** | **Predicted phenotype** | **Accession number** |
| blaCTX-M-27 | 100 | 876 / 876 | NXIN01000079.1 Escherichia coli strain ST-131:E013 NODE_79_length_1717_cov_92.1156_ID_157, whole genome shotgun sequence | 493..1368 | Beta-lactam resistance | [AY156923](http://www.ncbi.nlm.nih.gov/nuccore/AY156923) |

extended output

Show Point mutation results

**Chromosomal point mutations - Results**

**Species:*escherichia_coli***

**Known Mutations**

| **16S_rrsC** | | | | |
| --- | --- | --- | --- | --- |
| No known mutations found in 16S_rrsC |  |  |  |  |

| **parE** | | | | |
| --- | --- | --- | --- | --- |
| **Mutation** | **Nucleotide change** | **Amino acid change** | **Resistance** | **PMID** |
| parE p.I529L | ATT ➝ CTT | I ➝ L | Nalidixic acid,Ciprofloxacin | [14506034](http://www.ncbi.nlm.nih.gov/pubmed/14506034) |

| **16S_rrsB** | | | | |
| --- | --- | --- | --- | --- |
| No known mutations found in 16S_rrsB |  |  |  |  |

| **gyrA** | | | | |
| --- | --- | --- | --- | --- |
| **Mutation** | **Nucleotide change** | **Amino acid change** | **Resistance** | **PMID** |
| gyrA p.S83L | TCG ➝ TTG | S ➝ L | Nalidixic acid,Ciprofloxacin | [8891148](http://www.ncbi.nlm.nih.gov/pubmed/8891148) |
| gyrA p.D87N | GAC ➝ AAC | D ➝ N | Nalidixic acid,Ciprofloxacin | [12654733](http://www.ncbi.nlm.nih.gov/pubmed/12654733) |

| **16S_rrsH** | | | | |
| --- | --- | --- | --- | --- |
| No known mutations found in 16S_rrsH |  |  |  |  |

| **rpoB** | | | | |
| --- | --- | --- | --- | --- |
| No mutations found in rpoB |  |  |  |  |

| **folP** | | | | |
| --- | --- | --- | --- | --- |
| No known mutations found in folP |  |  |  |  |

| **pmrB** | | | | |
| --- | --- | --- | --- | --- |
| No known mutations found in pmrB |  |  |  |  |

| **23S** | | | | |
| --- | --- | --- | --- | --- |
| No known mutations found in 23S |  |  |  |  |

| **pmrA** | | | | |
| --- | --- | --- | --- | --- |
| No known mutations found in pmrA |  |  |  |  |

| **gyrB** | | | | |
| --- | --- | --- | --- | --- |
| No known mutations found in gyrB |  |  |  |  |

| **ampC** | | | | |
| --- | --- | --- | --- | --- |
| No known mutations found in ampC |  |  |  |  |

| **parC** | | | | |
| --- | --- | --- | --- | --- |
| **Mutation** | **Nucleotide change** | **Amino acid change** | **Resistance** | **PMID** |
| parC p.S80I | AGC ➝ ATT | S ➝ I | Nalidixic acid,Ciprofloxacin | [8851598](http://www.ncbi.nlm.nih.gov/pubmed/8851598) |
| parC p.E84V | GAA ➝ GTA | E ➝ V | Nalidixic acid,Ciprofloxacin | [12654733](http://www.ncbi.nlm.nih.gov/pubmed/12654733) |

Top of Form

Bottom of Form

Top of Form

Bottom of Form

**CITATIONS**

For publication of results, please cite:

- Identification of acquired antimicrobial resistance genes.
  Zankari E, Hasman H, Cosentino S, Vestergaard M, Rasmussen S, Lund O, Aarestrup FM, Larsen MV.
  J Antimicrob Chemother. 2012 Jul 10.
  PMID: [22782487](http://www.ncbi.nlm.nih.gov/pubmed/22782487)         doi: [10.1093/jac/dks261](http://dx.doi.org/10.1093/jac/dks261)

Top of Form
